# Supplementary material for: Automatic mapping of multiplexed social receptive fields by deep learning and GPU-accelerated 3D videography
Source: Nat Commun. 2022 Feb 1;13:593. doi: 10.1038/s41467-022-28153-7 (PMC8807631; doi:10.1038/s41467-022-28153-7)
Supplement: Supplementary file 2 — Reporting Summary [file 41467_2022_28153_MOESM2_ESM.pdf]

nature portfolio

Corresponding author(s): Christian L. Ebbesen

Last updated by author(s): Dec 14, 2021

Reporting Summary

Nature Portfolio wishes to improve the reproducibility of the work that we publish. This form provides structure for consistency and transparency in reporting. For further information on Nature Portfolio policies, see our Editorial Policies and the Editorial Policy Checklist.

Statistics

For all statistical analyses, confirm that the following items are present in the figure legend, table legend, main text, or Methods section.

n/a	Confirmed	
☐	☒	The exact sample size (*n*) for each experimental group/condition, given as a discrete number and unit of measurement
☒	☐	A statement on whether measurements were taken from distinct samples or whether the same sample was measured repeatedly
☐	☒	The statistical test(s) used AND whether they are one- or two-sided<br>*Only common tests should be described solely by name; describe more complex techniques in the Methods section.*
☐	☒	A description of all covariates tested
☐	☒	A description of any assumptions or corrections, such as tests of normality and adjustment for multiple comparisons
☐	☒	A full description of the statistical parameters including central tendency (e.g. means) or other basic estimates (e.g. regression coefficient) AND variation (e.g. standard deviation) or associated estimates of uncertainty (e.g. confidence intervals)
☐	☒	For null hypothesis testing, the test statistic (e.g. *F*, *t*, *r*) with confidence intervals, effect sizes, degrees of freedom and *P* value noted<br>*Give P values as exact values whenever suitable.*
☐	☒	For Bayesian analysis, information on the choice of priors and Markov chain Monte Carlo settings
☐	☒	For hierarchical and complex designs, identification of the appropriate level for tests and full reporting of outcomes
☐	☒	Estimates of effect sizes (e.g. Cohen's *d*, Pearson's *r*), indicating how they were calculated

Our web collection on statistics for biologists contains articles on many of the points above.

Software and code

Policy information about availability of computer code

Data collection	Python 3.6 (packages installed with pip and conda), Intel Realsense SDK 2.0 , Arduino 1.6.3, Ubuntu 16.04 LTE. Our custom recording code is avaiable at https://github.com/chrelli/3DDD_social_mouse_tracker/
Data analysis	Python 3.6 (packages installed with pip and conda), R v. 3.5.3 (packages installed from CRAN). Data analysis functions and scripts are available at https://github.com/chrelli/3DDD_social_mouse_tracker/

For manuscripts utilizing custom algorithms or software that are central to the research but not yet described in published literature, software must be made available to editors and reviewers. We strongly encourage code deposition in a community repository (e.g. GitHub). See the Nature Portfolio guidelines for submitting code & software for further information.

Data

Policy information about availability of data

All manuscripts must include a data availability statement. This statement should provide the following information, where applicable:

- Accession codes, unique identifiers, or web links for publicly available datasets
- A description of any restrictions on data availability
- For clinical datasets or third party data, please ensure that the statement adheres to our policy

An example dataset with source data for the figures (and raw data) is available on Zenodo: https://doi.org/10.5281/zenodo.5790820

Field-specific reporting

Please select the one below that is the best fit for your research. If you are not sure, read the appropriate sections before making your selection.

- [x] Life sciences
- [ ] Behavioural & social sciences
- [ ] Ecological, evolutionary & environmental sciences

For a reference copy of the document with all sections, see nature.com/documents/nr-reporting-summary-flat.pdf

Life sciences study design

All studies must disclose on these points even when the disclosure is negative.

Sample size	No sample size calculation was performed, we report the performance of the method by reporting all neurons that we could record per animal.
Data exclusions	None.
Replication	All replication attempts were succesfull, no exclusion of replication attempts.
Randomization	None. There was no assignment to experimental groups.
Blinding	None. There was no assignment to experimental groups.

Reporting for specific materials, systems and methods

We require information from authors about some types of materials, experimental systems and methods used in many studies. Here, indicate whether each material, system or method listed is relevant to your study. If you are not sure if a list item applies to your research, read the appropriate section before selecting a response.

Materials & experimental systems

n/a	Involved in the study	
x		Antibodies
x		Eukaryotic cell lines
x		Palaeontology and archaeology
	x	Animals and other organisms
x		Human research participants
x		Clinical data
x		Dual use research of concern

Methods

n/a	Involved in the study	
x		ChIP-seq
x		Flow cytometry
x		MRI-based neuroimaging

Animals and other organisms

Policy information about studies involving animals; ARRIVE guidelines recommended for reporting animal research

Laboratory animals	C57BL/6J mice (Jackson Laboratory), males (implanted), males and females (interaction partners), all adults (age 3-4 months).
Wild animals	None.
Field-collected samples	None.
Ethics oversight	All experimental procedures were performed according to animal welfare laws under the supervision of local ethics committees. All procedures were approved under NYU School of Medicine IACUC protocols.

Note that full information on the approval of the study protocol must also be provided in the manuscript.
