# Supplementary material for: Automatic mapping of multiplexed social receptive fields by deep learning and GPU-accelerated 3D videography
Source: Nat Commun. 2022 Feb 1;13:593. doi: 10.1038/s41467-022-28153-7 (PMC8807631; doi:10.1038/s41467-022-28153-7)
Supplement: Supplementary file 3 — Description of Additional Supplementary Files [file 41467_2022_28153_MOESM3_ESM.pdf]

Title: Supplementary Movie 1.
Description: Preprocessing pipeline. Example video sequence showing raw RGB and depth video, detected part affinity fields and body keypoints, 3D alignment and final pre-processed data.

Title: Supplementary Movie 2.
Description: Particle filter behaviour. Example convergence sequence of the multi-body particle filter on a single frame of pre-processed 3D data.

Title: Supplementary Movie 3.
Description: Statespace filtering. Example video sequence showing raw (left side) and state-space filtered (right side) behaviour data.

Title: Supplementary Movie 4.
Description: Social events. Examples of the three types of detected social events: nose-to-nose touch (left), anogenital sniffing by the implanted animal (center), and anogenital sniffing by the partner animals (right).

Title: Supplementary Movie 5.
Description: MousePlayer. Video sequence showing how to launch and use the interactive „MousePlayer‟ to play back and inspect pre-processed data and tracked data.

Title: Supplementary Software.
Description: *.zip file containing data recording software code (python), analysis scripts (jupyter notebooks), and associated readme files and step-bystep guides
