# Supplementary material for: Automatic mapping of multiplexed social receptive fields by deep learning and GPU-accelerated 3D videography
Source: Nat Commun. 2022 Feb 1;13:593. doi: 10.1038/s41467-022-28153-7 (PMC8807631; doi:10.1038/s41467-022-28153-7)
Supplement: Supplementary file 9 — Supplementary Software [file 41467_2022_28153_MOESM9_ESM.zip › ebbesen_froemke_2021_code/README_install.pdf]

Installing required software

OS versions

Running on `Ubuntu 16.04 LTE`, also semi-tested and should work on `Ubuntu 17.10` and `macOS 10.13`. This guide is for installing and running on linux (recommended).

Installing prerequisites

Update Ubuntu drivers
Update the proprietary Intel CPU and NVIDIA GPU drivers

Install git and clone the code
`$ sudo apt install git`
`$ mkdir git && cd git`

Install curl
`$ sudo apt install curl`

Install Python with anaconda
`$ cd ~/Downloads/`
`$ curl -O https://repo.continuum.io/archive/Anaconda3-5.0.1-Linux-x86_64.sh`
`$ sha256sum Anaconda3-5.0.1-Linux-x86_64.sh`
`$ bash Anaconda3-5.0.1-Linux-x86_64.sh`

Set up python environment

Make new environment with full anaconda3, eg. "myp":
`$ conda update -n base -c defaults conda`
`$ conda create -n myp anaconda python=3.6`
`$ conda install -c conda-forge opencv`
`$ conda install -n myp imgaug`
`$ conda install -n myp numba h5py`
`$ source activate myp`
`$ pip install tqdm colour tensorboard cmocean`
`$ pip install filterpy pyquaternion pywaffle`
`$ pip install palettable` (for the color selection)
`$ pip install future` (fixes caffe error in tensorboard, see https://github.com/pytorch/pytorch/issues/22389)
`$ pip install pyfirmata`
`$ pip install shapely[vectorized]`
`$ pip install git+https://github.com/daavoo/pyntcloud` (we use some RANSAC routines from this)

Install Pytorch (>= 1.0) and Pyro
Details depend on your cuda version, so check the website for details (https://pytorch.org/).
`$ conda install -n myp pytorch torchvision cudatoolkit=10.0 -c pytorch`
`$ pip install pyro-ppl`

Install librealsense and link it to the environment

Install cmake
`$ sudo apt install cmake`

Install and build the librealsense library:
Build the release version for best speed. Very importantly, build it without multiprocessing (openmp). We are running each of the four cameras on separate cpu cores, so the build has to be single-threaded. Otherwise each instance of librealsense will try to spawn multiple processes, choke everything, slow down and not be able to record.
The make flags should be set like this:

```
-DBUILD_EXAMPLES=true
-D CMAKE_BUILD_TYPE=release
-DPYTHON_EXECUTABLE=[full path to your python environment]
-DBUILD_PYTHON_BINDINGS=bool:true
-DBUILD_WITH_OPENMP=false
```

```
$ sudo apt-get update && sudo apt-get upgrade && sudo apt-get dist-upgrade
$ sudo apt-get install git libssl-dev libusb-1.0-0-dev pkg-config libgtk-3-dev
$ sudo apt-get install libglfw3-dev
$ git clone https://github.com/IntelRealSense/librealsense.git
$ cd librealsense
$ mkdir build && cd build
$ cmake ../ -DCMAKE_BUILD_TYPE=release -DBUILD_WITH_OPENMP=false -DBUILD_EXAMPLES=true -DBUILD_PYTHON_BINDINGS=bool:true -DPYTHON_EXECUTABLE=/home/chrelli/anaconda3/envs/myp/bin/python
$ make && sudo make install
```

The librealsense keeps slightly changing, though, so also check https://github.com/IntelRealSense/librealsense/blob/master/doc/installation.md

Note on camera sync:
For the camera sync to work, the four cameras have to be connected by sync cables, as described in this white paper:
https://www.intel.com/content/www/us/en/support/articles/000028140/emerging-technologies/intel-realsense-technology.html
Cam 0 in the code is the Master camera, camera ordering is determined in the code by sorting the cameras based on their serial numbers.
