# Supplementary material for: Automatic mapping of multiplexed social receptive fields by deep learning and GPU-accelerated 3D videography
Source: Nat Commun. 2022 Feb 1;13:593. doi: 10.1038/s41467-022-28153-7 (PMC8807631; doi:10.1038/s41467-022-28153-7)
Supplement: Supplementary file 9 — Supplementary Software [file 41467_2022_28153_MOESM9_ESM.zip › ebbesen_froemke_2021_code/README_analysis.pdf]

Flow of the analysis functions

We provide Jupyter notebooks that walk through all steps and demonstrate a full analysis pipeline. The example data are big files, so they are in a separate zip file. Please unzip that other file as well, and move the example data files as indicated in `README_data.md` in the other zip file.

NB! Note for Referees: We also provide the notebooks as html, so they should be easy to read without having to re-run everything. Some notebooks are written to display the figures inline (matplotlib notebook style), other notebooks use QT5 for better zooming and for more responsive 3D plots. In these notebooks, we have added some PNGs of the plots, so the html versions are still readable.

Example analysis workflow for RGB-based tracking

Network architecture and training

```
`001_Generate a training set`
```

Shows how to use clustering to extract a training image set from a recorded video.

```
`002_Label training images`
```

Shows how to run the GUI to label a training dataset.

```
`003_Train Hourglass network`
```

Shows how to train the hourglass network.

```
`004_Plot network training`
```

Shows various diagnostic plots to verify that training was successful.

Color and depth data pre-processing

```
`005_Process color images`
```

Pre-processing pipeline for the color images: detection of keypoints.

```
`006_Process depth images`
```

Pre-processing pipeline for depth data: 3D deprojection of keypoints, and alignement and stitching of pointcloud.

Actual tracking

```
`007_Tracking engine`
```

Shows how to run the tracking algorithm on a pre-processed dataset.

```
`008_Plot tracked parameters`
```

Shows how to extract and plot tracked parameters.

Behavioral analysis

```
`009_Error checks, analysis of head direction and running behavior`
```

Shows various error checks, diagnostic plots and how to extract 3D pose information.

```
`010_State space modeling and detection of social events`
```

Shows behavioral analysis.

```
`011_Align tracking data to electrophysiology`
```

Shows temporal stability and alignment of tracked behavior data.

Make videos and additional figures for the manuscript

```
`012_Make pre-processing pipeline video`
```

Makes a video showing the pipeline from raw images to pre-processed 3D data.

```
`013_Make supplementary particle filter video`
```

Makes a video showing how the particle filter converges

```
`014_Make Kalman filtering video`
```

Makes a side-by-side video of raw and filtered tracking data, plus some additional supplementary figures.

```
`015_Make example social interaction video`
```

Makes a video showing a few social events.

```
`016_Save a 10s sequence of images`
```

Dump a sequence of images for a figure.

```
`017_Plot example error and artifact`
```

Dump images for manual error inspection, and plot an example sequence showing a real error and a depth artifact.

MousePlayer

We provide an interactive jupyter notebook app for viewing the pre-processed and tracked behavior data. The app should be straightforward to use (see supplementary video 5):

```
`MousePlayer.ipynb`
```

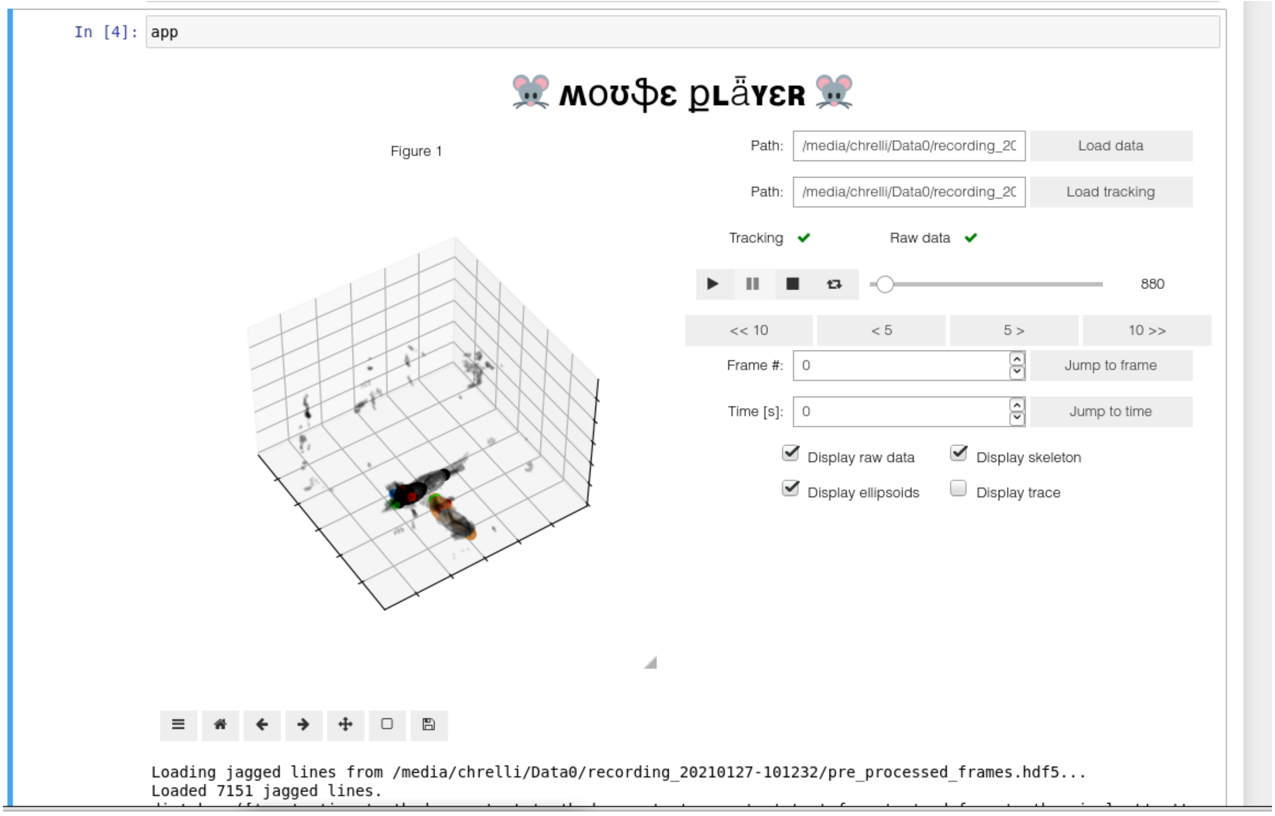

Note! The interactive video plots work in Jupyter Notebook, but not in Jupyter Lab (due to some backend differences between how they handle interactive matplotlib plots)

Automatic tracking (e.g. overnight)

The easiest way to automate the tracking is set everything up in a notebook, and then use a bash script to convert the notebook down to a `.py` file that are run from the terminal. Please see the example script:

```
`overnight_run.sh`
```

Example analysis workflow and profiling of IR-based tracking

We include example workflows for analysis and profiling on the IR-based tracking (reported in the paper). All the notebooks follow the same naming conventions as above, except that they are prepended with `ir_` and `ir_profiling_` for the notebooks where we record and profile IR-only tracking (in mice without a neural implant) and `IMP_ir_` for notebooks, where we record and analyze data from mice with electrophysiology headstages.

Example spike train modeling workflow

We provide four notebooks that walk through all the electrophysiology analysis in the paper.

```
`EPHYS_01_Align_data_and_make_example_plots`
`EPHYS_02_Automated_PSTH_analysis`
`EPHYS_03_Calculate_behavior_features_and_fit_models`
`EPHYS_04_Analyze_fitted_spike_train_models`
```

The notebooks should be pretty straightforward to follow, and the spike train modeling code is easy to modify (add or remove derived behavioral features of interest, for example).
