# Supplementary material for: Automatic mapping of multiplexed social receptive fields by deep learning and GPU-accelerated 3D videography
Source: Nat Commun. 2022 Feb 1;13:593. doi: 10.1038/s41467-022-28153-7 (PMC8807631; doi:10.1038/s41467-022-28153-7)
Supplement: Supplementary file 9 — Supplementary Software [file 41467_2022_28153_MOESM9_ESM.zip › ebbesen_froemke_2021_code/README_recording.pdf]

Flow of the recording functions

All python functions are made to be run from command line, with optional flags. List of options can be accessed with `-h` in the terminal:

```
Run in terminal: `python function_name -h`
```

In the current version, all recording functions dump data to two hard drives (two nVME drives on my lab computer). The user will have to edit these file paths to run the code (The code throws an error for a missing folder -> change line to your preferred recording location).

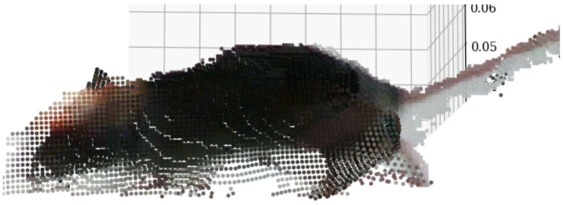

Before starting recording (each time)

Activate the correct python environment:

```
Run in terminal: `source activate env_name`
```

Open the firmata port:

```
Run in terminal: `sudo chmod 666 /dev/ttyACM0` + pw
```

Set up cameras and LEDs (one time)

To check exposure, camera view, alignment, stream live video from all cameras:

```
Run in terminal: `python stream_live_ir.py`
```

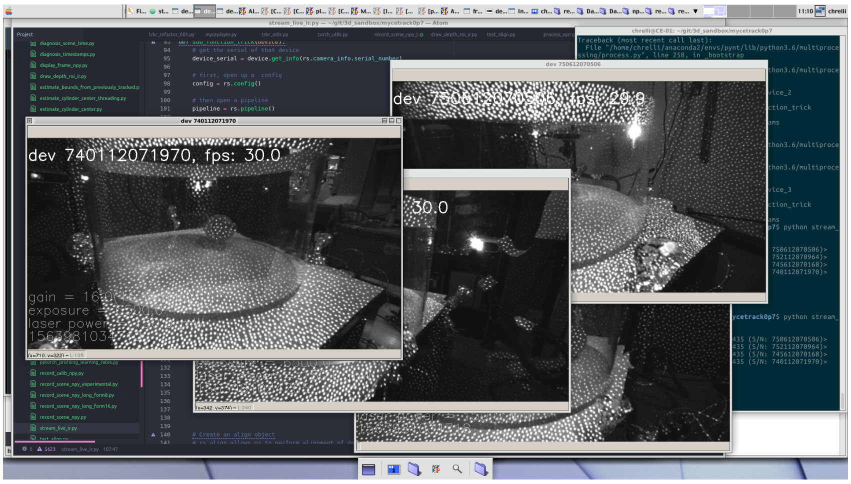

You can choose what to to stream, frame rate, etc by flags (pass `-h`):

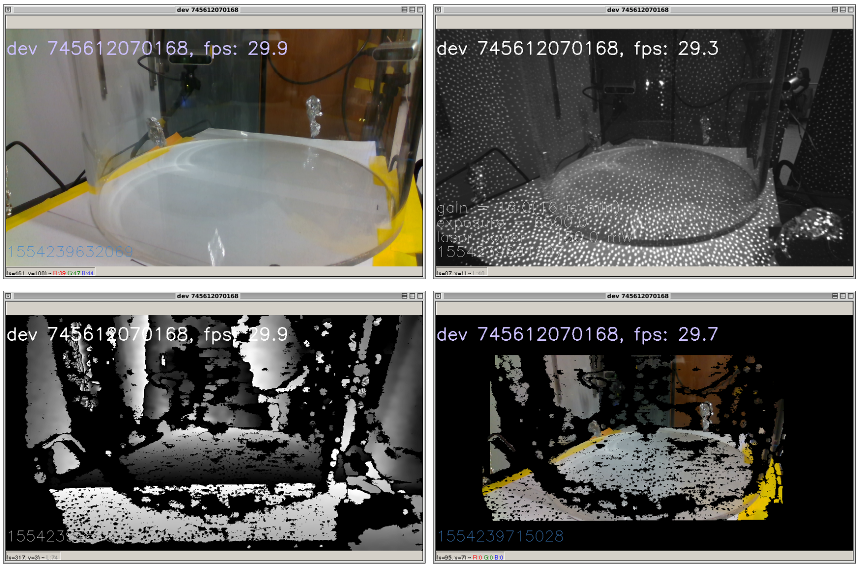

Automatically detect LED ROI by blinking from the Arduino:

```
Run in terminal: `python auto_LED_mask_ir.py`
```

Record and process calibration trace (one time)

Record a calibration trace

This is set up for a blue ping pong ball. Choose a color depending on the wall color of your laboratory, and adjust the HSV filter accordingly.

```
Run in terminal: `python record_calib_npy.py`
```

Check that the time stamps of the calibration trace are stable and that frames are not dropping.

```
Run in terminal: `python diagnosis_calib_time.py`
```

Reasons for dropped frames could be:

- You are using auto-exposure and your lab is too dark, so camera exposure time is getting too long for the frame rate -> Turn on some lamps.
- Your USB cables are bad, or you are not using independent USB controllers for every camera -> Use a dedicated PCIe card with multiple USB controllers and/or use actively repeating USB cables (see our recommendations in the manuscript).
- Your hard drive is too slow -> use an nVME SSD.

Check that the color and motion filtering looks good, adjust sliders if necessary:

```
Run in terminal: `python generate_HSV_filter_for_calibration_npy.py`
```

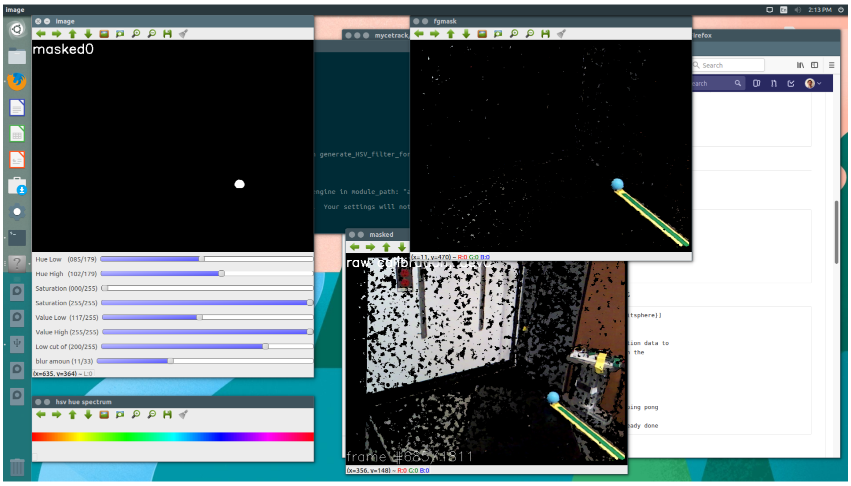

Align the camera data and time stamps

```
Run in terminal: `python align_time_stamps.py --convert calib`
```

Process the calibration data to extract the center location of the ping pong ball in every frame

```
Run in terminal: `python convert_calib_to_trace_npy.py`
```

Have a look to make sure the calibraion trajectory is healthy

```
Run in terminal: `python plot_calib_trace.py`
```

Automatically estimate the arena location and rotation matrix from calibration traces

These will dump the rotation matrices, etc. in the calibration data folder:

```
Run in terminal: `python generate_transformation_matrix.py`
Run in terminal: `python generate_arena_rotation_npy.py`
```

Record actual data (each time)

Record data, color and depth video, at 60 frames/sec:

```
Run in terminal: `python record_scene_npy_long_format_form16.py --nocad --noroi`
```

This dumps the color files as PNG and the depth as uint16 numpy binary files, in these lines:

```
>> cv2.imwrite(npy_folder+'/dev'+str(which_device)+'_cad_'+str(FRAME_CLOCK).rjust(n_padding_digits,'0')+'.png', cad[60::2,:
>> np.save(npy_folder+'/dev'+str(which_device)+'_d_'+str(FRAME_CLOCK).rjust(n_padding_digits,'0')+'.npy',depth, allow_pickl
```

Yielding the following file names:

```
dev0_cad_00000001.png (color frame #1 from cam 0, 8-bit, 3 channel, png-encoding)
dev0_d_00000001.npy (depth frame #1 from cam 0, 16-bit numpy array, binary file)
```

The function is set to pull raw images at 640 x 480 (color) and 640 x 480 (depth), but only saves 320 x 210 (color) and 640 x 480 (depth). We do this to reduce noise (multi-pixel averaging), save disk space and reduce processing time. Our software also works for saving images up to 848 x 480 (color) and 848 x 480 (depth) at 60 frames/s (if you need a higher resolution for some reason).

Optional, but good to do: Check time stamps

Record a short video, then have a quick look that time stamps are stable and that everything is fine. Maybe someone has moved something, an LED has died, or whatever:

```
Run in terminal: `python diagnosis_scene_time.py`
Run in terminal: `python align_time_stamps.py --convert scene`
```

After every recording, prepare for pre-processing

Before running these: Copy the three geometry variables (`M0` , `floor_point` and `floor_vec`) from the calibration folder to the recording folder [todo: We could automate this]. Then automatically detect the location of the acrylic cylinder (this changes with every recording):

```
Run in terminal: `python dump_cylinder_center.py`
```

And pack all the timing and geometry matrices, camera intrinsics, etc to a single, handy file:

```
Run in terminal: `python dump_geometry.py`
```

Differences between RGB-based tracking and Infrared-only tracking

Some of the functions differ between RGB-based tracking and tracking that only uses the IR data. RGB tracking is the default, and IR-recording/processing functions are marked by `_ir` . For example, after recording replace:

```
Run in terminal: `python dump_geometry.py`
```

with:

```
Run in terminal: `python dump_geometry_ir.py`
```

If an IR-specific function is needed, the function will throw an error (since no color data is found on disk).
