# Supplementary material for: Automatic mapping of multiplexed social receptive fields by deep learning and GPU-accelerated 3D videography
Source: Nat Commun. 2022 Feb 1;13:593. doi: 10.1038/s41467-022-28153-7 (PMC8807631; doi:10.1038/s41467-022-28153-7)
Supplement: Supplementary file 9 — Supplementary Software [file 41467_2022_28153_MOESM9_ESM.zip › ebbesen_froemke_2021_code/analysis/005_Process_color_images.html]

005\_Process\_color\_images


In [1]:

```
# MAKE A PIPELINE FOR PROCESSING THE COLOR IMAGES!
# FROM TIRAMISU
# IDEA: Add neck to the posture map?
# %matplotlib inline
# %matplotlib widget
%matplotlib qt
%load_ext autoreload
%autoreload 2

import time
from pathlib import Path
import numpy as np
import matplotlib.pyplot as plt

import torch
import torch.nn as nn
import torch.optim as optim
import torchvision
import torchvision.transforms as transforms

import sys, os, pickle
import cv2
from colour import Color
import h5py
from tqdm import tqdm, tqdm_notebook
```

In [2]:

```
# Check CUDA

print(torch.cuda.is_available())
print(torch.cuda.device_count())
print(torch.cuda.get_device_name(0))
torch_device = torch.device("cuda:0" if torch.cuda.is_available() else "cpu")
print(torch_device)
```

```
True
1
GeForce RTX 2080 Ti
cuda:0
```

# Make the hourglass net¶

In [3]:

```
# import the hourglass model and set up architecture
from architectures.hourglass import hg
global best_acc

model = hg(
    num_stacks=8,
    num_blocks=1,
    num_classes=11,
    num_feats=128,
    inplanes=64,
    init_stride=2,
)
    
model = torch.nn.DataParallel(model).cuda()
```

# Load well-trained weights and apply to the hourglass¶

In [4]:

```
# A HELPER FUNCTION WHICH SAVES THE STATE OF THE NETWORK, maybe every 10 epochs or smth?
import os
import sys
import math
import string
import random
import shutil
import glob
epoch = 50
epoch = 110

# save_weights(model, 0, 1000)    
# def load_weights(model, epoch):
WEIGHTS_PATH = '192x320_weights/'

# # the the most recent from that epoch
# all_options = sorted( glob.glob(WEIGHTS_PATH + '/singlecore_weights_epoch_'+str(epoch)+'*' ) )
# print(all_options)
# weights_fpath = all_options[0]

# CHOSEN
weights_fpath = WEIGHTS_PATH + 'singlecore_weights_epoch_135_loss_1565.910_2019-11-22_11-14-39.pth'
print("loading weights '{}'".format(weights_fpath))
model.load_state_dict( torch.load(weights_fpath) )
model.eval()
print('loaded!')
```

```
loading weights '192x320_weights/singlecore_weights_epoch_135_loss_1565.910_2019-11-22_11-14-39.pth'
loaded!
```

# Wrap images in pytorch dataset and make helper functions¶

In [5]:

```
# load a stacvk of some sorted frames!

import numpy as np
import cv2
import h5py
import matplotlib.pyplot as plt
# from deepposekit import VideoReader, KMeansSampler
import sys
# sys.path.append('/home/chrelli/git/3d_sandbox/mouseposev0p1/chrelli_annotator/')
# sys.path.append('/home/chrelli/git/3d_sandbox/mousepose_0p1/deepposekit-annotator/')

# from dpk_annotator import VideoReader, KMeansSampler
import tqdm
import glob
import itertools

from os.path import expanduser
home = expanduser("~")
```

In [6]:

```
# load the geometry
top_folder_0 = '/media/chrelli/Data0/recording_20190905-115115'
top_folder_1 = '/media/chrelli/Data1/recording_20190905-115115'

scene_folders = [top_folder_0,top_folder_0,top_folder_1,top_folder_1]
import pickle
geometry = pickle.load( open( scene_folders[0]+'/geometry.pkl', "rb" ) ) 
timing = pickle.load( open( scene_folders[0]+'/timing.pkl', "rb" ) )
print(geometry.keys())
print(timing.keys())
```

```
dict_keys(['start_frame', 'end_frame', 'd_cam_params', 'c_cam_params', 'R_extrinsics', 't_extrinsics', 'R_world', 't_world', 'M0', 'floor_point', 'floor_normal', 'c_cylinder', 'r_cylinder'])
dict_keys(['master_frame_table', 'reference_time_cam', 'reference_stamps', 'time_stamps', 'shifted_stamps'])
```

In [7]:

```
# also make a list of all the frames to process
# make a list of the cameras!
png_files = [glob.glob(scene_folders[i] + '/npy_raw/dev' +str(i) +'_cad_*.png') for i in range(4)]
png_files = [sorted(f) for f in png_files]
```

In [8]:

```
# WRAP inside of a pytorch dataset
import torch
import torch.utils.data as data
import imgaug.augmenters as iaa

from c_utils.utils_hour import gaussian

class ReadDataset(data.Dataset):
    # todo add augmentation here, clean up and make faster
    # todo remove stupid side effects etc
    def __init__(self, dev,png_files):
        '''Initialization'''
        self.dev = dev
        self.file_list = png_files[dev]
        self.n_frames = len(self.file_list)
        
    def __len__(self):
        'Denotes the total number of samples'
        return self.n_frames    

    def __getitem__(self, index):
        # returns the image in RBG
        c_image = cv2.imread(self.file_list[index])
        # REMEMBER TO CUT DOWN THE TOP of the revolution, such that the image is 192x320
        # pack depth and pixels to target - OR NOT??
        im = c_image
        frame_height = im.shape[0]
        frame_width = im.shape[1]
        
        # make the resolution correct, i.e. set the height to 192
        pad_top = 8
        pad_bottom = 10
        im = im[pad_top:-pad_bottom,:,:]       
        # return the index AND flip from rgb to bgr AND normalize to 0 to 1
#         return index, np.moveaxis( im[:,:,[2,1,0]], 2, 0)
        return index, np.moveaxis( im, 2, 0)

    
# # we shuffle, so that we always see different dumps
dev = 0
FrameLoader = data.DataLoader( ReadDataset(dev,png_files) , batch_size=12, shuffle=False, num_workers = 1)
```

In [27]:

```
# geneate some frames, show as video to see that it's working!
for _ in range(1):    
#     im,target = random_from( MouseTrainLoader)
    index,im_batch = next(iter( FrameLoader))
    print(im_batch.shape)
    print(im_batch.dtype)
    print(index)
    print(torch.max(im_batch))
    plt.figure(figsize=(6,4))
    for i in range(12):
        plt.subplot(3,4,1+i)
#         plt.imshow(np.moveaxis(im_batch[i,...].numpy(),0,2))
        plt.imshow(np.moveaxis(im_batch[i,...].numpy(),0,2))
        plt.axis('off')
    plt.suptitle('matplotlib wants rgb, network wants bgr \n verify here, i.e. \n these colors must look WEIRD')
    plt.show()
```

```
torch.Size([50, 3, 192, 320])
torch.uint8
tensor([ 0,  1,  2,  3,  4,  5,  6,  7,  8,  9, 10, 11, 12, 13, 14, 15, 16, 17,
        18, 19, 20, 21, 22, 23, 24, 25, 26, 27, 28, 29, 30, 31, 32, 33, 34, 35,
        36, 37, 38, 39, 40, 41, 42, 43, 44, 45, 46, 47, 48, 49])
tensor(255, dtype=torch.uint8)
```

In [12]:

```
# loop over the dataset and generate the score maps!

# function to pass through the network
def im_batch_2_scores(im_batch,model):
    inputs = im_batch.float().div(255.).cuda()
    model.eval()
    with torch.no_grad():
        # compute model output
        output = model(inputs)
        # get the resulting scores out! Drop the affinity maps
        scores = output[-1][:,:4,:,:]
    return scores.cpu().numpy()
```

In [22]:

```
# plot some scores to see that it's fine
sco = im_batch_2_scores(im_batch,model)
plt.figure(figsize=(6,3))

for i in range(12):
    plt.subplot(3,4,1+i)
    plt.imshow(sco[i,1,:,:])
    plt.axis('off')
plt.suptitle('Example network output')
plt.subplots_adjust(hspace=.1,wspace=.1)    
plt.show()
```

In [24]:

```
from skimage.feature import peak_local_max

def single_score_2_keypoints(sco):
    xy_list = [None]*4
    pxy_list = [None]*4
    score_idx_list = [None]*4
    for key in range(4):
        xy = peak_local_max(sco[key,:,:],threshold_abs = 0.5,num_peaks = 6)
        xy_list[key] = xy
        pxy_list[key] = sco[key,xy[:,0],xy[:,1]]
        # print(xy.shape)
        score_idx_list[key] = key * np.ones_like(xy)

    return np.concatenate(xy_list), np.concatenate(pxy_list), np.concatenate(score_idx_list)

def save_keypoints(dev,index,sco,hf_file):
    for i in range(len(index)):
        xy, pxy, score_idx = single_score_2_keypoints(sco[i,...])
        # stack everythong together in a list
        data_string = np.hstack( [xy.ravel(), np.round(pxy*100).astype('int') ,score_idx[:,0]])
        hf_file['dataset'][index[i]] = data_string
```

# Pass all images through the net and save the detected keypoints in hdf5 file¶

In [ ]:

```
# from tqdm.auto import tqdm # to automatically use tqdm notebook
# from tqdm import tqdm_notebook as tqdm

for dev in range(4):
# for dev in [1,2,3]:
    # make the file, if it does not already exist
    hdf5_file_path = top_folder_0+'/keypoints_'+str(dev)+'.hdf5'
    if os.path.exists(hdf5_file_path):
        print(hdf5_file_path+" was already done!")
        continue
    else:
        print("starting "+hdf5_file_path+"...")
        
    # make a frame loader
    FrameLoader = data.DataLoader( ReadDataset(dev,png_files) , batch_size=50, 
                                  shuffle=False, num_workers = 10)
    
    # open the file
    n_pictures = len(png_files[dev])
    with h5py.File(hdf5_file_path, mode='w') as hdf5_file:
        # make the variable length dataset
        dt = h5py.special_dtype(vlen=np.dtype('int'))
        hdf5_file.create_dataset('dataset', (n_pictures,), dtype=dt)
        # save the keypoint to an h5py file by looping over!
        for index,im_batch in tqdm_notebook(FrameLoader):
            sco = im_batch_2_scores(im_batch,model)
            save_keypoints(dev,index,sco,hdf5_file)    
    
    print(hdf5_file_path+" is done!")
```

In [234]:

```
if False:
    # Wuick check to see if the data is in the h5py file
    with h5py.File(top_folder_0+'/keypoints_'+str(dev)+'.hdf5', mode='r') as hdf5_file:
        print(hdf5_file.keys())
        print(len(hdf5_file['dataset']))
        print( hdf5_file['dataset'][500] )
```

```
<KeysViewHDF5 ['dataset']>
74962
[21 50 23 29 22 48 23 50 24 40 58 85 98 81 90  0  1  1  2  3]
```
