# Supplementary material for: Automatic mapping of multiplexed social receptive fields by deep learning and GPU-accelerated 3D videography
Source: Nat Commun. 2022 Feb 1;13:593. doi: 10.1038/s41467-022-28153-7 (PMC8807631; doi:10.1038/s41467-022-28153-7)
Supplement: Supplementary file 9 — Supplementary Software [file 41467_2022_28153_MOESM9_ESM.zip › ebbesen_froemke_2021_code/analysis/013_Make_supplementary_particle_filter_video.html]

013\_Make\_supplementary\_particle\_filter\_video


In [1]:

```
# IDEA: Add neck to the posture map?
from IPython import get_ipython

# QT for movable plots
%load_ext autoreload
%autoreload 2

import time, os, sys, shutil
from utils.fitting_utils import *

# for math and plotting
import pandas as pd
import numpy as np
import scipy as sp
import matplotlib.pyplot as plt
# %matplotlib notebook
# %matplotlib inline

# %matplotlib widget
%matplotlib qt

from itertools import compress # for list selection with logical
from tqdm import tqdm

from multiprocessing import Process

# ALLSO JIT STUFF
from numba import jit, njit

# and pytorch
import torch

import sys, os, pickle

from colour import Color
import h5py
from tqdm import tqdm, tqdm_notebook
import glob
import itertools
```

# Make an example video for the filter fitting¶

In [2]:

```
im_folder = 'figs/example_convergence'
```

In [5]:

```
import cv2

# create a splash screen
# create blank image
b_cut = 120

height =1000 - b_cut
width =1860

fourcc = cv2.VideoWriter_fourcc(*'MP4V')
fps = 4
out = cv2.VideoWriter('videos/supplementary_video_particles.mp4', fourcc, fps, (int(width), int(height)))

img = np.zeros((int(height),int(width), 3), np.uint8)
font = cv2.FONT_HERSHEY_SIMPLEX
font_color = (255, 255, 255)
h = height-.1*height
w = width/2
font_scale = 1.5
thickness = 2
text = 'Ebbesen & Froemke, 2020'
def put_centered_text(img,text,w,h,font, font_scale, font_color, thickness):
    # get boundary of this text
    textsize = cv2.getTextSize(text, font, font_scale, thickness)[0]
    cv2.putText(img, text, (int(w - textsize[0]/2),int(h) ), font, font_scale, font_color, thickness, cv2.LINE_AA)

put_centered_text(img,text,w,h,font, font_scale, font_color, thickness)
put_centered_text(img,'Supplementary video 2: Particle filter behavior',w,.2*height,font, font_scale, font_color, thickness)

for _ in range(10):
    cv2.imshow('frame',img)
    cv2.waitKey(150)
    out.write(img)

i = 1
im_start = cv2.imread(im_folder+'/figure_number_starting_mix.png'.format(i))
im_end = cv2.imread(im_folder+'/figure_number_final_mix.png'.format(i))

# im_end_hsv = cv2.cvtColor(im_end, cv2.COLOR_BGR2HSV)
alp = .3
im_end_a = im_end.astype('float32') *alp + 255*(1-alp)
im_end_a = np.clip(im_end_a,0,255).astype('uint8')

im_start_a = im_start.astype('float32') *alp + 255*(1-alp)
im_start_a = np.clip(im_start_a,0,255).astype('uint8')


img = 255*np.ones((int(height),int(width), 3), np.uint8)
img[:,:1000,:] = im_start[:-b_cut,:,:]
img[:,-1000:,:] = im_end_a[:-b_cut,:,:] 
put_centered_text(img,'Starting guess',500,.1*height,font, font_scale, (0,0,0), thickness)
cc = int((1-alp)*255)
put_centered_text(img,'Estimated fit',width-500,.1*height,font, font_scale, (cc,cc,cc), thickness)


for _ in range(16):
    cv2.imshow('frame',img)
    cv2.waitKey(150)
    out.write(img)

for i in range(33):
    if i%2 == 0:
        pass
    
    im_top = cv2.imread(im_folder+'/figure_number_{:03d}_top.png'.format(i))
    im_side = cv2.imread(im_folder+'/figure_number_{:03d}_side.png'.format(i))
    im_mix = cv2.imread(im_folder+'/figure_number_{:03d}_mix.png'.format(i))
    cut = 190
    im = cv2.hconcat([im_top[:-b_cut,cut:-cut,:],im_mix[:-b_cut,cut:-cut,:],im_side[:-b_cut,cut:-cut,:]])    
    
    font = cv2.FONT_HERSHEY_SIMPLEX
    put_centered_text(im,'Top view',0.5*(1000-2*cut),.13*height,font, font_scale, (0,0,0), thickness)
    put_centered_text(im,'Side view',1.5*(1000-2*cut),.13*height,font, font_scale, (0,0,0), thickness)
    put_centered_text(im,'Side view',2.5*(1000-2*cut),.13*height,font, font_scale, (0,0,0), thickness)

    strings = ['Explore ','Resample','Estimate']
    textsize = cv2.getTextSize('Iteration 1: ' + strings[0], font, font_scale, thickness)[0]
    w = .5*width
    h = 0.07*height
    cv2.putText(im, 'Iteration {:2.0f}: '.format( 1+(i-i%3)/3) +strings[i%3] , (int(w - textsize[0]/2),int(h) ), font, font_scale, (0,0,0), thickness, cv2.LINE_AA)

    cv2.imshow('frame',im)
    cv2.waitKey(150)
    out.write(im)

    
img = 255*np.ones((int(height),int(width), 3), np.uint8)
img[:,:1000,:] = im_start_a[:-b_cut,:,:]
img[:,-1000:,:] = im_end[:-b_cut,:,:] 
cc = int((1-alp)*255)
put_centered_text(img,'Starting guess',500,.1*height,font, font_scale, (cc,cc,cc), thickness)
put_centered_text(img,'Estimated fit',width-500,.1*height,font, font_scale, (0,0,0), thickness)
    
for _ in range(16):
    cv2.imshow('frame',img)
    cv2.waitKey(150)
    out.write(img)    
out.release()

cv2.destroyAllWindows()
```

In [ ]:

```

```
