# Supplementary material for: Automatic mapping of multiplexed social receptive fields by deep learning and GPU-accelerated 3D videography
Source: Nat Commun. 2022 Feb 1;13:593. doi: 10.1038/s41467-022-28153-7 (PMC8807631; doi:10.1038/s41467-022-28153-7)
Supplement: Supplementary file 9 — Supplementary Software [file 41467_2022_28153_MOESM9_ESM.zip › ebbesen_froemke_2021_code/analysis/015_Make_example_social_interaction_video.html]

015\_Make\_example\_social\_interaction\_video


In [7]:

```
# IDEA: Add neck to the posture map?
from IPython import get_ipython

# QT for movable plots
%load_ext autoreload
%autoreload 2

import time, os, sys, shutil
from utils.fitting_utils import *

# for math and plotting
import pandas as pd
import numpy as np
import scipy as sp
import matplotlib.pyplot as plt
# %matplotlib notebook
# %matplotlib inline

# %matplotlib widget
%matplotlib qt

from itertools import compress # for list selection with logical
from tqdm import tqdm

from multiprocessing import Process

# ALLSO JIT STUFF
from numba import jit, njit

# and pytorch
import torch

import sys, os, pickle
# import cv2
from colour import Color
import h5py
from tqdm import tqdm, tqdm_notebook
import glob
import itertools
```

```
The autoreload extension is already loaded. To reload it, use:
  %reload_ext autoreload
```

In [8]:

```
from utils.analysis_tools import adjust_spines,cmpl
```

# Load the tracked data¶

In [9]:

```
#load the tracked data!
data_folder = 'example_data/tracking/'

with open(data_folder +'tracked_behavior.pkl', 'rb') as f:
    tracked_behavior = pickle.load(f)
print(tracked_behavior.keys())

# load ALL the frames as jagged lines 
with h5py.File(data_folder+'/pre_processed_frames.hdf5', mode='r') as hdf5_file:
    print(hdf5_file.keys())
    print(len(hdf5_file['dataset']))
    jagged_lines = hdf5_file['dataset'][...]
```

```
dict_keys(['var', 'ivar', 'body_constants', 'start_frame', 'end_frame', 'tracking_holder', 'guessing_holder', 'data_folder'])
<KeysViewHDF5 ['dataset']>
74962
```

In [10]:

```
from utils.cuda_tracking_utils import unpack_from_jagged, cheap4d
# kill first 6 secs of the frames (delay is ~180)
start_frame = 30*60
pos, pos_weights, keyp, pkeyp, ikeyp = unpack_from_jagged(jagged_lines[start_frame])
print(ikeyp)
print(pos.shape)

cheap4d(pos,keyp,ikeyp)
# fig = plt.gcf()
# plt.title("N positions is {}".format(pos.shape))
```

```
[0 1 1 2 3 0 1 1 1 2 2 0 2 0 1 1 1 2 3]
(2986, 3)
```

In [11]:

```
print(tracked_behavior['tracking_holder'].shape)
print(tracked_behavior['var'])
print(tracked_behavior['ivar'])
print(tracked_behavior.keys())
```

```
(18, 73361)
['b', 'c', 's', 'psi', 'theta', 'phi', 'x', 'y', 'z', 'b', 'c', 's', 'theta', 'phi', 'x', 'y', 'z']
['b0', 'c0', 's0', 'psi0', 'theta0', 'phi0', 'x0', 'y0', 'z0', 'b1', 'c1', 's1', 'theta1', 'phi1', 'x1', 'y1', 'z1']
dict_keys(['var', 'ivar', 'body_constants', 'start_frame', 'end_frame', 'tracking_holder', 'guessing_holder', 'data_folder'])
```

# Import the plotter and smooth the data¶

In [12]:

```
from utils.analysis_tools import VideoPlotMachine,PlotMachine
# take a list of frames, calculate body supports and plot as a kind of decaying trail, with some decay and lengthm maybe the center of the nose??
plt.close('all')
Plotter = VideoPlotMachine(tracked_behavior,jagged_lines)
#Plotter.kernel_smoothing(9)
Plotter.better_smoothing()

example_frame = 11800

n_fine = 5
Plotter.make_me(example_frame,view_override = [55.0,90.])
```

```
100%|██████████| 73361/73361 [00:16<00:00, 4331.29it/s]
100%|██████████| 73361/73361 [00:17<00:00, 4293.85it/s]
100%|██████████| 73361/73361 [00:17<00:00, 4300.24it/s]
100%|██████████| 73361/73361 [00:17<00:00, 4254.04it/s]
100%|██████████| 73361/73361 [00:17<00:00, 4288.44it/s]
100%|██████████| 73361/73361 [00:17<00:00, 4314.66it/s]
100%|██████████| 73361/73361 [00:14<00:00, 5098.01it/s]
100%|██████████| 73361/73361 [00:17<00:00, 4312.16it/s]
100%|██████████| 73361/73361 [00:17<00:00, 4290.21it/s]
100%|██████████| 73361/73361 [00:17<00:00, 4289.81it/s]
100%|██████████| 73361/73361 [00:17<00:00, 4287.71it/s]
100%|██████████| 73361/73361 [00:17<00:00, 4173.62it/s]
100%|██████████| 73361/73361 [00:17<00:00, 4146.90it/s]
100%|██████████| 73361/73361 [00:14<00:00, 4932.80it/s]
```

# And make an example video with three side-by-side social events¶

In [ ]:

```
# 63812,10200,12515,6214,19239,19259,59269,7814,20839,15458,33622,54890,44279
```

In [53]:

```
# Make videos for example social events from the figure! (detected in previous notebook)
event_frames = [18969,36343,17100]
event_frames = [63812,10200,12515,6214+1600,19239+1600,19259+1600,59269+1600,7814,20839,15458,33622,54890,44279]
# event_frames = [10200,54890,12515]
# event_frames = [63812,60869,54890]
# event_frames = [18969,62390,61606]

# event_frames = [61606,60869,54890] # good, slightly silly middle one
event_frames = [61606,7814,54890] # 
# event_frames = [18969,15458,33612]

event_name = ['Nose0 <-> Nose1','Nose0 -> Tail1','Nose1 -> Tail0']
```

In [54]:

```
# Plot the examples
plt.close('all')
for i in range(len(event_frames)):    
    Plotter.make_me(event_frames[i],cloud = True,skel='smooth',ellip='smooth',
             trace='smooth',view_override = [55.0,90.+18])
```

In [33]:

```
# now, we make the actual videos. They have three parts:
# - 1) a chunk of time before the social event
pre_time = 6 # frames (half frame rate)
# - 2) a rotation around the social event
# - 3) a chunk of time after the social event
post_time = 6 # frames


video_folder = 'videos'
dpi = 60
# dpi = 60


for event_number in tqdm(range(len(event_frames))):
    # before
    frame_window = np.hstack([np.arange(-pre_time*10,0)]).astype('int')*10
    frame_list = frame_window + event_frames[event_number]
    savepath = video_folder+'/pre_'+str(event_number) + '.mp4'
    if True:
        Plotter.video_me(frame_list=frame_list,cloud = True,skel='smooth',ellip='smooth',
             trace='smooth',savepath = savepath,view_override = [20,50],dpi=dpi,time_offset = -frame_window[0]/60)
    
    # after
    frame_window = np.hstack([np.arange(0,post_time*10)]).astype('int')*10
    frame_list = frame_window + event_frames[event_number]
    savepath = video_folder+'/post_'+str(event_number) + '.mp4'
    if True:
        Plotter.video_me(frame_list=frame_list,cloud = True,skel='smooth',ellip='smooth',
             trace='smooth',savepath = savepath,view_override = [20,50],dpi=dpi)
    
    # rotation
    savepath = video_folder+'/rotation_'+str(event_number) + '.mp4'
    if True:
        Plotter.rotation(frame_list=frame_list,cloud = True,skel='smooth',ellip='smooth',
             trace='smooth',savepath=savepath,view_override = [20,50],dpi=dpi)
    plt.close('all')
```

```
100%|██████████| 3/3 [01:49<00:00, 36.42s/it]
```

In [34]:

```
# merge the videos using opencv, from here: https://gist.github.com/nkint/8576156
import cv2
import os

n_videos = 3
video_folder = 'videos'
video_files = [video_folder+'/pre_{:01d}.mp4'.format(i) for i in range(n_videos)]
caps = [cv2.VideoCapture(video_files[i]) for i in range(n_videos)]

width  = caps[0].get(cv2.CAP_PROP_FRAME_WIDTH)   # float
height = caps[0].get(cv2.CAP_PROP_FRAME_HEIGHT)  # float

b_cut = 12
s_cut = 40

fourcc = cv2.VideoWriter_fourcc(*'MP4V')
out_fps = 15
out = cv2.VideoWriter('videos/supplementary_video_social_recomp.mp4', fourcc, out_fps, (int(n_videos*width-6*s_cut), int(height-b_cut)))

# create a splash screen
# create blank image
img = np.zeros((int(height-b_cut),int(n_videos*width-6*s_cut), 3), np.uint8)
font = cv2.FONT_HERSHEY_SIMPLEX
font_color = (255, 255, 255)
h = height-.1*height
w = (n_videos*width-6*s_cut)/2
font_scale = 1.4
thickness = 2
text = 'Ebbesen & Froemke, 2020'
def put_centered_text(img,text,w,h,font, font_scale, font_color, thickness):
    # get boundary of this text
    textsize = cv2.getTextSize(text, font, font_scale, thickness)[0]
    cv2.putText(img, text, (int(w - textsize[0]/2),int(h) ), font, font_scale, font_color, thickness, cv2.LINE_AA)

put_centered_text(img,text,w,h,font, font_scale, font_color, thickness)
put_centered_text(img,'Supplementary video 4: Social events',w,.2*height,font, font_scale, font_color, thickness)

for _ in range(30):
    cv2.imshow('frame',img)
    cv2.waitKey(10)
    out.write(img)
    

        
# Add annotation!
# centering is not easy, in cv2: https://gist.github.com/xcsrz/8938a5d4a47976c745407fe2788c813a
font = cv2.FONT_HERSHEY_SIMPLEX
font_color = (0,0,0)
h = .07*height
w = width/2
font_scale = 1
thickness = 2


def add_files(video_files,c=(0,0,0)):
    caps = [cv2.VideoCapture(video_files[i]) for i in range(n_videos)]

    while(caps[0].isOpened()):
        ret, frame_0 = caps[0].read()
        ret, frame_1 = caps[1].read()
        ret, frame_2 = caps[2].read()
        if frame_1 is not None:
            stacked_frame = cv2.hconcat([frame_0[:-b_cut,s_cut:-s_cut,:],frame_1[:-b_cut,s_cut:-s_cut,:],frame_2[:-b_cut,s_cut:-s_cut,:]])
            
            
            put_centered_text(stacked_frame,'Nose_0 <-> Nose_1',w - s_cut,h,font, font_scale, font_color, thickness)
            put_centered_text(stacked_frame,'Nose_0 -> Tail_1',w+width - 3* s_cut,h,font, font_scale, font_color, thickness)
            put_centered_text(stacked_frame,'Nose_1 -> Tail_0',w+2*width- 5 * s_cut,h,font, font_scale, font_color, thickness)

            cv2.imshow('frame',stacked_frame)
            cv2.waitKey(10)
            out.write(stacked_frame)
        else:
            break

cc = (10,10,10)            
video_files = [video_folder+'/pre_{:01d}.mp4'.format(i) for i in range(n_videos)]
add_files(video_files, c = cc)     
video_files = [video_folder+'/rotation_{:01d}.mp4'.format(i) for i in range(n_videos)]
add_files(video_files, c= cc)     
video_files = [video_folder+'/post_{:01d}.mp4'.format(i) for i in range(n_videos)]
add_files(video_files,c = cc)             
        
caps[0].release()
caps[1].release()
caps[2].release()
out.release()
cv2.destroyAllWindows()

print ("end.")
```

```
end.
```

In [26]:

```
# Plot the examples
plt.close('all')
for i in range(len(event_frames)):    
    Plotter.make_me(event_frames[i],cloud = True,skel='smooth',ellip='smooth',
             trace='smooth',view_override = [55.0,90.+18])
```

In [ ]:

```
# ALso same some examples for the figure
```

In [56]:

```
%matplotlib inline
event_frames = [18969,7814,33612]
az = 55
eta = .01
views = [[az,0+eta],[az,90+eta],[az,90+eta]]
# Plot the examples
plt.close('all')
for i in range(len(event_frames)):    
#     path = '/home/chrelli/git/3d_sandbox/mouseposev0p2/figure_raw_pics/figure_6/kalman_examples/'+str(i)+'.pdf'
    path =  'figs/social_'+str(i)+'.pdf'
    Plotter.make_me(event_frames[i],cloud = True,skel='smooth',ellip='smooth',
             trace='smooth',view_override =views[i],savepath =
                   path)
```

In [ ]:

```

```
