# Supplementary material for: Automatic mapping of multiplexed social receptive fields by deep learning and GPU-accelerated 3D videography
Source: Nat Commun. 2022 Feb 1;13:593. doi: 10.1038/s41467-022-28153-7 (PMC8807631; doi:10.1038/s41467-022-28153-7)
Supplement: Supplementary file 9 — Supplementary Software [file 41467_2022_28153_MOESM9_ESM.zip › ebbesen_froemke_2021_code/analysis/006_Process_depth_images.html]

006\_Process\_depth\_images


In [1]:

```
# OK, now make a function to process images, collapse to a pointcloud and dump to an h5py file

%matplotlib qt
%load_ext autoreload
%autoreload 2

import time
from pathlib import Path
import numpy as np
import matplotlib.pyplot as plt

import torch

import sys, os, pickle
import cv2
from colour import Color
import h5py
from tqdm import tqdm, tqdm_notebook
import glob
import itertools
```

# Load the timing and geometry, load depth images¶

In [2]:

```
# load the geometry
top_folder_0 = '/media/chrelli/Data0/recording_20190905-115115'
top_folder_1 = '/media/chrelli/Data1/recording_20190905-115115'

scene_folders = [top_folder_0,top_folder_0,top_folder_1,top_folder_1]
import pickle
geometry = pickle.load( open( scene_folders[0]+'/geometry.pkl', "rb" ) ) 
timing = pickle.load( open( scene_folders[0]+'/timing.pkl', "rb" ) )
print(geometry.keys())
print(timing.keys())
```

```
dict_keys(['start_frame', 'end_frame', 'd_cam_params', 'c_cam_params', 'R_extrinsics', 't_extrinsics', 'R_world', 't_world', 'M0', 'floor_point', 'floor_normal', 'c_cylinder', 'r_cylinder'])
dict_keys(['master_frame_table', 'reference_time_cam', 'reference_stamps', 'time_stamps', 'shifted_stamps'])
```

In [3]:

```
# also make a list of all the frames to process
# make a list of the cameras!
d_files = [glob.glob(scene_folders[i] + '/npy_raw/dev' +str(i) +'_d_*.npy') for i in range(4)]
d_files = [sorted(f) for f in d_files]

png_files = [glob.glob(scene_folders[i] + '/npy_raw/dev' +str(i) +'_cad_*.png') for i in range(4)]
png_files = [sorted(f) for f in png_files]
```

In [4]:

```
# load all the keypoint data (since it's so tiny!)
# OK, try to see if the data is in the h5py file
keyp_datasets = []
for dev in range(4):
    with h5py.File(top_folder_0+'/keypoints_'+str(dev)+'.hdf5', mode='r') as hdf5_file:
        print(hdf5_file.keys())
        print(len(hdf5_file['dataset']))
        print( hdf5_file['dataset'][500] )
        keyp_datasets.append(hdf5_file['dataset'][...])
```

```
<KeysViewHDF5 ['dataset']>
74962
[21 50 23 29 22 48 23 50 24 40 58 85 98 81 90  0  1  1  2  3]
<KeysViewHDF5 ['dataset']>
74791
[ 32  35  34  41  22  61  40  33  25  65  31  58  23  50 247  70  95  80
  78  84  85   0   1   1   2   2   3   3]
<KeysViewHDF5 ['dataset']>
74801
[20 19 33 51 31 48 23 17 40 46 30 19 99 73 74 54 76 77  0  1  1  1  2  3]
<KeysViewHDF5 ['dataset']>
74785
[ 13  44  22  10  16  43  17  45  26  23  19  32 163  64  94  77  76  91
   0   1   1   2   3   3]
```

# Define a function to align depth to color images¶

In [5]:

```
def align_d_to_c(d_image,c_image,dev,geometry):
    # todo can be sped up    
    pi,pj = np.where( (d_image>0) ) # simply all
    dij = d_image[pi,pj]

    fx,fy,ppx,ppy,depth_scale,fps,frame_width,frame_height = geometry['d_cam_params'][dev]
    fps,frame_width,frame_height = fps.astype('int'),frame_width.astype('int'),frame_height.astype('int')
    fx_c,fy_c,ppx_c,ppy_c,_,_,_,_ = geometry['c_cam_params'][dev]
    
    # FIX the geometry due to downsample
    # divide the xy because downsample
    fx,fy,ppx,ppy = fx/2,fy/2,ppx/2,ppy/2
    fx_c,fy_c,ppx_c,ppy_c = fx_c/2,fy_c/2,ppx_c/2,ppy_c/2
    frame_width,frame_height = int(frame_width/2),int(frame_height/2)

    
    z_m = dij*depth_scale # +1e-6

    # and now use pinhole cam function to get the x and y
    x_m = (pj - ppx) * z_m / fx
    y_m = (pi - ppy) * z_m / fy    

    # and pack to a stack of positions!
    positions_depth_space = np.vstack((x_m,y_m,z_m)).T    

    # swing the depth positions to the color space
    R_extr = geometry['R_extrinsics'][dev]
    t_extr = geometry['t_extrinsics'][dev]
    positions_color_space = np.einsum('ij,aj->ai',R_extr,positions_depth_space) + t_extr

    # now we can caculate cu and cj, the index in the color frame of each point
    ci = np.round(positions_color_space[:,1] * fy_c / positions_color_space[:,2] + ppy_c)
    cj = np.round(positions_color_space[:,0] * fx_c / positions_color_space[:,2] + ppx_c)

    # make sure that they are good (actually, should probably set to zero outside)
    ci = np.clip(ci,0,frame_height-1).astype(int)
    cj = np.clip(cj,0,frame_width-1).astype(int)    

    # depth aligned to color
    
    dac_image = np.zeros((frame_height,frame_width))
    dac_mask = np.zeros((frame_height,frame_width))
    # return the depth in meters
    dac_image[ci,cj] = dij
    dac_mask[ci,cj] = 1
    sigma_g = 3
    # dac_image = cv2.medianBlur(dac_image.astype('uint16'),5)
    # dac_image = cv2.GaussianBlur(dac_image,(sigma_g,sigma_g),0)/cv2.GaussianBlur(dac_mask,(sigma_g,sigma_g),0)
    # dac_image = dac_image[:,:,0]/dac_image[:,:,1]
    return dac_image.astype('uint16')
```

# Define functions to align, merge and cut the pointclouds¶

In [6]:

```
plt.close('all')

#%% also set up the cylinder filtering!
c_cylinder = geometry['c_cylinder']
r_cylinder = geometry['r_cylinder']
floor_point = geometry['floor_point']
floor_normal = geometry['floor_normal']
M0 = geometry['M0']

def apply_rigid_transformation(positions,R,t):
    # takes postions as a Nx3 vector and applies rigid transformation
    # make matrices
    A = np.asmatrix(positions)
    R = np.asmatrix(R)
    t = np.asmatrix(t).T

    # Matrix way:
    n = A.shape[0]
    A2 = np.matmul(R,A.T) + np.tile(t, (1, n))

    # print(str(i)+' after transform: '+str(A2.shape))
    # make it an array?
    return np.asarray(A2.T)


def cut_by_floor_roof(positions,floor_point,floor_normal,floor_cut=0.005,roof_cut=0.01):
    """
    Function to cut away the floor w/o a need to rotate the points fikst, just use the dot product trick
    # cut away floor?
    # use the equation of the plane: http://tutorial.math.lamar.edu/Classes/CalcIII/EqnsOfPlanes.aspx
    # and evaluate this to check if it's above or below: https://stackoverflow.com/questions/15688232/check-which-side-of-a-plane-points-are-on

    """
    # find the first coefficients of the equation of the plane!
    plane_coeffs = floor_normal

        # find a point above the plane!
    hover_point = floor_point + floor_normal * floor_cut
    roof_point = floor_point + floor_normal * roof_cut
        # calculate d, which is the dot product between a point on the plane and the normal
    floor_d = np.dot(floor_normal,hover_point)
    roof_d = np.dot(floor_normal,roof_point)

    # the idea is to calc ax+by+cz+d where abc is the normal and xyz is the point being tested
    # now do the dot product as the logic to pflip on the sign (don't care about equal to)
    #test_prod = np.dot(positions,plane_coeffs[0:3])
    # einsum is faster!
    test_prod = np.einsum('j,ij->i',plane_coeffs,positions)


    above_logic = (test_prod > floor_d) * (test_prod < roof_d)
    return above_logic


def align_by_floor(positions,floor_point,M0):
    positions = positions - floor_point
    # rotate!
    #TODO desperate need to convert everything to 4D transformations!! Here translation is first, then rotate. Above it's the other way around Yikes!!
    positions = np.transpose(np.matmul(M0,positions.T))

    # cut_logic = (positions[:,2] > 0.01 ) * (positions[:,2] < 0.1 )
    return positions

def cut_by_cylinder(positions,r_factor= .99 ,showplot = False):
    dd = np.sqrt( (positions[:,0] - c_cylinder[0])**2 + (positions[:,1] - c_cylinder[1])**2 )

    logic = dd < r_factor*r_cylinder

    if showplot:

        # easy3d(positions[::10,:])
        positions = positions[logic,:]
        easy3d(positions[:,:])

        plt.figure()
        plt.hist(dd)
        plt.show()

    return logic
```

# A few simple plotting functions¶

In [7]:

```
# For plotting just pointcloud data
def cheap3d(positions,rgb = None, new=True):
    from matplotlib import rcParams
    rcParams['font.family'] = 'serif'
    #   3D plot of the
    import matplotlib.pyplot as plt
    from mpl_toolkits.mplot3d import Axes3D

    X, Y, Z = positions[:,0],positions[:,1],positions[:,2]

    #   3D plot of Sphere
    if new:
        fig = plt.figure()
        ax = fig.add_subplot(111, projection='3d')
    else:
        ax = plt.gca()
        ax = ax.add_subplot(111, projection='3d')


    if rgb is None:
        ax.scatter(X, Y, Z, zdir='z', s=10, c='b',rasterized=True)
    else:
        ax.scatter(X, Y, Z, zdir='z', s=6, c=rgb/255,rasterized=True)
#     ax.set_aspect('equal')
    #ax.set_xlim3d(-35, 35)
    #ax.set_ylim3d(-35,35)
    #ax.set_zlim3d(-70,0)
    ax.set_xlabel('$x$ (mm)',fontsize=16)
    ax.set_ylabel('\n$y$ (mm)',fontsize=16)
    zlabel = ax.set_zlabel('\n$z$ (mm)',fontsize=16)

    max_range = np.array([X.max()-X.min(), Y.max()-Y.min(), Z.max()-Z.min()]).max() / 2.0

    mid_x = (X.max()+X.min()) * 0.5
    mid_y = (Y.max()+Y.min()) * 0.5
    mid_z = (Z.max()+Z.min()) * 0.5
    ax.set_xlim(mid_x - max_range, mid_x + max_range)
    ax.set_ylim(mid_y - max_range, mid_y + max_range)
    ax.set_zlim(mid_z - max_range, mid_z + max_range)

    plt.show()
```

In [8]:

```
# For plotting pointcloud data with keypoints
def cheap4d(pos,keyp,keyp_idx,rgb = None, new=True):
    from matplotlib import rcParams
    rcParams['font.family'] = 'serif'
    #   3D plot of the
    import matplotlib.pyplot as plt
    from mpl_toolkits.mplot3d import Axes3D

    X, Y, Z = pos[:,0],pos[:,1],pos[:,2]

    #   3D plot of Sphere
    if new:
        fig = plt.figure()
        ax = fig.add_subplot(111, projection='3d')
    else:
        ax = plt.gca()
        ax = ax.add_subplot(111, projection='3d')


    if rgb is None:
        ax.scatter(X, Y, Z, zdir='z', s=10, c='k', alpha = .1,rasterized=True)
    else:
        ax.scatter(X, Y, Z, zdir='z', s=6, c=rgb/255,alpha = .5,rasterized=True)
#     ax.set_aspect('equal')
    #ax.set_xlim3d(-35, 35)
    #ax.set_ylim3d(-35,35)
    #ax.set_zlim3d(-70,0)
    
    body_colors = ['dodgerblue','red','lime','orange']
    for i,body in enumerate(keyp_idx):
        ax.scatter(keyp[i,0], keyp[i,1], keyp[i,2], zdir='z', s=100, c=body_colors[body],rasterized=True)
    
    ax.set_xlabel('$x$ (mm)',fontsize=16)
    ax.set_ylabel('\n$y$ (mm)',fontsize=16)
    zlabel = ax.set_zlabel('\n$z$ (mm)',fontsize=16)

    max_range = np.array([X.max()-X.min(), Y.max()-Y.min(), Z.max()-Z.min()]).max() / 2.0

    mid_x = (X.max()+X.min()) * 0.5
    mid_y = (Y.max()+Y.min()) * 0.5
    mid_z = (Z.max()+Z.min()) * 0.5
    ax.set_xlim(mid_x - max_range, mid_x + max_range)
    ax.set_ylim(mid_y - max_range, mid_y + max_range)
    ax.set_zlim(mid_z - max_range, mid_z + max_range)

    plt.show()
plt.close('all')
# cheap4d(pos[::9],keyp,keyp_idx)
```

# Wrappers to load pairs of depth and color images, load keypoints coordinates¶

In [9]:

```
def load_dc_frames(frame,dev,d_files,png_files):
    # get the frame # from the master table
    # load the frame and the keypoints
    d_image = np.load(d_files[dev][frame])
#     c_image = cv2.imread(png_files[dev][frame])
    c_image = np.zeros((240,320,3),dtype='uint8')
    c_image[30:,:,:] = cv2.imread(png_files[dev][frame])
    return c_image,d_image

# make a figure to 'unpack the integers'
def unpack_keypoints(keyp_datasets,dev,raw_frame):
    # some unpack index wrangling
    raw_line = keyp_datasets[dev][raw_frame]
    fourth_length = int(len(raw_line)/4)
    xy = raw_line[:(2*fourth_length)].reshape((-1,2))
    pxy = raw_line[(2*fourth_length):-fourth_length]/100.
    score_idx = raw_line[-fourth_length:]
    return xy, pxy, score_idx
```

In [10]:

```
# function which loads a single frame with keypoints
dev = 0
frame = 1000
def load_d_and_keyp(frame,dev):
    # get the frame # from the master table
    frame = timing['master_frame_table'][frame,dev]
    # # load the frame and the keypoints
    # d_image = np.load(d_files[dev][raw_frame])
    c_image,d_image = load_dc_frames(frame,dev,d_files,png_files)
    dac_image =  align_d_to_c(d_image,c_image,dev,geometry)
    xy, pxy, score_idx = unpack_keypoints(keyp_datasets,dev,frame)

    # scale the keypoints up to the color space, remember the 32 offset
    # make the resolution correct, i.e. set the height to 192
    # ALSO remember that there was a 30 px cut
    pad_top = 8
    pad_bottom = 10
    # im = im[pad_top:-pad_bottom,:,:]
    xy_cij = 4 * xy + np.array([pad_top+30,0])

    # move the keypoints from color pixel space to depth pixel space
    # discussion here: https://github.com/IntelRealSense/librealsense/issues/2137

    #TODO take average around point!
    xy_d = dac_image[xy_cij[:,0],xy_cij[:,1]] 
    
    xy_d = np.zeros_like(xy[:,0])
    for i in range(xy.shape[0]):
        pixels = dac_image[np.meshgrid( np.arange(-2,3) + xy_cij[i,0], np.arange(-2,3) + xy_cij[i,1])]
        xy_d[i] = np.nanmax( [np.median(pixels[pixels > 0].ravel()) , 0])


    # convert the keypoints to XYZ
    fx,fy,ppx,ppy,depth_scale,fps,frame_width,frame_height = geometry['d_cam_params'][dev]
    fps,frame_width,frame_height = fps.astype('int'),frame_width.astype('int'),frame_height.astype('int')
    fx_c,fy_c,ppx_c,ppy_c,_,_,_,_ =  geometry['c_cam_params'][dev]

    # FIX the geometry due to downsample
    # divide the xy because downsample
    fx,fy,ppx,ppy = fx/2,fy/2,ppx/2,ppy/2
    fx_c,fy_c,ppx_c,ppy_c = fx_c/2,fy_c/2,ppx_c/2,ppy_c/2
    frame_width,frame_height = int(frame_width/2),int(frame_height/2)


    z_c = xy_d*depth_scale # +1e-6

    # and now use pinhole cam function to get the x and y
    x_c = (xy_cij[:,1] - ppx_c) * z_c / fx_c
    y_c = (xy_cij[:,0] - ppy_c) * z_c / fy_c    

    # # and pack to a stack of positions!
    keyp_color_space = np.vstack((x_c,y_c,z_c)).T    

    # SWING THESE POSITIONS TO THE DEPTH SPACE
    R_extr = geometry['R_extrinsics'][dev]
    t_extr = geometry['t_extrinsics'][dev]
    keyp_depth_space = np.einsum('ij,aj->ai',R_extr.T,(keyp_color_space - t_extr ))

    # also unpack the depth points!
    # get the expanded once more
    pi,pj = np.where( (d_image>0) )

    # pi,pj = np.where( (d>0 ) ) # simply all
    # get the depth of the masked pixels as a raveled list
    dij = d_image[pi,pj]

    # z is easy to calculate, it's just the depth
    z_m = dij*depth_scale # +1e-6
    # z_m = np.clip(z_m,0.,.5)

    # and now use pinhole cam function to get the x and y
    x_m = (pj - ppx) * z_m / fx
    y_m = (pi - ppy) * z_m / fy

    d_positions = np.vstack((x_m,y_m,z_m)).T    
    
    points_to_cam = np.linalg.norm(d_positions,axis=1)

    d_world = apply_rigid_transformation(d_positions,geometry['R_world'][dev],geometry['t_world'][dev])
    keyp_world = apply_rigid_transformation(keyp_depth_space,geometry['R_world'][dev],geometry['t_world'][dev])    
    
    # add the distance as a fourth dimension to the positions
    d_world_weights = np.hstack([d_world,points_to_cam[:,np.newaxis]])
    
    return d_world_weights,keyp_world,pxy,score_idx
```

# Plot a frame, to make sure everything looks good¶

In [15]:

```
# # plot the keypoints in depth space
# plt.figure()
# dummy = (d_image%255)/5
# dummy[xy_cij[:,0],xy_cij[:,1]] = 255
# plt.imshow(dummy)
# # plt.plot(xy_cij[:,0],xy_cij[:,1],'or')
if True:
    frame = 2000
    c_image,d_image = load_dc_frames(frame,dev,d_files,png_files)
    dac_image =  align_d_to_c(d_image,c_image,dev,geometry)
    xy, pxy, score_idx = unpack_keypoints(keyp_datasets,dev,frame)
    # scale the keypoints up to the color space, remember the 32 offset
    # make the resolution correct, i.e. set the height to 192
    # ALSO remember that there was a 30 px cut
    pad_top = 8
    pad_bottom = 10
    # im = im[pad_top:-pad_bottom,:,:]
    xy_cij = 4 * xy + np.array([pad_top+30,0])
    # plot the keypoints in depth space
    plt.figure(figsize = (10,6))
    plt.subplot(1,2,1)
    dummy = (np.mean(c_image,2))/2
    dummy[xy_cij[:,0],xy_cij[:,1]] = 255
    plt.imshow(dummy)
    plt.title('Color image, high value pixels are keypoints')
    # plt.plot(xy_cij[:,0],xy_cij[:,1],'or')

    plt.subplot(1,2,2)
    dummy = (dac_image%255)/(2)
    dummy[xy_cij[:,0],xy_cij[:,1]] = 255
    plt.imshow(dummy)
    plt.title('Depth aligned to color image, high value pixels are keypoints')
    plt.show()
```

# Make a wrapper for loading and composing a full 3D frame¶

In [17]:

```
# load the point cloud, the keypoints fof a frame!

def load_d_and_keyp_all(frame):
    d_world_list = [None]*4
    keyp_list = [None]*4 
    pkeyp_list = [None]*4 
    score_idx_list = [None]*4
    for dev in range(4):
        d_world,keyp_world,pkeyp,score_idx = load_d_and_keyp(frame,dev)
        d_world_list[dev] = d_world
        keyp_list[dev] = keyp_world
        score_idx_list[dev] = score_idx
        pkeyp_list[dev] = pkeyp
        
    return np.concatenate(d_world_list), np.concatenate(keyp_list), np.concatenate(pkeyp_list), np.concatenate(score_idx_list)

def load_full_frame(frame):
    pos, keyp, pkeyp, keyp_idx = load_d_and_keyp_all(frame)
    # split out, 
    pos, points_to_cam = pos[:,:3],pos[:,3]
    # TODO could clean this up, sort of silly right now
    
    cut_logic = cut_by_floor_roof(pos,floor_point,floor_normal,floor_cut=0.006,roof_cut=0.15)
    
    pos = align_by_floor(pos,floor_point,M0)
    keyp = align_by_floor(keyp,floor_point,M0)

    cyl_logic = cut_by_cylinder(pos,r_factor= .97,showplot = False)
    
    # select points above the floor and inside the cylinder
    selection_logic = cyl_logic*cut_logic
    pos = pos[selection_logic,:]

#     pos = pos[cyl_logic]
    
    keyp_logic = (pkeyp > .06) * cut_by_cylinder(keyp,r_factor= .99,showplot = False)
    
    # and the weigths as well!
    pos_weights = points_to_cam[selection_logic]**2

    # TODO!!!! CENTER EVERYTHING BY THE CENTER OF THE CYLINDER
    
    pos[:,:2] = pos[:,:2] - c_cylinder[np.newaxis,:]
    keyp[:,:2] = keyp[:,:2] - c_cylinder[np.newaxis,:]
    
    return pos, pos_weights, keyp[keyp_logic,:], pkeyp[keyp_logic], keyp_idx[keyp_logic]
```

# Again, check with a few plots that it looks fine¶

In [18]:

```
frame = 15000+1
pos, pos_weights, keyp, pkeyp, keyp_idx = load_full_frame(frame)
cheap4d(pos[::3],keyp,keyp_idx)
```

```
/home/chrelli/anaconda2/envs/mousepose/lib/python3.6/site-packages/ipykernel_launcher.py:29: FutureWarning: Using a non-tuple sequence for multidimensional indexing is deprecated; use `arr[tuple(seq)]` instead of `arr[seq]`. In the future this will be interpreted as an array index, `arr[np.array(seq)]`, which will result either in an error or a different result.
/home/chrelli/anaconda2/envs/mousepose/lib/python3.6/site-packages/numpy/core/fromnumeric.py:3118: RuntimeWarning: Mean of empty slice.
  out=out, **kwargs)
/home/chrelli/anaconda2/envs/mousepose/lib/python3.6/site-packages/numpy/core/_methods.py:85: RuntimeWarning: invalid value encountered in double_scalars
  ret = ret.dtype.type(ret / rcount)
```

In [20]:

```
cheap3d(pos[::3],rgb = pos_weights[::3]/(pos_weights.max()), new=True)
```

In [21]:

```
plt.close('all')
```

# Make functions to pack and unpack 3D data format¶

In [22]:

```
# make functions to pack and unpack data to a single string
def pack_to_jagged(pos, pos_weights, keyp, pkeyp, keyp_idx):
    ''' Takes the NX3, N, Mx3, M, M shapes and packs to a single float16
    We ravel the position, ravel the keyp, stack everything and 
    - importantly - we also save M, the number of keypoints'''
    n_keyp = keyp_idx.shape[0]
    block = np.hstack([pos,pos_weights[:,np.newaxis]])
    jagged_line = np.hstack((block.ravel(),keyp.ravel(),pkeyp,keyp_idx,n_keyp))
    return jagged_line

jagged_line = pack_to_jagged(pos, pos_weights, keyp, pkeyp, keyp_idx)
```

In [23]:

```
def unpack_from_jagged(jagged_line):
    ''' THE REVESER SO HERE IT UNPACKS AGAIN SO THE DATA CAN BE SAVED
    AS A JAGGED H5PY DATASET 
    FROM OTHER: Takes the NX3, N, Mx3, M, M shapes and packs to a single float16
    We ravel the position, ravel the keyp, stack everything and 
    - importantly - we also save M, the number of keypoints'''
    n_keyp = int(jagged_line[-1])
    keyp_idx2 = jagged_line[-(1+n_keyp):-1].astype('int')
    pkeyp2 = jagged_line[-(1+2*n_keyp):-(1+n_keyp)]
    keyp2 = jagged_line[-(1+5*n_keyp):-(1+2*n_keyp)].reshape((n_keyp,3))
    block2 = jagged_line[:-(1+5*n_keyp)].reshape((-1,4))
    pos2,pos_weights2 = block2[:,:3], block2[:,3]
    return pos2, pos_weights2, keyp2, pkeyp2, keyp_idx2

pos2, pos_weights2, keyp2, pkeyp2, keyp_idx2 = unpack_from_jagged(jagged_line)
```

# Process all frames and dump to hdf5 file as jagged arrays¶

In [ ]:

```
# NOW we actually pre-process the data, required for saving and compressing the data

# OK OK OK, now select the reference camera!
n_frames = len(timing['reference_stamps'])

# open a file for the 
with h5py.File(top_folder_0+'/pre_processed_frames.hdf5', mode='w') as hdf5_file:
    # make the variable length dataset, go with float16 for now
    dt = h5py.special_dtype(vlen=np.dtype('float16'))
    hdf5_file.create_dataset('dataset', (n_frames,), dtype=dt)
    
    # now, loop over the frames and save them
    for frame in tqdm_notebook(range(n_frames)):
        # load the frame
        pos, pos_weights, keyp, pkeyp, keyp_idx = load_full_frame(frame)
        # pack to a single line for jagged h5py
        jagged_line = pack_to_jagged(pos[::2], pos_weights[::2], keyp, pkeyp, keyp_idx)
        # write to the h5py file
        hdf5_file['dataset'][frame] = jagged_line.astype('float16')
    print("Done with {} frames!".format(n_frames))
```

# Check if file is good: Reload data from hdf5 file, unpack and plot¶

In [25]:

```
# now, try to load some data, to see if it is OK
with h5py.File(top_folder_0+'/pre_processed_frames.hdf5', mode='r') as hdf5_file:
    print(hdf5_file.keys())
    print(len(hdf5_file['dataset']))
    jagged_line = hdf5_file['dataset'][71950] 
    
    pos, pos_weights, keyp, pkeyp, keyp_idx = unpack_from_jagged(jagged_line)
    print(keyp_idx)
    cheap4d(pos,keyp,keyp_idx)
```

```
<KeysViewHDF5 ['dataset']>
74962
[0 1 1 2 2 3 0 1 1 1 2 3 0 1 1 1 3 3 0 1 1 1 2 3]
```

# Bonus: Make a plot to show merging of four clouds, for the manuscript¶

In [ ]:

```
import matplotlib

# Say, "the default sans-serif font is COMIC SANS"
matplotlib.rcParams['font.sans-serif'] = "Liberation Sans"
# Then, "ALWAYS use sans-serif fonts"
matplotlib.rcParams['font.family'] = "sans-serif"

matplotlib.rc('font', family='sans-serif') 
matplotlib.rc('text', usetex='false') 
matplotlib.rcParams.update({'font.size': 15})

from palettable.cmocean.sequential import Algae_6
cmpl=Algae_6.mpl_colors


frame = 12000+100-1000
elev,azim = 40,-104
elev,azim = 28,-119


pos, pos_weights, cam_idx, keyp, pkeyp, keyp_idx = load_full_frame(frame)

# def cheap3d(positions,rgb = None, new=True):
import matplotlib.pyplot as plt
from mpl_toolkits.mplot3d import Axes3D

positions = pos

X, Y, Z = positions[:,0],positions[:,1],positions[:,2]

#   3D plot of Sphere
fig = plt.figure(figsize = (7,7))
ax = fig.add_subplot(111, projection='3d')

for dev in range(4):
    logi = cam_idx == dev
    ax.scatter(X[logi], Y[logi], Z[logi], zdir='z',c=cmpl[dev+1], s=1, rasterized=False)

max_range = np.array([X.max()-X.min(), Y.max()-Y.min(), Z.max()-Z.min()]).max() / 2.0

mid_x = (X.max()+X.min()) * 0.5
mid_y = (Y.max()+Y.min()) * 0.5
mid_z = (Z.max()+Z.min()) * 0.5
ax.set_xlim(mid_x - max_range, mid_x + max_range)
ax.set_ylim(mid_y - max_range, mid_y + max_range)
# ax.set_zlim(mid_z - max_range, mid_z + max_range)
ax.set_zlim(0, 2* max_range)

ax.set_xticklabels([])
ax.set_yticklabels([])
ax.set_zticklabels([])

ax.view_init(elev,azim)

plt.show()
```
