# Supplementary material for: Automatic mapping of multiplexed social receptive fields by deep learning and GPU-accelerated 3D videography
Source: Nat Commun. 2022 Feb 1;13:593. doi: 10.1038/s41467-022-28153-7 (PMC8807631; doi:10.1038/s41467-022-28153-7)
Supplement: Supplementary file 9 — Supplementary Software [file 41467_2022_28153_MOESM9_ESM.zip › ebbesen_froemke_2021_code/analysis/001_Generate_a_training_set.html]

001\_Generate\_a\_training\_set


In [1]:

```
import numpy as np
import cv2
import h5py
import matplotlib.pyplot as plt
import sys
import tqdm
import glob
import itertools

from os.path import expanduser
home = expanduser("~")
```

# Point to the recording folders, load the png files¶

In [2]:

```
# load the geometry
top_folder_0 = '/media/chrelli/Data0/recording_20190905-115115'
top_folder_1 = '/media/chrelli/Data1/recording_20190905-115115'
scene_folders = [top_folder_0,top_folder_0,top_folder_1,top_folder_1]
import pickle
geometry = pickle.load( open( scene_folders[0]+'/geometry.pkl', "rb" ) ) 
timing = pickle.load( open( scene_folders[0]+'/timing.pkl', "rb" ) )
print(geometry.keys())
print(timing.keys())
```

```
dict_keys(['start_frame', 'end_frame', 'd_cam_params', 'c_cam_params', 'R_extrinsics', 't_extrinsics', 'R_world', 't_world', 'M0', 'floor_point', 'floor_normal', 'c_cylinder', 'r_cylinder'])
dict_keys(['master_frame_table', 'reference_time_cam', 'reference_stamps', 'time_stamps', 'shifted_stamps'])
```

In [3]:

```
# use glob to make a list of the png files from all cameras
png_files = [glob.glob(scene_folders[i] + '/npy_raw/dev' +str(i) +'_cad_*.png') for i in range(4)]
png_files = [sorted(f) for f in png_files]
```

In [4]:

```
# Look at an example image
cam = 3
pic = cv2.imread(png_files[cam][230])
plt.imshow(pic[:,:,[2,1,0]])
```

Out[4]:

```
<matplotlib.image.AxesImage at 0x7f694b9b3518>
```

# Pick 400 random images¶

In [5]:

```
# make a training stack!
n_train = 400
frame_height,frame_width = 210,320
# n x w x h x cam
sampled_c_frames = np.empty((n_train,frame_height,frame_width,3,4)).astype('uint8')

for cam in range(4):
    for i,frame in enumerate( np.random.choice(len(png_files[cam]),n_train) ):
        c_image = cv2.imread(png_files[cam][frame])
        sampled_c_frames[i,:,:,:,cam] = c_image
        
# and stack the four camera views together
sampled_c_frames = np.concatenate( [sampled_c_frames[:,:,:,:,i] for i in range(4)] ,axis = 0)
```

In [6]:

```
sampled_c_frames.shape
```

Out[6]:

```
(1600, 210, 320, 3)
```

In [7]:

```
new_image = sampled_c_frames[500,...][:,:,[2,1,0]]
plt.imshow(new_image)
```

Out[7]:

```
<matplotlib.image.AxesImage at 0x7f694ba2e978>
```

In [8]:

```
new_image = sampled_c_frames[500,...][:,:,[2,1,0]]
# * Enter the alpha value [1.0-3.0]: 2.2
alpha = 1#.5
# * Enter the beta value [0-100]: 50
beta = 0
pic = cv2.convertScaleAbs(new_image, -1, alpha, beta)
plt.imshow(pic)
```

Out[8]:

```
<matplotlib.image.AxesImage at 0x7f694d220748>
```

In [9]:

```
# make a greyscale version
sampled_g_frames = sampled_c_frames @ [0.2989, 0.5870, 0.1140]
sampled_g_frames = sampled_g_frames.astype('uint16')
plt.imshow(sampled_g_frames[0,:,:])
sampled_g_frames.shape
```

Out[9]:

```
(1600, 210, 320)
```

# k-means to group similar images in clusters¶

In [10]:

```
# do k-means on the greyscale images!
from sklearn.cluster import MiniBatchKMeans
from sklearn.utils.validation import check_is_fitted

Machine = MiniBatchKMeans(n_clusters=12, init='k-means++', max_iter=1000,
                 batch_size=100, verbose=1, compute_labels=True,
                 random_state=None, tol=0.0, max_no_improvement=10,
                 init_size=None, n_init=10, reassignment_ratio=0.01)

# flatten everything, except the first dimension
g_frames_flat = np.reshape(sampled_g_frames,(sampled_g_frames.shape[0],-1))
# downsample here?
Machine.fit(g_frames_flat)
cluster_labels = Machine.predict(g_frames_flat)

# sampled_g_frames = sampled_g_frames.astype('uint8')
# plt.imshow(sampled_g_frames[0,:,:]
```

```
Init 1/10 with method: k-means++
Inertia for init 1/10: 12337371534.952845
Init 2/10 with method: k-means++
Inertia for init 2/10: 13381913977.132999
Init 3/10 with method: k-means++
Inertia for init 3/10: 12975223243.285252
Init 4/10 with method: k-means++
Inertia for init 4/10: 12674940295.282211
Init 5/10 with method: k-means++
Inertia for init 5/10: 12962862515.711441
Init 6/10 with method: k-means++
Inertia for init 6/10: 12957061912.840343
Init 7/10 with method: k-means++
Inertia for init 7/10: 12858661333.364788
Init 8/10 with method: k-means++
Inertia for init 8/10: 12937062676.498627
Init 9/10 with method: k-means++
Inertia for init 9/10: 13058941326.109694
Init 10/10 with method: k-means++
Inertia for init 10/10: 12707007331.146833
Minibatch iteration 1/16000: mean batch inertia: 49148655.934680, ewa inertia: 49148655.934680 
Minibatch iteration 2/16000: mean batch inertia: 48906888.024173, ewa inertia: 49118453.822187 
Minibatch iteration 3/16000: mean batch inertia: 49656860.135769, ewa inertia: 49185712.574664 
Minibatch iteration 4/16000: mean batch inertia: 48789747.952839, ewa inertia: 49136247.912350 
Minibatch iteration 5/16000: mean batch inertia: 48144500.018156, ewa inertia: 49012356.857485 
Minibatch iteration 6/16000: mean batch inertia: 42925169.602106, ewa inertia: 48251933.715027 
Minibatch iteration 7/16000: mean batch inertia: 42444863.905523, ewa inertia: 47526503.382797 
Minibatch iteration 8/16000: mean batch inertia: 52512330.670008, ewa inertia: 48149342.519238 
Minibatch iteration 9/16000: mean batch inertia: 46823012.995736, ewa inertia: 47983654.883572 
Minibatch iteration 10/16000: mean batch inertia: 48255856.821016, ewa inertia: 48017658.873259 
Minibatch iteration 11/16000: mean batch inertia: 41687400.143915, ewa inertia: 47226870.774653 
Minibatch iteration 12/16000: mean batch inertia: 40665979.270004, ewa inertia: 46407271.586065 
Minibatch iteration 13/16000: mean batch inertia: 46771379.435784, ewa inertia: 46452756.639121 
Minibatch iteration 14/16000: mean batch inertia: 44752480.256488, ewa inertia: 46240354.842415 
Minibatch iteration 15/16000: mean batch inertia: 44643447.832542, ewa inertia: 46040866.146616 
Minibatch iteration 16/16000: mean batch inertia: 42812057.662281, ewa inertia: 45637517.179179 
Minibatch iteration 17/16000: mean batch inertia: 44802555.367788, ewa inertia: 45533212.143402 
Minibatch iteration 18/16000: mean batch inertia: 40724862.123553, ewa inertia: 44932543.808630 
Minibatch iteration 19/16000: mean batch inertia: 46741269.819744, ewa inertia: 45158493.341561 
Minibatch iteration 20/16000: mean batch inertia: 44439428.587160, ewa inertia: 45068666.389107 
Minibatch iteration 21/16000: mean batch inertia: 50620149.601950, ewa inertia: 45762168.351985 
Minibatch iteration 22/16000: mean batch inertia: 45151331.340282, ewa inertia: 45685861.417356 
Minibatch iteration 23/16000: mean batch inertia: 41567838.342771, ewa inertia: 45171430.052636 
Minibatch iteration 24/16000: mean batch inertia: 37134007.630925, ewa inertia: 44167379.781342 
Minibatch iteration 25/16000: mean batch inertia: 42968258.685601, ewa inertia: 44017583.267196 
Minibatch iteration 26/16000: mean batch inertia: 43385569.820199, ewa inertia: 43938630.931531 
Minibatch iteration 27/16000: mean batch inertia: 44447646.534512, ewa inertia: 44002218.139898 
Minibatch iteration 28/16000: mean batch inertia: 47591026.351413, ewa inertia: 44450538.965821 
Minibatch iteration 29/16000: mean batch inertia: 45471574.380639, ewa inertia: 44578088.674106 
Minibatch iteration 30/16000: mean batch inertia: 48422137.717950, ewa inertia: 45058294.675835 
Minibatch iteration 31/16000: mean batch inertia: 43219549.161500, ewa inertia: 44828595.048810 
Minibatch iteration 32/16000: mean batch inertia: 43899563.572187, ewa inertia: 44712538.649482 
Minibatch iteration 33/16000: mean batch inertia: 41573947.792750, ewa inertia: 44320459.841645 
Minibatch iteration 34/16000: mean batch inertia: 42642893.010134, ewa inertia: 44110894.965754 
Minibatch iteration 35/16000: mean batch inertia: 42696429.517834, ewa inertia: 43934197.220855 
Minibatch iteration 36/16000: mean batch inertia: 45312063.853161, ewa inertia: 44106322.971299 
Minibatch iteration 37/16000: mean batch inertia: 41207619.364337, ewa inertia: 43744211.340198 
Minibatch iteration 38/16000: mean batch inertia: 44344549.852180, ewa inertia: 43819206.782045 
Minibatch iteration 39/16000: mean batch inertia: 47233834.827905, ewa inertia: 44245768.686587 
Minibatch iteration 40/16000: mean batch inertia: 38174718.749806, ewa inertia: 43487361.449013 
Minibatch iteration 41/16000: mean batch inertia: 46205121.549829, ewa inertia: 43826869.269227 
Minibatch iteration 42/16000: mean batch inertia: 47646257.904668, ewa inertia: 44303994.645297 
Minibatch iteration 43/16000: mean batch inertia: 47233785.445138, ewa inertia: 44669989.748338 
Minibatch iteration 44/16000: mean batch inertia: 40451338.172307, ewa inertia: 44142987.677628 
Minibatch iteration 45/16000: mean batch inertia: 40036568.098964, ewa inertia: 43630005.843941 
Minibatch iteration 46/16000: mean batch inertia: 45666165.853484, ewa inertia: 43884366.869493 
Minibatch iteration 47/16000: mean batch inertia: 42490193.006273, ewa inertia: 43710203.988391 
Minibatch iteration 48/16000: mean batch inertia: 44178701.788571, ewa inertia: 43768729.634885 
Minibatch iteration 49/16000: mean batch inertia: 46688635.137601, ewa inertia: 44133489.847591 
Minibatch iteration 50/16000: mean batch inertia: 40034367.425495, ewa inertia: 43621419.588741 
Converged (lack of improvement in inertia) at iteration 50/16000
Computing label assignment and total inertia
Computing label assignment and total inertia
```

In [11]:

```
# show the images in the clusters
plt.hist(cluster_labels)
np.unique(cluster_labels)
```

Out[11]:

```
array([ 0,  1,  2,  3,  4,  5,  6,  7,  8,  9, 10, 11], dtype=int32)
```

# Sample from the clusters and save as hdf5 file¶

In [12]:

```
# save these random frames and cluster identity as a dataset
with h5py.File(top_folder_0 + '/1600_random_frames.h5', mode='w') as h5file:
    h5file.create_dataset('c_images', shape=sampled_c_frames.shape, dtype=np.uint8, data=sampled_c_frames[:,:,:,[2,1,0]])
    h5file.create_dataset('cluster_labels', shape=cluster_labels.shape, dtype=np.uint8, data=cluster_labels)
```

In [13]:

```
count,_ = np.histogram(cluster_labels,bins=np.arange(12+1))
count 
n_pr_cluster = np.min([ 60,  np.min(count) ])
print(n_pr_cluster)

reference_index = []

for i in range(12):
    in_cluster = cluster_labels == i
    reference_index.append(np.arange(sampled_c_frames.shape[0])[in_cluster][:n_pr_cluster])

reference_index = np.hstack([i for i in reference_index])
n_train = len(reference_index)
n_train
```

```
48
```

Out[13]:

```
576
```

In [14]:

```
#save the training data!

n_train = len(reference_index)
with h5py.File(top_folder_0 + '/mouse_training_set.h5', mode='w') as h5file:
    c_dump = sampled_c_frames[reference_index,:,:,:]
    h5file.create_dataset('c_images', shape=c_dump.shape, dtype=np.uint8, data=c_dump[:,:,:,:])
    h5file.create_dataset('reference_index', shape=reference_index.shape, dtype=np.uint16, data=reference_index)
```

# Check the training set¶

In [15]:

```
# Make sure the images are in the file
for _ in range(6):
    with h5py.File(top_folder_0 + '/mouse_training_set.h5', mode='r') as h5file:
        ji = np.random.choice(np.arange(n_train))
        h5_c_image = h5file['c_images'][ji]

    plt.figure(figsize=(15,15))
    plt.imshow(h5_c_image[:,:,[2,1,0]])
    plt.show()

    h5file.close()
```
