# Supplementary material for: Automatic mapping of multiplexed social receptive fields by deep learning and GPU-accelerated 3D videography
Source: Nat Commun. 2022 Feb 1;13:593. doi: 10.1038/s41467-022-28153-7 (PMC8807631; doi:10.1038/s41467-022-28153-7)
Supplement: Supplementary file 9 — Supplementary Software [file 41467_2022_28153_MOESM9_ESM.zip › ebbesen_froemke_2021_code/analysis/007_Tracking_engine.html]

007\_Tracking\_engine


In [1]:

```
from IPython import get_ipython

%matplotlib qt
%load_ext autoreload
%autoreload 2


import time, os, sys, shutil
from utils.fitting_utils import *

# for math and plotting
import pandas as pd
import numpy as np
import scipy as sp
import matplotlib.pyplot as plt

import matplotlib
matplotlib.use('Qt5Agg')
# %matplotlib notebook
# %matplotlib widget
# %matplotlib qt5
# %matplotlib nbagg

from itertools import compress # for list selection with logical
from tqdm import tqdm

from multiprocessing import Process

# ALLSO JIT STUFF
from numba import jit, njit

# and pytorch
import torch

import sys, os, pickle
# import cv2
from colour import Color
import h5py
from tqdm import tqdm, tqdm_notebook
import glob
import itertools
```

In [2]:

```
# Check CUDA
print(torch.cuda.is_available())
print(torch.cuda.device_count())
print(torch.cuda.get_device_name(0))
torch_device = torch.device("cuda:0" if torch.cuda.is_available() else "cpu")
print(torch_device)
```

```
True
1
GeForce RTX 2080 Ti
cuda:0
```

In [3]:

```
# setup for pretty plotting
matplotlib.rcParams['font.sans-serif'] = "Liberation Sans"
# Then, "ALWAYS use sans-serif fonts"
matplotlib.rcParams['font.family'] = "sans-serif"

matplotlib.rc('font', family='sans-serif') 
matplotlib.rc('text', usetex='false') 
matplotlib.rcParams.update({'font.size': 13})

from palettable.cmocean.sequential import Algae_6
cmpl = Algae_6.mpl_colors

def adjust_spines(ax, spines):
    for loc, spine in ax.spines.items():
        if loc in spines:
            spine.set_position(('outward', 10))  # outward by 10 points
            spine.set_smart_bounds(True)
        else:
            spine.set_color('none')  # don't draw spine

    # turn off ticks where there is no spine
    if 'left' in spines:
        ax.yaxis.set_ticks_position('left')
    else:
        # no yaxis ticks
        ax.yaxis.set_ticks([])

    if 'bottom' in spines:
        ax.xaxis.set_ticks_position('bottom')
    else:
        # no xaxis ticks
        ax.xaxis.set_ticks([])
```

# Load the pre-processed data and display an example frame¶

In [4]:

```
data_folder = 'example_data/tracking/'

# load ALL the frames as jagged lines 
with h5py.File(data_folder+'/pre_processed_frames.hdf5', mode='r') as hdf5_file:
    print(hdf5_file.keys())
    print(len(hdf5_file['dataset']))
    jagged_lines = hdf5_file['dataset'][...]
```

```
<KeysViewHDF5 ['dataset']>
74962
```

In [5]:

```
from utils.cuda_tracking_utils import unpack_from_jagged, cheap4d
# kill first 6 secs of the frames (delay is ~180)
start_frame = 30*60
pos, pos_weights, keyp, pkeyp, ikeyp = unpack_from_jagged(jagged_lines[start_frame])
print(ikeyp)
print(pos.shape)
fig = plt.gcf()
plt.title("N positions is {}".format(pos.shape))
plt.show()
cheap4d(pos,keyp,ikeyp)
```

```
[0 1 1 2 3 0 1 1 1 2 2 0 2 0 1 1 1 2 3]
(2986, 3)
```

# Initialize tracking with a click¶

In [6]:

```
from utils.cuda_tracking_utils import body_constants, particles_to_distance_cuda, clean_keyp_by_r
from utils.cuda_tracking_utils import loading_wrapper
from utils.clicking import *

click_start = True
if True:
    pos,pos_weights,keyp,ikeyp = loading_wrapper(start_frame,jagged_lines)
    x0_start,click_holder = initialize_x0(pos.cpu().numpy(),click_start=click_start)

x0_start = x0_start[[1,2,3,4,5,6,7,8,9,11,12,13,15,16,17,18,19]]
    
part = torch.Tensor(x0_start).to(torch_device).unsqueeze(0)
# no need for the the particle to have gradients
part.requires_grad = False
keyp,ikeyp = clean_keyp_by_r(part,keyp,ikeyp)

print(part)
print(part.shape)
print(pos.shape)
```

```
/home/chrelli/git/3d_sandbox/share_code/analysis/utils/clicking.py:105: MatplotlibDeprecationWarning: Adding an axes using the same arguments as a previous axes currently reuses the earlier instance.  In a future version, a new instance will always be created and returned.  Meanwhile, this warning can be suppressed, and the future behavior ensured, by passing a unique label to each axes instance.
  plt.axes().set_aspect('equal', 'datalim')
/home/chrelli/git/3d_sandbox/share_code/analysis/utils/clicking.py:131: MatplotlibDeprecationWarning: Adding an axes using the same arguments as a previous axes currently reuses the earlier instance.  In a future version, a new instance will always be created and returned.  Meanwhile, this warning can be suppressed, and the future behavior ensured, by passing a unique label to each axes instance.
  plt.axes().set_aspect('equal', 'datalim')
```

```
tensor([[ 0.0000,  1.3182,  0.8000,  0.0000,  0.0000, -0.2374,  0.0947, -0.1031,
          0.0220,  0.0000, -1.7430,  0.8000,  0.0000,  0.3141,  0.1382, -0.0813,
          0.0220]], device='cuda:0')
torch.Size([1, 17])
torch.Size([2986, 3])
```

# Plot the clicked start¶

In [7]:

```
plt.close('all')
positions = pos.cpu().numpy()
import cmocean
###############
# Show a 2D plot and ask for two clicks
###############
plt.figure(figsize = (4,4))
plt.scatter(positions[:,0],positions[:,1],c=positions[:,2]/np.max(positions[:,2]),s=5,cmap=cmocean.cm.algae_r)
ax = plt.gca
# plt.axes().set_aspect('equal', 'datalim')
# plt.title('click center of hip, then mid, then head of mouse!')
w,h = 570,800
# plt.get_current_fig_manager().window.setGeometry(1920-w-10,60,w,h)

clicks = np.vstack(click_holder)
plt.plot(clicks[:3,0],clicks[:3,1],'o-',c=cmpl[1],lw=2)
plt.plot(clicks[3:,0],clicks[3:,1],'o-',c=cmpl[1],lw=2)

plt.plot([.15,.2],[-.16,-.16],'-k')

# plt.xticks([])
# plt.yticks([])
plt.axis('equal')
plt.axis('off')
# plt.xlabel('x [m]')
# plt.ylabel('y [m]')
ax = plt.gca()
# adjust_spines(ax,['bottom','left'])
plt.tight_layout()

plt.show()
```

# Import the actual particle filter tracking engine, 'MousePFilt', and fit the first frame¶

In [8]:

```
# get the limits for the tracking and the residual functions
from utils.cuda_tracking_utils import search_cone, global_min, global_max
from utils.cuda_tracking_utils import add_implant_residual,add_body_residual,add_ass_residual, add_ear_residual, add_nose_residual
```

In [10]:

```
from utils.cuda_tracking_utils_weights_for_figures import MousePFilt, make_some_bounds,particles_to_body_supports_cuda

upper_bound,lower_bound = make_some_bounds(part,search_cone,global_max,global_min)
pzo = MousePFilt(swarm_size = 200)

pzo.search_space(upper_bound,lower_bound)

# populate the tracker
pzo.populate(sobol = True)

# send the data for tracking
pzo.pos = pos
pzo.pos_weights = pos_weights
pzo.keyp = keyp
pzo.ikeyp = ikeyp

pzo.max_iterations = 10
self = pzo
pzo.run2(cinema=False)
```

```
it 0 of 10, best loss is 0.0158627238124609, time 0.043894153997825924
it 1 of 10, best loss is 0.013409052044153214, time 0.03448206699977163
it 2 of 10, best loss is 0.012639489024877548, time 0.04041906099882908
it 3 of 10, best loss is 0.012183340266346931, time 0.017488825003965758
it 4 of 10, best loss is 0.01201407890766859, time 0.01733491699997103
it 5 of 10, best loss is 0.01195606030523777, time 0.01738783600012539
it 6 of 10, best loss is 0.011938204057514668, time 0.017349959998682607
it 7 of 10, best loss is 0.011921667493879795, time 0.017381060002662707
it 8 of 10, best loss is 0.011915399692952633, time 0.017358592995151412
it 9 of 10, best loss is 0.011910584755241871, time 0.017360957004711963
tensor([[   -inf,    -inf,  0.0000,    -inf, -1.0467,    -inf,    -inf,    -inf,
          0.0000,    -inf,    -inf,  0.0000, -1.0467,    -inf,    -inf,    -inf,
          0.0000]], device='cuda:0')
```

# You can plot without estimating a single fit to view filter behavior...¶

In [10]:

```
# like, after 1,5 and 10 iterations
plt.close('all')
pzo.plot_status(reduce_mean=False,keep_open=True,plot_ellipsoids=False)
```

# ...or make a point estimate and plot the ellipsoids¶

In [11]:

```
plt.close('all')
pzo.plot_status(reduce_mean=True,keep_open=True,plot_ellipsoids=True)
```

# Make a wrapper to run the particle filter across all frames, set options¶

In [13]:

```
pzo = MousePFilt(swarm_size = 200)

def pzo_wrapper(part,pos,pos_weights,keyp,ikeyp,pzo):

    upper_bound,lower_bound = make_some_bounds(part,search_cone,global_max,global_min)
    
    pzo.search_space(upper_bound,lower_bound)
    pzo.populate(sobol = True)

    pzo.pos = pos
    pzo.pos_weights = pos_weights

    pzo.keyp = keyp
    pzo.ikeyp = ikeyp

    pzo.max_iterations = 5

    pzo.run2(verbose=False,use_weights = False,barrier = True,fast_sort = True)
    
    return pzo.meanwinner
```

# Make a function to dump plots during tracking¶

In [14]:

```
plt.close('all')

from utils.plotting_during_tracking import *

def plot_single_frame(part,pos, keyp, ikeyp,frame):
    plt.ioff()
    plt.close('all')
    # the winning mouse is the one, with the lowest final loss
    #end_loss = [np.mean(ll[-1:]) for ll in ll_holder]
    dist0,_,body_support_0 = particles_to_distance_cuda(part[:,:9],pos,implant = True)
    dist1,_,body_support_1 = particles_to_distance_cuda(part[:,9:],pos,implant = False)
    body_supports = [body_support_0,body_support_1]
    
    #best_idx = np.argmin(end_loss)
    #best_mouse = best_holder[best_idx]

    fig = plt.figure(figsize=(7.5,7.5))

    ax = fig.add_subplot(1, 1, 1, projection='3d')
    plot_particles_new_nose(ax,part.cpu().numpy(),pos.cpu().numpy(),body_constants,alpha = .5,keyp = keyp.cpu(), ikeyp = ikeyp.cpu(),body_supports = [ [i.cpu() for i in j] for j in body_supports] )
    
    plt.axis('tight')
    
    ax.set_xlim(-.10,.20)
    ax.set_ylim(-.20,.1)
    ax.set_zlim(0,.3)
    

    ax.view_init(elev=60., azim=-147.)

    
    plt.savefig('frames/frame_'+str(frame).zfill(6)+'.png')
#     plt.show()
    
    plt.close('all')
    
# frame = start_frame
# plot_single_frame(part,pos, keyp, ikeyp,frame)
```

# And import a bank for online filtering and prediction¶

In [15]:

```
plt.close('all')

from utils.cuda_tracking_utils import rls_bank

def ML_predict(bank,i_frame,embedding,tracking_holder,guessing_holder):
    # # do the RLS step to predict the next step
    if (i_frame > embedding + 2)*True:
        x_train = np.flip( tracking_holder[:-1,(i_frame-embedding):i_frame],axis = 1)
        y_train = tracking_holder[:-1,i_frame]
        d = torch.from_numpy(y_train.copy())
        x = torch.from_numpy(x_train.copy())
        # make sure the type is right
        d = torch.tensor(d,dtype = torch.float32)
        x = torch.tensor(x,dtype = torch.float32)

        # and send to the holder
        bank.adapt(d,x)

        # guess the upcoming step!
        x_predict = torch.cat((d.unsqueeze(1),x[:,:-1]),1)
        
        part_guess = bank.predict(x_predict)
        if ( i_frame +1 ) < ( guessing_holder.shape[1] - 2 ):
            guessing_holder[:-1,i_frame+1] = part_guess[:].numpy()
        return bank,part_guess.unsqueeze(0),guessing_holder
    else:
        return bank,0.,guessing_holder
```

# Now, run the tracking across all frames and save to disk¶

In [ ]:

```
start_frame =  30*60-200

n_frames = len(jagged_lines)-1-start_frame

# do 1000 frames!
# n_frames = 10000
# do two mins for profiling
# n_frames = 2*60*60 

end_frame = start_frame + n_frames

### START ###
pos,pos_weights,keyp,ikeyp = loading_wrapper(start_frame,jagged_lines)
x0_start,clicked_points = initialize_x0(pos.cpu().numpy(),click_start=True)
x0_start = x0_start[[1,2,3,4,5,6,7,8,9,11,12,13,15,16,17,18,19]]
part = torch.Tensor(x0_start).to(torch_device).unsqueeze(0)

pzo = MousePFilt(swarm_size = 200)

part = pzo_wrapper(part,pos,pos_weights,keyp,ikeyp,pzo)


embedding = 5
bank = rls_bank(n_vars = part.shape[1], embedding=embedding)
bank.mu = .99

x0_trace = []
frame_trace = []
history_trace = []
# just make a numpy holder for it directly
# and a frame index which tells us which frame we're currently tracking
tracking_holder = np.zeros((part.shape[1]+1,n_frames))
guessing_holder = np.zeros((part.shape[1]+1,n_frames))*np.nan

from tqdm import tqdm

with torch.no_grad():
    for i_frame, this_frame in enumerate(tqdm_notebook(range(start_frame,start_frame+n_frames))):
        # if we've learned, preditc

        # load and fit
        pos,pos_weights,keyp,ikeyp = pos,pos_weights,keyp,ikeyp = loading_wrapper(this_frame,jagged_lines)
        pos = pos#[::4]

        keyp,ikeyp = clean_keyp_by_r(part,keyp,ikeyp)
    #     part,history = klm_routine(part,pos,keyp,ikeyp,max_iters = 100,verbose=False,save_history = True,ftol = 1e-4)
    #     part, histo = pzo_step(part,pos,keyp,ikeyp)
        part = pzo_wrapper(part,pos,pos_weights,keyp,ikeyp,pzo)
        # 3. add to fitting history
        x0_trace.append(part.cpu().numpy())
        frame_trace.append(this_frame)
    #     history_trace.append(history)
        # and update the frame index and the tracking_holder
        tracking_holder[:-1,i_frame] = part[0,:].cpu().numpy()
        tracking_holder[-1,i_frame] = this_frame

        # always adapt!
        if True:
            bank,part_guess,guessing_holder = ML_predict(bank,i_frame,embedding,tracking_holder,guessing_holder)

        if i_frame > 150 and True:
            # do prediction after the first 150 frames
            pass
    #         part_guess[:,[5,13]] = part[:,[5,13]]
    #         part = part_guess
    #         part[:,[0,1,2,6,7,8,9,10,11,14,15,16]] = part_guess[:,[0,1,2,6,7,8,9,10,11,14,15,16]]
            part[:,[6,7,8,14,15,16]] = part_guess[:,[6,7,8,14,15,16]].to(torch_device)

        if i_frame%2 == 0 and False:
            # fully update the 
            if i_frame > 150:
                plot_single_frame(part_guess.to(torch_device),pos, keyp, ikeyp,this_frame)    
            else:
                plot_single_frame(part,pos, keyp, ikeyp,this_frame)    

        if i_frame%6000 == 0:
            top_folder = 'frames/'
            print("saving tracking at frame {} of {}...".format(i_frame,start_frame+n_frames))
            np.save(top_folder+'tracking_holder.npy',tracking_holder)
            np.save(top_folder+'guessing_holder.npy',guessing_holder)
            np.save(top_folder+'body_constants.npy',body_constants)
            print("tracking saved!")    

            
# TODO also add the date of the folder as a string?
tracked_behavior = {
    "var": ['b','c','s','psi','theta','phi','x','y','z','b','c','s','theta','phi','x','y','z'],
    "ivar": ['b0','c0','s0','psi0','theta0','phi0','x0','y0','z0','b1','c1','s1','theta1','phi1','x1','y1','z1'],
    "body_constants": body_constants,
    "start_frame": start_frame,
    "end_frame": end_frame,
    "tracking_holder": tracking_holder,
    "guessing_holder": guessing_holder,
    "data_folder": data_folder
}

print("pickling tracking at frame {}...".format(i_frame))
with open(data_folder +'/tracked_behavior.pkl', 'wb+') as f:
    pickle.dump(tracked_behavior,f)
print("behavior tracking pickled!")
```

In [38]:

```
#%% Plot tracked data to see that everything is fine
plt.close('all')
plt.figure()

NNN = ['b','c','s','psi','theta','phi','x','y','z','b','c','s','theta','phi','x','y','z']
for ii,name in enumerate(NNN):
    plt.subplot(len(NNN),1,ii+1)
    index = np.arange(tracking_holder.shape[1])
    plt.plot(index[:i_frame],tracking_holder[ii,:i_frame])
    plt.plot(index[:i_frame],guessing_holder[ii,:i_frame])
    plt.ylabel(str(ii)+'_'+name)
plt.show()
```
