# Supplementary material for: Automatic mapping of multiplexed social receptive fields by deep learning and GPU-accelerated 3D videography
Source: Nat Commun. 2022 Feb 1;13:593. doi: 10.1038/s41467-022-28153-7 (PMC8807631; doi:10.1038/s41467-022-28153-7)
Supplement: Supplementary file 9 — Supplementary Software [file 41467_2022_28153_MOESM9_ESM.zip › ebbesen_froemke_2021_code/analysis/004_Plot_network_training.html]

004\_Plot\_network\_training


In [1]:

```
# plot the training performance!

import time
from pathlib import Path
import numpy as np
import matplotlib.pyplot as plt

import torch

import sys, os, pickle
import h5py
import cv2
from colour import Color
import glob, pathlib
import pandas as pd
```

# Load the loss function across training¶

In [2]:

```
# you will find the training runs in ./Runs/
# these training traces are just csv's exported from tensorboard, 
# They are exported to make a figrue for the manuscript -- you can just look at the runs in tensorboard

run_csv_folder = 'example_data/training_traces/'
files = os.listdir(run_csv_folder)
files_txt = [i for i in files if i.endswith('.csv')]
files_txt.sort()
for f in files_txt:
    print(f)
```

```
run-Nov22_11-19-19_CE-01-tag-lr(1).csv
run-Nov22_11-19-19_CE-01-tag-trn_frame_loss.csv
run-Nov22_11-19-19_CE-01-tag-trn_loss.csv
run-Nov22_11-19-19_CE-01-tag-val_frame_loss.csv
run-Nov22_11-19-19_CE-01-tag-val_loss.csv
```

In [3]:

```
val_frame_loss = pd.read_csv(glob.glob(run_csv_folder+'*val_frame_loss*')[0])
val_loss = pd.read_csv(glob.glob(run_csv_folder+'*val_loss*')[0])
trn_frame_loss = pd.read_csv(glob.glob(run_csv_folder+'*trn_frame_loss*')[0])
trn_loss = pd.read_csv(glob.glob(run_csv_folder+'*trn_loss*')[0])
print(val_frame_loss)
```

```
        Wall time    Step        Value
0    1.574440e+09       0  5138.962891
1    1.574440e+09       1  4729.284668
2    1.574440e+09       2  4470.230469
3    1.574440e+09       3  5048.220703
4    1.574440e+09       4  5750.927246
..            ...     ...          ...
665  1.574443e+09  133000   894.225952
666  1.574443e+09  133001  1448.598633
667  1.574443e+09  133002  1281.904907
668  1.574443e+09  133003  1145.183350
669  1.574443e+09  133004  1027.295776

[670 rows x 3 columns]
```

# Plot the training and validation loss¶

In [4]:

```
import matplotlib

# Say, "the default sans-serif font is COMIC SANS"
matplotlib.rcParams['font.sans-serif'] = "Liberation Sans"
# Then, "ALWAYS use sans-serif fonts"
matplotlib.rcParams['font.family'] = "sans-serif"

matplotlib.rc('font', family='sans-serif') 
matplotlib.rc('text', usetex='false') 
matplotlib.rcParams.update({'font.size': 13})
```

In [5]:

```
fig, (ax1, ax2) = plt.subplots(2, gridspec_kw={'height_ratios': [3, 1]}, sharex=False,figsize=(3.5,3))
from palettable.cmocean.sequential import Algae_6
cmpl = Algae_6.mpl_colors

def adjust_spines(ax, spines):
    for loc, spine in ax.spines.items():
        if loc in spines:
            spine.set_position(('outward', 10))  # outward by 10 points
            spine.set_smart_bounds(True)
        else:
            spine.set_color('none')  # don't draw spine

    # turn off ticks where there is no spine
    if 'left' in spines:
        ax.yaxis.set_ticks_position('left')
    else:
        # no yaxis ticks
        ax.yaxis.set_ticks([])

    if 'bottom' in spines:
        ax.xaxis.set_ticks_position('bottom')
    else:
        # no xaxis ticks
        ax.xaxis.set_ticks([])


def moving_average(data_set, periods=3):
    weights = np.ones(periods) / periods
    return np.convolve(data_set, weights, mode='same').ravel()

total_time = (trn_frame_loss['Wall time'].max()-trn_frame_loss['Wall time'].min())/60
time_stretch = total_time/len(trn_frame_loss['Value'])


# plt.subplot(4,1,(1,2,3) )
loss = trn_frame_loss['Value']
loss_ = loss.copy()
time = np.arange(len(loss)) * time_stretch
time_ = time.copy() 
# ax1.plot(time_,loss_,'.',c=cmpl[1],label="training batch",alpha = 1,markersize=4)

loss = val_frame_loss['Value']
scaling = len(loss_)/len(loss)
time = np.arange(len(loss))*scaling * time_stretch
ax1.plot(time,loss,'.',c=cmpl[4],label="validation batch",alpha = 1,markersize=4)
ax1.plot(time_,loss_,'.',c=cmpl[1],label="training batch",alpha = .5,markersize=4)

from scipy.signal import savgol_filter

ax = plt.gca()
ax1.spines['top'].set_visible(False)
ax1.spines['right'].set_visible(False)
ax1.spines['bottom'].set_visible(False)
ax2.spines['top'].set_visible(False)
ax2.spines['right'].set_visible(False)


# ax1.legend(loc='upper right')

ax1.set_ylim(300,10000)


ax1.set_yscale('log')
ax2.set_xlabel("Time [min]")
ax2.set_ylabel("Learning rate")
ax1.set_ylabel("Loss")

trn_lr = pd.read_csv(glob.glob(run_csv_folder+'*tag-lr*')[0])
time = np.arange(len(trn_lr))/len(trn_lr)*total_time
ax2.plot(time,trn_lr['Value'],c=cmpl[2])
print(len(trn_lr))

ax2.set_yscale('log')

# plt.xlim(0,45)
adjust_spines(ax1, ['left'])
adjust_spines(ax2, ['left', 'bottom'])

ax2.set_xticks([0,15,30,45,60])
ax2.set_yticks([1e-8,1e-3])

plt.show()
```

```
134
```
