# Supplementary material for: Automatic mapping of multiplexed social receptive fields by deep learning and GPU-accelerated 3D videography
Source: Nat Commun. 2022 Feb 1;13:593. doi: 10.1038/s41467-022-28153-7 (PMC8807631; doi:10.1038/s41467-022-28153-7)
Supplement: Supplementary file 9 — Supplementary Software [file 41467_2022_28153_MOESM9_ESM.zip › ebbesen_froemke_2021_code/analysis/009_Error checks_head_direction_and_running_behavior.html]

009\_Error checks\_head\_direction\_and\_running\_behavior


In [1]:

```
from IPython import get_ipython

# QT for movable plots
%load_ext autoreload
%autoreload 2

import time, os, sys, shutil
from utils.fitting_utils import *

# for math and plotting
import pandas as pd
import numpy as np
import scipy as sp
import matplotlib.pyplot as plt

from itertools import compress # for list selection with logical
from tqdm import tqdm

from multiprocessing import Process

# and pytorch
import torch

import sys, os, pickle
# import cv2
from colour import Color
import h5py
from tqdm import tqdm, tqdm_notebook
import glob
import itertools
```

In [2]:

```
from utils.analysis_tools import adjust_spines,cmpl
```

# Load the tracked data into memory¶

In [3]:

```
#load the tracked data!
data_folder = 'example_data/tracking/'

with open(data_folder +'tracked_behavior.pkl', 'rb') as f:
    tracked_behavior = pickle.load(f)
print(tracked_behavior.keys())

# load ALL the frames as jagged lines 
with h5py.File(data_folder+'pre_processed_frames.hdf5', mode='r') as hdf5_file:
    print(hdf5_file.keys())
    print(len(hdf5_file['dataset']))
    jagged_lines = hdf5_file['dataset'][...]
```

```
dict_keys(['var', 'ivar', 'body_constants', 'start_frame', 'end_frame', 'tracking_holder', 'guessing_holder', 'data_folder'])
<KeysViewHDF5 ['dataset']>
74962
```

In [4]:

```
from utils.cuda_tracking_utils import unpack_from_jagged, cheap4d
# kill first 6 secs of the frames (delay is ~180)
start_frame = 30*60
pos, pos_weights, keyp, pkeyp, ikeyp = unpack_from_jagged(jagged_lines[start_frame])
print(ikeyp)
print(pos.shape)

cheap4d(pos,keyp,ikeyp)
# fig = plt.gcf()
# plt.title("N positions is {}".format(pos.shape))
```

```
findfont: Font family ['cursive'] not found. Falling back to DejaVu Sans.
```

```
[0 1 1 2 3 0 1 1 1 2 2 0 2 0 1 1 1 2 3]
(2986, 3)
```

In [5]:

```
print(tracked_behavior['tracking_holder'].shape)
print(tracked_behavior['var'])
print(tracked_behavior['ivar'])
print(tracked_behavior.keys())
```

```
(18, 73361)
['b', 'c', 's', 'psi', 'theta', 'phi', 'x', 'y', 'z', 'b', 'c', 's', 'theta', 'phi', 'x', 'y', 'z']
['b0', 'c0', 's0', 'psi0', 'theta0', 'phi0', 'x0', 'y0', 'z0', 'b1', 'c1', 's1', 'theta1', 'phi1', 'x1', 'y1', 'z1']
dict_keys(['var', 'ivar', 'body_constants', 'start_frame', 'end_frame', 'tracking_holder', 'guessing_holder', 'data_folder'])
```

# Import 'PlotMachine' -- lots of handy plotting and analysis functions¶

In [20]:

```
from utils.analysis_tools import PlotMachine
# take a list of frames, calculate body supports and plot as a kind of decaying trail, with some decay and lengthm maybe the center of the nose??
plt.close('all')
Plotter = PlotMachine(tracked_behavior,jagged_lines)


frame = 30*60+1000+2000
Plotter.make(frame,view_override = [55.0,45.])
Plotter.make(frame,view_override = [95.0,40.])
Plotter.plot_residuals(frame)

n_fine = 5
```

```
4800
3200
i_trace_start is 2240 and i_frame is 3200
['nose']
['nose']
```

```
4800
3200
i_trace_start is 2240 and i_frame is 3200
['nose']
['nose']
```

# Plot data with full body, data with skeleton, only skeleton¶

In [21]:

```
if True:
    FFF = 30*60+20000-100
    Plotter.make_skel1(FFF)
    Plotter.make_skel2(FFF)
    Plotter.make_skel3(FFF)
#     Plotter.make_skel1(FFF, savepath = 'figure_raw_pics/figure_5_S/Full_BodyA.pdf')
#     Plotter.make_skel2(FFF, savepath = 'figure_raw_pics/figure_5_S/Full_BodyB.pdf')
#     Plotter.make_skel3(FFF, savepath = 'figure_raw_pics/figure_5_S/Full_BodyC.pdf')
```

# Import 'TrackingWrangler' -- handy functions for calculating features for movement and social analysis¶

In [23]:

```
from utils.analysis_tools import TrackingWrangler
     
Wrangler = TrackingWrangler(tracked_behavior,jagged_lines)
# Wrangler.kernel_smoothing()
Wrangler.unpack_all_body_support()
Wrangler.unpack_body_points()
# Wrangler.kernel_smoothing_points()

# Wrangler.calculate_2d_running(zoom = False, savepath='figure_raw_pics/figure_5_S/Running2D.pdf')
# Wrangler.plot_spatial_running(body_idx = 0,savepath='figure_raw_pics/figure_5_S/RunningFromAbove.pdf')
# Wrangler.plot_spatial_running(body_idx = 1,savepath='figure_raw_pics/figure_5_S/RunningFromAbove.pdf')
```

# Plot the distance to the implant¶

In [24]:

```
Wrangler.calculate_implant_distance()
```

```
100%|██████████| 73361/73361 [00:11<00:00, 6319.77it/s]
/home/chrelli/git/3d_sandbox/share_code/analysis/utils/analysis_tools.py:1852: MatplotlibDeprecationWarning: Adding an axes using the same arguments as a previous axes currently reuses the earlier instance.  In a future version, a new instance will always be created and returned.  Meanwhile, this warning can be suppressed, and the future behavior ensured, by passing a unique label to each axes instance.
  plt.subplot(n_subplots,1,1+subplot_counter)
```

# Plot the distance to the ears¶

In [26]:

```
Wrangler.calculate_ear_distance(zoom=True)
```

```
100%|██████████| 73361/73361 [00:26<00:00, 2720.50it/s]
```

```
pik
```

```
/home/chrelli/git/3d_sandbox/share_code/analysis/utils/analysis_tools.py:2008: MatplotlibDeprecationWarning: Adding an axes using the same arguments as a previous axes currently reuses the earlier instance.  In a future version, a new instance will always be created and returned.  Meanwhile, this warning can be suppressed, and the future behavior ensured, by passing a unique label to each axes instance.
```

```
x
y
z
```

```
/home/chrelli/git/3d_sandbox/share_code/analysis/utils/analysis_tools.py:2176: MatplotlibDeprecationWarning: Adding an axes using the same arguments as a previous axes currently reuses the earlier instance.  In a future version, a new instance will always be created and returned.  Meanwhile, this warning can be suppressed, and the future behavior ensured, by passing a unique label to each axes instance.
  for subplot_counter in range(n_subplots):
```

# Do some statistics on the 3D head direction¶

In [27]:

```
# MAKE some example plots of the joint distributions of 3D head angles and behaviors
v_ed = Wrangler.v_ed_smooth
v_rej = Wrangler.v_ed_reject_smooth
# normalize 
v_ed_norm = v_ed/np.linalg.norm(v_ed,axis =1)[:,np.newaxis]
v_rej_norm = v_rej/np.linalg.norm(v_rej,axis =1)[:,np.newaxis]

# normalize for plot
v_ed_norm = v_ed/np.linalg.norm(v_ed,axis =1)[:,np.newaxis]
v_rej_norm = v_rej/np.linalg.norm(v_rej,axis =1)[:,np.newaxis]

# z-coordinate
```

In [32]:

```
plt.figure(figsize = (7,7))
import matplotlib.gridspec as gridspec

gs = gridspec.GridSpec(3, 3)

dat = v_rej_norm[::50,:]
dat = v_rej_norm

def pairgrid_heatmap(x, y, **kws):
    cmap = sns.light_palette(kws.pop("color"), as_cmap=True)
    plt.hist2d(x, y, cmap=cmap, cmin=1, **kws)

# diagonals:
# lb = ['v$_{rej,x}$','v$_{rej,y}$','v$_{rej,z}$']
lb = ['v$_{x}$','v$_{y}$','v$_{z}$']

for i in range(3):
    ax = plt.subplot(gs[i,i])
    ax.hist(dat[:,i], bins=30,color = 'blueviolet',edgecolor = 'None',density = True)
    plt.ylim([0,6])
    plt.xlim([-1,1])
    plt.ylabel('density')
    ax.set_yticks([])
    plt.xlabel(lb[i])
    ax.spines['top'].set_visible(False)
    ax.spines['right'].set_visible(False)    
    

for i in range(3):
    for j in range(3):
        if i>=j:
            continue
        ax = plt.subplot(gs[i,j])
        ax.hist2d(dat[:,i], dat[:,j], cmap='Purples',bins = 30, cmin=0)
        plt.ylim([-1,1])
        plt.xlim([-1,1])     
        plt.xlabel(lb[i])
        ax.xaxis.labelpad = -10

        plt.ylabel(lb[j])
        ax.yaxis.labelpad = -10
        ax.set_xticks([-1,1])
        ax.set_yticks([-1,1])
        ax.spines['top'].set_visible(False)
        ax.spines['right'].set_visible(False)
        if j == 1:
            plt.plot([-.3,.3],[.9,.9],'k')
            plt.plot([-.3,-.3],[.9-.05,.9+.05],'k')
            plt.plot([.3,.3],[.9-.05,.9+.05],'k')

#         ax.spines['bottom'].set_visible(False)
#         ax.spines['left'].set_visible(False)

# plt.tight_layout()
# plt.savefig('figure_raw_pics/figure_5_S/SNS_jointA.pdf',transparent=True)
ss = .3

plt.subplots_adjust(wspace = ss,hspace = ss)
plt.show()
```

In [33]:

```
plt.figure(figsize = (4,2.4))
c_mid_1 = Wrangler.body_points[1][2]
z_mid = c_mid_1[:,2]

plt.hist(z_mid, bins=30,color = 'blueviolet',edgecolor = 'None',density = True)
plt.xlim([0,.1])
ax = plt.gca()
ax.set_xticks([0,.1])
ax.set_yticks([])
plt.ylabel('Density')
plt.xlabel('z$_{mid}$ [m]')
plt.axvline(.035,color = 'k')
ax.spines['top'].set_visible(False)
ax.spines['right'].set_visible(False)
plt.tight_layout()
# plt.savefig('figure_raw_pics/figure_5_S/SNS_jointB.pdf',transparent=True)

plt.show()
```

In [39]:

```
plt.figure(figsize = (6,6))
import matplotlib.gridspec as gridspec

gs = gridspec.GridSpec(2, 2)

dat = np.vstack([v_rej_norm[:,2],z_mid]).T
# print(dat)
dat = dat[z_mid>.035,:]

def pairgrid_heatmap(x, y, **kws):
    cmap = sns.light_palette(kws.pop("color"), as_cmap=True)
    plt.hist2d(x, y, cmap=cmap, cmin=1, **kws)

# diagonals:
# lb = ['v$_{rej,x}$','v$_{rej,y}$','v$_{rej,z}$']
lb = ['v$_{z}$','z$_{mid}$ [m]']

for i in range(2):
    ax = plt.subplot(gs[i,i])
    ax.hist(dat[:,i], bins=30,color = 'blueviolet',edgecolor = 'None',density = True)
    plt.ylim([0,6])
    if i == 0:
        plt.xlim([-1,1])
    if i == 1:
        plt.ylim([0,120])
        plt.xlim([.035,.1])
    plt.ylabel('density')
    ax.set_yticks([])
    plt.xlabel(lb[i])
    ax.spines['top'].set_visible(False)
    ax.spines['right'].set_visible(False)    
ax.set_xticks([.035,.1])

ax = plt.subplot(gs[0,1])    
ax.plot(dat[::50,1],dat[::50,0],'.',color = 'blueviolet',alpha = .5)
plt.ylim([-1,1])
plt.xlim([.035,.1])     
ax.set_yticks([-1,1])
ax.set_xticks([.035,.1])
plt.xlabel(lb[1])
plt.ylabel(lb[0])
ax.xaxis.labelpad = -10
ax.yaxis.labelpad = -10

ax.spines['top'].set_visible(False)
ax.spines['right'].set_visible(False)    

# plt.tight_layout()
# plt.savefig('figure_raw_pics/figure_5_S/SNS_jointC.pdf',transparent=True)
ss = .2
plt.subplots_adjust(wspace = ss,hspace = ss)

plt.show()
```

# Plot some example frames with the 3D head direction¶

In [41]:

```
Plotter.v_ed = Wrangler.v_ed_smooth
Plotter.v_ed_reject = Wrangler.v_ed_reject_smooth

Plotter.make_skel3(7000,view_override = [20.0,90.+180+30])
Plotter.make_skel3(6500,view_override = [20.0,90.+180+30])
Plotter.make_skel1(7000,view_override = [20.0,90.+180+30])
Plotter.make_skel1(6500,view_override = [20.0,90.+180+30])
Plotter.make_skel2(7000,view_override = [20.0,90.+180+30])
Plotter.make_skel2(6500,view_override = [20.0,90.+180+30])
```

# Also have a look at the running of the two mice¶

In [47]:

```
from utils.analysis_tools import TrackingWrangler

Wrangler = TrackingWrangler(tracked_behavior,jagged_lines)
# do a bit of smoothing here, for the running speed
Wrangler.kernel_smoothing()
Wrangler.unpack_all_body_support()
Wrangler.unpack_body_points()
Wrangler.kernel_smoothing_points()

Wrangler.calculate_2d_running(zoom =False)
```

In [ ]:

```

```
