# Supplementary material for: Automatic mapping of multiplexed social receptive fields by deep learning and GPU-accelerated 3D videography
Source: Nat Commun. 2022 Feb 1;13:593. doi: 10.1038/s41467-022-28153-7 (PMC8807631; doi:10.1038/s41467-022-28153-7)
Supplement: Supplementary file 9 — Supplementary Software [file 41467_2022_28153_MOESM9_ESM.zip › ebbesen_froemke_2021_code/analysis/010_State_space_modeling_and_detection_of_social_events.html]

010\_State\_space\_modeling\_and\_detection\_of\_social\_events


In [1]:

```
from IPython import get_ipython

# QT for movable plots
%load_ext autoreload
%autoreload 2

import time, os, sys, shutil
from utils.fitting_utils import *
    
# for math and plotting
import pandas as pd
import numpy as np
import scipy as sp
import matplotlib.pyplot as plt

from itertools import compress # for list selection with logical
from tqdm.notebook import tqdm

from multiprocessing import Process

# and pytorch
import torch

import sys, os, pickle
# import cv2
from colour import Color
import h5py
# from tqdm import tqdm, tqdm_notebook
import glob
import itertools
```

# Set up stuff for plotting, colors, fonts, etc.¶

In [2]:

```
# SET UP MATPLOTLIB
import matplotlib
import matplotlib.pyplot as plt
from mpl_toolkits.mplot3d import Axes3D

# Say, "the default sans-serif font is COMIC SANS"
matplotlib.rcParams['font.sans-serif'] = "Liberation Sans"
# Then, "ALWAYS use sans-serif fonts"
matplotlib.rcParams['font.family'] = "sans-serif"

matplotlib.rc('font', family='sans-serif') 
matplotlib.rc('text', usetex=False) 
matplotlib.rcParams.update({'font.size': 13})

# AHHH, Ok: https://stackoverflow.com/questions/11367736/matplotlib-consistent-font-using-latex
matplotlib.rcParams['mathtext.fontset'] = 'custom'
matplotlib.rcParams['mathtext.rm'] = 'Liberation Sans'
matplotlib.rcParams['mathtext.it'] = 'Liberation Sans:italic'
matplotlib.rcParams['mathtext.bf'] = 'Liberation Sans:bold'
matplotlib.rcParams['mathtext.fallback_to_cm'] = False
from palettable.cmocean.sequential import Algae_6
cmpl = Algae_6.mpl_colors
```

In [3]:

```
# setup for pretty plotting

import matplotlib

# Say, "the default sans-serif font is COMIC SANS"
matplotlib.rcParams['font.sans-serif'] = "Liberation Sans"
# Then, "ALWAYS use sans-serif fonts"
matplotlib.rcParams['font.family'] = "sans-serif"

matplotlib.rc('font', family='sans-serif') 
matplotlib.rc('text', usetex='false') 
matplotlib.rcParams.update({'font.size': 13})

from palettable.cmocean.sequential import Algae_6
cmpl = Algae_6.mpl_colors

def adjust_spines(ax, spines):
    for loc, spine in ax.spines.items():
        if loc in spines:
            spine.set_position(('outward', 10))  # outward by 10 points
            spine.set_smart_bounds(True)
        else:
            spine.set_color('none')  # don't draw spine

    # turn off ticks where there is no spine
    if 'left' in spines:
        ax.yaxis.set_ticks_position('left')
    else:
        # no yaxis ticks
        ax.yaxis.set_ticks([])

    if 'bottom' in spines:
        ax.xaxis.set_ticks_position('bottom')
    else:
        # no xaxis ticks
        ax.xaxis.set_ticks([])
```

In [4]:

```
from utils.analysis_tools import gradient_cmap
```

In [5]:

```
from palettable.wesanderson import Mendl_4,GrandBudapest5_5,Darjeeling2_5,Moonrise1_5
from palettable.cartocolors.qualitative import Pastel_10,Bold_10
from palettable.colorbrewer.qualitative import Set2_8
from palettable.tableau import TableauMedium_10

cmpl_wes = Moonrise1_5.mpl_colors
cmpl_qual = TableauMedium_10.mpl_colors

cmpl_qual[0]=TableauMedium_10.mpl_colors[1]
cmpl_qual[1]=TableauMedium_10.mpl_colors[0]
# colors = harvard_colors()
colors = cmpl_qual
# add the mendl colors 
# for i,state in enumerate(np.array([0,1,2,3])):
#     colors[state] = np.array(cmpl_mendl1[i])
    
# for i,state in enumerate(np.array([4,5,6])+1):
#     colors[state] = np.array(cmpl_wes[i])

cmap = gradient_cmap(colors)
# cmap = LinearSegmentedColormap('behav_colors', colors)
```

# Load the data, either from a 'TrackingWrangler' instace holding raw data, or -- like here for upload with the manuscipt -- from saved running trajectories¶

In [11]:

```
# Save data, handy for later! 

import pickle
loaded_data=pickle.load(open( "example_data/pyro_xyz.p", "rb" ) )
fit_fwd = loaded_data["fwd"]
fit_left = loaded_data["left"]
fit_up = loaded_data["up"]
fit_z = loaded_data["z"]
    
# zcore    
from scipy import stats
y_data = np.vstack((stats.zscore(fit_fwd),stats.zscore(fit_left))).T

# also get the whitening coefficients!
white_shift = np.array([np.mean(a) for a in [fit_fwd,fit_left]])
white_scale = np.array([np.std(a) for a in [fit_fwd,fit_left]])
```

# Plot a 2D histogram (log scale) of the running¶

In [14]:

```
from utils.analysis_tools import plot_2dhist_all

plot_2dhist_all(y_data)
```

# Use k-means to initialize the HMM¶

In [16]:

```
# set the number of states to fit and give them colors
data_dim = 2
hidden_dim = 5

cmpl_qual = TableauMedium_10.mpl_colors

cmpl_qual[0]=TableauMedium_10.mpl_colors[3]
cmpl_qual[3]=TableauMedium_10.mpl_colors[0]
# colors = harvard_colors()
colors = cmpl_qual
cmap = gradient_cmap(colors)

    
    
verbose = True
```

In [17]:

```
from utils.analysis_tools import fit_kmeans

data = torch.tensor(y_data[:,:2],dtype = torch.float)
data_np = y_data[:,:2]

mu_prior,std_prior = fit_kmeans(data,hidden_dim,colors)
```

```
'c' argument looks like a single numeric RGB or RGBA sequence, which should be avoided as value-mapping will have precedence in case its length matches with 'x' & 'y'.  Please use a 2-D array with a single row if you really want to specify the same RGB or RGBA value for all points.
'c' argument looks like a single numeric RGB or RGBA sequence, which should be avoided as value-mapping will have precedence in case its length matches with 'x' & 'y'.  Please use a 2-D array with a single row if you really want to specify the same RGB or RGBA value for all points.
'c' argument looks like a single numeric RGB or RGBA sequence, which should be avoided as value-mapping will have precedence in case its length matches with 'x' & 'y'.  Please use a 2-D array with a single row if you really want to specify the same RGB or RGBA value for all points.
'c' argument looks like a single numeric RGB or RGBA sequence, which should be avoided as value-mapping will have precedence in case its length matches with 'x' & 'y'.  Please use a 2-D array with a single row if you really want to specify the same RGB or RGBA value for all points.
'c' argument looks like a single numeric RGB or RGBA sequence, which should be avoided as value-mapping will have precedence in case its length matches with 'x' & 'y'.  Please use a 2-D array with a single row if you really want to specify the same RGB or RGBA value for all points.
```

```
prior mu: tensor([[ 0.3805,  2.9133],
        [ 0.9057,  0.0679],
        [ 3.0595, -0.3147],
        [-0.4526,  0.0669],
        [ 0.5209, -2.1550]]) and std: tensor([[1.2596, 1.5675],
        [0.5030, 0.6411],
        [0.8725, 1.2380],
        [0.3267, 0.4313],
        [0.9966, 1.2315]])
```

# Plot the starting guess on top of the behavior¶

In [18]:

```
# ONLY show the training data!

data = torch.tensor(y_data[:,:2],dtype = torch.float)
# data = torch.tensor(y_data[110000:130000,:2],dtype = torch.float)
# data = torch.tensor(y_data[80000:140000,:2],dtype = torch.float)
data = torch.tensor(y_data[75000:-10000,:2],dtype = torch.float)
data_np = data.numpy()

bins = (np.linspace(-4,6,10*4),np.linspace(-10,10,6*4))

# mu_prior,std_prior = fit_kmeans(data,hidden_dim,colors)

plt.figure(figsize = (4,2.7))
plt.hist2d(data_np[:,0],data_np[:,1],bins=bins,cmap='Greys',norm=matplotlib.colors.LogNorm())

plt.xlabel('Fwd speed [z]')
plt.ylabel('Left speed [z]')
plt.tight_layout()
ax = plt.gca()
# adjust_spines(ax,['Bottom','Left'])
ax.set_yticks([-8,0,8])
ax.set_xticks([-4,0,6])
plt.gca().spines['top'].set_visible(False)
plt.gca().spines['right'].set_visible(False)
plt.gca().spines['left'].set_bounds(-8,8)

plt.xlim([-4,6])
plt.ylim([-10,10])

for i in range(hidden_dim):
    plt.plot(mu_prior[i,0],mu_prior[i,1],'o',markersize = 7, c=colors[i])
    plt.plot(mu_prior[i,0] * np.ones(2),mu_prior[i,1]+np.array([-1,1])*std_prior[i,1].numpy(),'-', c=colors[i],lw=2.5)
    
    plt.plot(mu_prior[i,0]+np.array([-1,1])*std_prior[i,0].numpy(),mu_prior[i,1]*np.ones(2),'-', c=colors[i],lw = 2.5)

plt.savefig('figs/raw_histo.png',transparent = True,dpi=600)

plt.show()
```

# Cut the data in snippets for parallel fitting with Pyro¶

In [19]:

```
import os
import torch
import pyro
import pyro.distributions as dist
from torch.distributions import constraints
from pyro import poutine
from pyro.infer import SVI, Trace_ELBO, TraceEnum_ELBO, JitTraceEnum_ELBO, config_enumerate, infer_discrete
from pyro.infer.autoguide import AutoDiagonalNormal,init_to_sample,AutoMultivariateNormal,AutoDelta
from pyro.ops.indexing import Vindex
from collections import defaultdict
device = torch.device('cuda:0' if torch.cuda.is_available() else 'cpu')
device = 'cpu'

pyro.enable_validation()
pyro.set_rng_seed(1987)
```

In [20]:

```
# Trainid data is T x D= 5000 x D
# MAKE th training data into batch X T X D
# Let's make 20 batches?

seq_length = 600
data_idx = torch.arange(data.shape[0])
if data.shape[0]%seq_length == 0:
    split_data = torch.stack(torch.split(data,seq_length))
    split_data_idx = torch.stack(torch.split(data_idx,seq_length))
else:
    split_data = torch.stack( list(torch.split(data,seq_length))[:-1]) 
    split_data_idx = torch.stack( list(torch.split(data_idx,seq_length))[:-1])
```

In [21]:

```
# Figure! Plot training snippets!
plt.figure(figsize = (4,2.7))
axes = []
for i in range(2):
    ax = plt.subplot(2,1,1+i)
    ax.plot(data_idx,data[:,i],'k')
    axes.append(ax)
for a in range(split_data_idx.shape[0]):
    for i in range(2):
        c = cmpl[1+2*(a%2)] 
        axes[i].plot(split_data_idx[a,:],split_data[a,:,i],'-',c=c,alpha = 1.,lw=3)

        axes[i].set_xlim([0,10*600])        
    
plt.subplots_adjust(hspace=0)
    
for i in range(2):
    plt.subplot(2,1,1+i)
    
    ax = plt.gca()
    if i == 0:
        plt.ylabel('Fwd \nspeed\n[z]')
#         adjust_spines(ax,'Left')
    else:
        plt.ylabel('Left \nspeed\n[z]')
#         adjust_spines(ax,['Left','Bottom'])
        llim,ulim = ax.get_xlim()
        plt.plot(ulim+np.array([-60*10,0]),-4*np.ones(2),'k')   
    plt.ylim([-6,6])

        
    ax.set_yticks([-5,0,5])
#         ax.set_xticks([-4,0,6])
    plt.gca().spines['top'].set_visible(False)
    plt.gca().spines['right'].set_visible(False)        
    plt.gca().spines['bottom'].set_visible(False)        
    
    if i == 0:
        plt.gca().spines['bottom'].set_visible(False)        
    plt.xticks([])
    ax.spines['left'].set_bounds(-5, 5)


plt.xlabel('Frames')

plt.tight_layout()
plt.subplots_adjust(hspace=0)
plt.savefig('figs/training_snippets.png',transparent = True,dpi=600)
plt.show()
```

```
/home/chrelli/anaconda3/envs/myp/lib/python3.6/site-packages/ipykernel_launcher.py:18: MatplotlibDeprecationWarning: Adding an axes using the same arguments as a previous axes currently reuses the earlier instance.  In a future version, a new instance will always be created and returned.  Meanwhile, this warning can be suppressed, and the future behavior ensured, by passing a unique label to each axes instance.
```

# Define the HMM model in Pyro¶

In [22]:

```
# MAKE A PLATED
# THIS is a batched version
from pyro.util import ignore_jit_warnings

data_pyro = torch.tensor(data_np, dtype = torch.float,device=device)
verbose=True

# data_pyro_batch = split_data.shape
# for debugging
# data_pyro_batch = split_data[[0,0,0,0,0,0,0,0],:,:]
# data_pyro_batch = split_data[0,:,:].unsqueeze(0)
data_pyro_batch = split_data

if not (device == 'cpu'):
    data_pyro_batch.to(device)
data_pyro = torch.tensor(data_np, dtype = torch.float,device=device)

@config_enumerate
def hmm_model_batch(data,hidden_dim=hidden_dim,batch_size = None, device = device,verbose=False):
    # data has to be a tensor with batch x time x data_dtim
    with ignore_jit_warnings():
        num_segments, segment_length, data_dim = map(int, data.shape)
    if batch_size is None:
        batch_size = num_segments
    if verbose:
        print('Running for {} time steps'.format(len(data)))
    # Sample global matrices wrt a Jeffreys prior.
    with pyro.plate("hidden_state", hidden_dim):
        trans_dist = dist.Dirichlet(.5 * torch.ones(hidden_dim,dtype=torch.float,device=device))
        transition = pyro.sample("transition", trans_dist)
        # independent priors for the scale and mean of data dimensions
        locs = pyro.sample('locs', dist.Normal(torch.zeros(data_dim,dtype=torch.float,device=device), 2.* torch.ones(data_dim,dtype=torch.float,device=device) ) .independent() )
        scales = pyro.sample('scales', dist.LogNormal(0.*torch.ones(data_dim,dtype=torch.float,device=device),1.*torch.ones(data_dim,dtype=torch.float,device=device)) .independent() )
    #     scales = pyro.sample('scales', dist.HalfCauchy(0.01*torch.ones(data_dim,dtype=torch.float,device=device),1.*torch.ones(data_dim,dtype=torch.float,device=device)) .independent() )
    #     scales = pyro.sample('scales', dist.HalfCauchy(0.*torch.ones(data_dim,dtype=torch.float,device=device),1.*torch.ones(data_dim,dtype=torch.float,device=device)) .independent() )

        # make a diagonal matrix of the variances
    #         scale_matrix = scales.sqrt().diag_embed()
        scale_matrix = scales.diag_embed()
        # LKJ prior for the covariance
        # draw from lkj priors to make the covariance matrices
        eta = 5.*torch.ones(1,dtype=torch.float,device=device)
        lkj_priors = pyro.sample('lkj', dist.LKJCorrCholesky(data_dim, eta))  
        # and multiply the lkj and the scale matrix to get the lower triangle of the covariance matrix
        L_Omega = torch.bmm(scale_matrix , torch.bmm(lkj_priors,scale_matrix))

    if verbose:    
        print("transition is {}, shape: {}".format(transition,transition.shape))
        print("locs is {}, shape: {}".format(locs,locs.shape))
        print("scales is {}, shape: {}".format(scales,scales.shape))
        print("scale_matrix is {}, shape: {}".format(scale_matrix,scale_matrix.shape))
        print("lkj_priors is {}, shape: {}".format(lkj_priors,lkj_priors.shape))
        print("L_Omega is {}, shape: {}".format(L_Omega,L_Omega.shape))
    
    # select a subset of the possible batches, use automatic broadcasting
    with pyro.plate('batching',num_segments,batch_size) as batch:
        with poutine.scale(scale=1./batch_size):
            if verbose:
                print("these arae that batch indices:")
                print(batch)

            # Now, use the markov enumeration to observe all the data from a multivariate normal. 
            x = 0  # initial state
            for t in pyro.markov(range(segment_length)):
                x = pyro.sample("x_{}".format(t), dist.Categorical(transition[x]),
                                infer={"enumerate": "parallel"})
                pyro.sample("y_{}".format(t), dist.MultivariateNormal(locs[x,...], scale_tril=L_Omega[x,...]), 
                            obs=data[batch,t,...])
                if (t < 4) and verbose:
                    print("x_{}.shape = {}".format(t, x.shape))
                    print("x is: {}".format(x))
                    print("locs shape is :")
                    print(locs[x,...].shape)

hmm_model_batch(data_pyro_batch,verbose=False)
```

In [23]:

```
# TOD add a starting guess for the covariance, probably uncorrelated?
def my_init_fn(site):
    if site["name"] == "locs":
        return mu_prior.to(device)
    if site["name"] == "scales":
        return std_prior.to(device)
    # IF the site is not caugth above, we just init to a random sample from the prior
    if site["name"] == "transition":
        off_diag = 0.05
        transition_init = torch.eye(hidden_dim,dtype = torch.float,device = device)*(1-off_diag) +off_diag/hidden_dim
        return transition_init.to(device)
    if site["name"] == "lkj":
#         off_diag = 0.0001
#         lkj_single = torch.eye(data_dim,dtype = torch.float,device = device)*(1-off_diag) +off_diag/data_dim
        lkj_single = torch.eye(data_dim,dtype = torch.float,device = device)
        lkj_init = torch.stack(hidden_dim*[lkj_single],0)
        return lkj_init.to(device)    
    return init_to_sample(site)

from pyro.optim import Adam
# Make a data loader which takes all the 
my_init_fn({"name": "locs"})
```

Out[23]:

```
tensor([[ 0.3805,  2.9133],
        [ 0.9057,  0.0679],
        [ 3.0595, -0.3147],
        [-0.4526,  0.0669],
        [ 0.5209, -2.1550]])
```

In [26]:

```
# import a few plotting functions
from utils.analysis_tools import plot_cov_ellipse,plot_latent_ellisoids,plot_map_estimate
```

In [24]:

```
# def make_svi(hmm_model_batch,data_pyro_batch):
    # MAke a guide hmm_model_batch
#     hmm_guide = AutoDiagonalNormal(poutine.block(hmm_model_batch, expose=["transition", "locs","scales","lkj"]), 
#                                    init_loc_fn=my_init_fn).to(device)
hmm_guide = AutoDelta(poutine.block(hmm_model_batch, expose=["transition", "locs","scales","lkj"]), 
                                                    init_loc_fn=my_init_fn).to(device)

pyro.clear_param_store()
#     optim = pyro.optim.Adam({'lr': 0.005, 'betas': [0.8, 0.99]})
#     optim = pyro.optim.Adam({'lr': 0.01})

#     optim = pyro.optim.Adam({'lr': 0.05})
#     optim = pyro.optim.SGD({'lr': 0.001, 'momentum': 0.5})
optim = pyro.optim.SGD({'lr': 0.0005, 'momentum': 0.0})

# elbo = TraceEnum_ELBO(max_plate_nesting=1)
elbo = TraceEnum_ELBO(max_plate_nesting=2)
#     elbo = JitTraceEnum_ELBO(max_plate_nesting=2)

# elbo.loss(hmm_model, hmm_guide, data_pyro[:200], data_dim=data_dim);
#     batch_size = 50 
batch_size = data_pyro_batch.shape[0]
#     batch_size = 20
elbo.loss(hmm_model_batch, hmm_guide, data_pyro_batch,batch_size =  batch_size);
svi = SVI(hmm_model_batch, hmm_guide, optim, loss=elbo);


#     return svi, hmm_guide,elbo

# svi, hmm_guide,elbo = make_svi(hmm_model_batch,data_pyro_batch)
```

# Fit the HMM model¶

In [27]:

```
import time
# Register hooks to monitor gradient norms.
gradient_norms = defaultdict(list)
for name, value in pyro.get_param_store().named_parameters():
    value.register_hook(lambda g, name=name: gradient_norms[name].append(g.norm().item()))

losses = []
loss_time = []
map_esti_holder = []
# save the staring point
#     map_estimates =  hmm_guide(data_pyro_batch)

names = ['transition','locs','scales','lkj']
#     map_esti_holder.append( [ map_estimates[name].detach().cpu().numpy() for name in names] )
map_esti_holder.append( [my_init_fn({"name": name}).detach().cpu().numpy() for name in names] )

plot_map_estimate(map_esti_holder,colors,data_pyro.cpu().numpy(),0,savepath = None)

N_STEPS = 100
start_time= time.time()

for i in tqdm(range(N_STEPS)):
    loss = svi.step(data_pyro_batch)
    loss_time.append(time.time()-start_time)
    print("i: {} loss = {}".format(i,loss))

    losses.append(loss)
    map_estimates =  hmm_guide(data_pyro_batch)
    names = ['transition','locs','scales','lkj']
    map_esti_holder.append( [ map_estimates[name].detach().cpu().numpy() for name in names] )

    if i%20 == 0:
        # plot ever 20th step
        plot_map_estimate(map_esti_holder,colors,data_pyro.cpu().numpy(),0,savepath = None)
        plot_map_estimate(map_esti_holder,colors,data_pyro.cpu().numpy(),i,savepath = None)
        plt.figure(figsize = (2,2))
        plt.imshow(map_esti_holder[i][0])
        plt.show()


        #     print('.' if i % 2 else '\n', end='')
        from matplotlib import pyplot
        %matplotlib inline

        plt.figure(figsize=(3,2), dpi=100).set_facecolor('white')
        plt.plot(np.log(losses),'--o')
        plt.xlabel('iters')
        plt.ylabel('loss')
        plt.yscale('log')
        plt.title('Convergence of SVI');


        # plt plot the gradients converging
        plt.figure(figsize=(3,2), dpi=100).set_facecolor('white')
        for name, grad_norms in gradient_norms.items():
            plt.plot(grad_norms, label=name)
        plt.xlabel('iters')
        plt.ylabel('gradient norm')
        plt.yscale('log')
        plt.legend(loc='best')
        plt.title('Gradient norms during SVI');    


import pickle,time
now = time.time()
# todo, also pickle the training data and the initialization
pickle.dump( {"losses": losses,"loss_time": loss_time , "map": map_esti_holder,"data": data_pyro}, 
            open( "example_data/CPU_BATCH_pyro_fit_"+str(now)+".p", "wb" ) )

# return losses,loss_time,map_esti_holder,gradient_norms
# losses,loss_time,map_esti_holder,gradient_norms = pyro_routine(hmm_model_batch,data_pyro_batch,svi,hmm_guide,elbo)    

for i in [0,1,-1]:
# for i in range(len(losses)):
#     plot_map_estimate(map_esti_holder,colors,data_pyro_batch[0,...].cpu().numpy(),i,savepath = None)
    plot_map_estimate(map_esti_holder,colors,data_pyro.cpu().numpy(),i,savepath = None)
```

```
i: 0 loss = 1287.1181640625
```

```
i: 1 loss = 1143.6317138671875
i: 2 loss = 1086.8746337890625
i: 3 loss = 1052.2349853515625
i: 4 loss = 1030.6092529296875
i: 5 loss = 1024.2890625
i: 6 loss = 1020.6343994140625
i: 7 loss = 1017.839111328125
i: 8 loss = 1015.4512329101562
i: 9 loss = 1013.322265625
i: 10 loss = 1011.3645629882812
i: 11 loss = 1009.5355834960938
i: 12 loss = 1007.8073120117188
i: 13 loss = 1006.1642456054688
i: 14 loss = 1004.5956420898438
i: 15 loss = 1003.083251953125
i: 16 loss = 1001.6265258789062
i: 17 loss = 1000.21826171875
i: 18 loss = 998.8585815429688
i: 19 loss = 997.543212890625
```

```
findfont: Font family ['cursive'] not found. Falling back to DejaVu Sans.
```

```
i: 20 loss = 996.2714233398438
```

```
i: 21 loss = 995.0418090820312
i: 22 loss = 993.8544921875
i: 23 loss = 992.7037963867188
i: 24 loss = 991.5899658203125
i: 25 loss = 990.5082397460938
i: 26 loss = 989.4598388671875
i: 27 loss = 988.4417724609375
i: 28 loss = 987.4525146484375
i: 29 loss = 986.4910278320312
i: 30 loss = 985.5582885742188
i: 31 loss = 984.6510009765625
i: 32 loss = 983.7714233398438
i: 33 loss = 982.9169921875
i: 34 loss = 982.0869750976562
i: 35 loss = 981.2820434570312
i: 36 loss = 980.4983520507812
i: 37 loss = 979.7373046875
i: 38 loss = 978.9954833984375
i: 39 loss = 978.2753295898438
i: 40 loss = 977.5720825195312
```

```
i: 41 loss = 976.885498046875
i: 42 loss = 976.2161254882812
i: 43 loss = 975.564453125
i: 44 loss = 974.9329223632812
i: 45 loss = 974.3207397460938
i: 46 loss = 973.7281494140625
i: 47 loss = 973.1529541015625
i: 48 loss = 972.5982666015625
i: 49 loss = 972.06005859375
i: 50 loss = 971.537841796875
i: 51 loss = 971.030517578125
i: 52 loss = 970.5391235351562
i: 53 loss = 970.0612182617188
i: 54 loss = 969.5972290039062
i: 55 loss = 969.141845703125
i: 56 loss = 968.6987915039062
i: 57 loss = 968.2669067382812
i: 58 loss = 967.8447875976562
i: 59 loss = 967.436279296875
i: 60 loss = 967.0354614257812
```

```
i: 61 loss = 966.6455688476562
i: 62 loss = 966.2644653320312
i: 63 loss = 965.8934936523438
i: 64 loss = 965.5330200195312
i: 65 loss = 965.1819458007812
i: 66 loss = 964.837890625
i: 67 loss = 964.5042724609375
i: 68 loss = 964.1786499023438
i: 69 loss = 963.859375
i: 70 loss = 963.5494995117188
i: 71 loss = 963.2455444335938
i: 72 loss = 962.9489135742188
i: 73 loss = 962.6598510742188
i: 74 loss = 962.37744140625
i: 75 loss = 962.0993041992188
i: 76 loss = 961.828369140625
i: 77 loss = 961.5606079101562
i: 78 loss = 961.2999267578125
i: 79 loss = 961.042236328125
i: 80 loss = 960.7903442382812
```

```
i: 81 loss = 960.5418701171875
i: 82 loss = 960.2987670898438
i: 83 loss = 960.0618896484375
i: 84 loss = 959.82763671875
i: 85 loss = 959.6018676757812
i: 86 loss = 959.3768310546875
i: 87 loss = 959.1575317382812
i: 88 loss = 958.9415893554688
i: 89 loss = 958.7311401367188
i: 90 loss = 958.5225219726562
i: 91 loss = 958.3203735351562
i: 92 loss = 958.1196899414062
i: 93 loss = 957.9241943359375
i: 94 loss = 957.7290649414062
i: 95 loss = 957.53857421875
i: 96 loss = 957.3432006835938
i: 97 loss = 957.13916015625
i: 98 loss = 956.9373779296875
i: 99 loss = 956.7379150390625
```

In [32]:

```
# save the convergence plot
from utils.analysis_tools import plot_losses

savepath = 'figs/convergence_batch.png'
plot_losses(losses,savepath)

plot_map_estimate(map_esti_holder,colors,data_np,-1,
                  savepath = 'figs/pyro_fit.png')
```

# Assign states by MAP and plot example¶

In [29]:

```
# Use a viterbi decoder on the training data! And see if it looks good.
@infer_discrete(first_available_dim=-1, temperature=0)
@config_enumerate
def viterbi_decoder(data, transition_probs, means, covs,debug = False):
    states = [0]
    for t in pyro.markov(range(len(data))) :
        states.append(pyro.sample("states_{}".format(t),
                                  dist.Categorical(transition_probs[states[-1]])))
        if (t < 10) and debug:
            print("tr")
            print(transition_probs[states[-1]].shape)
            print(states[-1])
        pyro.sample("obs_{}".format(t),
                    dist.MultivariateNormal(means[states[-1]], scale_tril=covs[states[-1]]),
                    obs=data[t])
    return states  # returns maximum likelihood states


# And plot it!
transition,locs,scales,lkj = map_esti_holder[-1]
hidden_dim,data_dim = locs.shape

# Calculate the covariance
L_om = np.zeros([hidden_dim,data_dim,data_dim])
Sigma = np.zeros([hidden_dim,data_dim,data_dim])
#     print(scales)
# print(lkj)
for k in range(hidden_dim):
    # make a matrix of the scales
    s_matrix = np.diag( np.sqrt(scales[k,...]) )
    lkj_matrix=lkj[k,...]
    L_om[k,...] = s_matrix @ (lkj_matrix @ s_matrix)
    # mirror the lower to the upper!
    X = L_om[k,...]
    Sigma[k,...] = X + X.T - np.diag(np.diag(X))    

    
# example_data_pyro = data
example_data_pyro = torch.tensor(y_data[80000:85000,:], dtype = torch.float,device=device)
states = viterbi_decoder(example_data_pyro,torch.tensor(transition,dtype=torch.float),
                         torch.tensor(locs,dtype=torch.float),
                         torch.tensor(L_om,dtype=torch.float))    
hmm_state = torch.stack(states[1:])
```

In [30]:

```
def plot_colored_data(data_pyro,hmm_state,cmap):
    plt.figure(figsize = (10*1.5,1.5*1.2))
    # colors = ['r','g','b','orange']
    # and plot
    cmap_here = cmap
    cmap_here._init()
    alphas = 0.8
    # alphas = 1.0
    cmap_here._lut[:,-1] = alphas

    for i in range(2):
        plt.subplot(2,1,1+i)
        ax = plt.gca()
        plt.axhline(white_scale[i],ls='--',color='lightgrey')

        ax.plot(data_pyro[:,i],c='k',lw=2)
        llim,ulim = ax.get_ylim()
        ax.imshow(hmm_state[None,:], aspect="auto",cmap=cmap_here, vmin=0, vmax=len(colors)-1,  
                  extent=(0, data_pyro.shape[0], llim, ulim))

        if i == 0:
            plt.ylabel('Fwd\nspeed\n[z]')
        if i == 1:
            plt.ylabel('Left\nspeed\n[z]')
    #         plt.xlabel('Frame')

            _,xmax = ax.get_xlim()
            ymin,_ = ax.get_ylim()
            plt.plot(np.array([-5*60,0])+xmax,np.ones(2)*ymin,c='k',lw = 6)

        plt.xticks([])
        plt.yticks([])
        ax.spines['top'].set_visible(False)
        ax.spines['right'].set_visible(False)
        ax.spines['bottom'].set_visible(False)
        ax.spines['left'].set_visible(False)

    plt.tight_layout()    
    plt.subplots_adjust(hspace=0)
    savepath = 'figs/states_pyro_xy.png'
    plt.savefig(savepath,transparent = True,dpi=600)
    plt.show()

plot_colored_data(example_data_pyro,hmm_state,cmap)
```

# Decode all the data¶

In [31]:

```
# decode all the data!
all_data_pyro = torch.tensor(y_data[:,:], dtype = torch.float,device=device)
# do it chunk by chunk
all_split = torch.split(all_data_pyro,3000)
all_states = []
for d in tqdm(all_split):
    
    sta = viterbi_decoder(d,torch.tensor(transition,dtype=torch.float),
                         torch.tensor(locs,dtype=torch.float),
                         torch.tensor(L_om,dtype=torch.float))    
    all_states.append(torch.stack(sta[1:]))

all_states_xy = torch.cat(all_states)
print("viterbi done!")
```

```
viterbi done!
```

# Also fit an HMM model to the z speed. Start by cutting snippets for fitting¶

In [43]:

```
# Trainid data is T x D= 5000 x D
# MAKE th training data into batch X T X D
# Let's make 20 batches?

from scipy import stats
y_data_z = np.vstack((stats.zscore(fit_fwd),stats.zscore(fit_left),stats.zscore(fit_up))).T

# also get the whitening coefficients!
white_shift_z = np.array([np.mean(a) for a in [fit_fwd,fit_left,fit_up]])
white_scale_z = np.array([np.std(a) for a in [fit_fwd,fit_left,fit_up]])


data = torch.tensor(y_data_z[:,2],dtype = torch.float)
data = torch.tensor(y_data_z[75000:-10000,2],dtype = torch.float).unsqueeze(1)
data_np = data.numpy()

seq_length = 600
data_idx = torch.arange(data.shape[0])
if data.shape[0]%seq_length == 0:
    split_data = torch.stack(torch.split(data,seq_length))
    split_data_idx = torch.stack(torch.split(data_idx,seq_length))
else:
    split_data = torch.stack( list(torch.split(data,seq_length))[:-1]) 
    split_data_idx = torch.stack( list(torch.split(data_idx,seq_length))[:-1]) 

data_pyro_z = split_data    
# Figure! Plot training snippets!
plt.figure(figsize = (2.5*1.5,2*1.5))
axes = []
data_dim = data.shape[1]
for i in range(data_dim):
    ax = plt.subplot(2,1,1+i)
    ax.plot(data_idx,data[:,i],'k')
    axes.append(ax)
for a in range(split_data_idx.shape[0]):
    for i in range(data_dim):
        c = cmpl[1+2*(a%2)] 
        axes[i].plot(split_data_idx[a,:],split_data[a,:,i],'-',c=c,alpha = 1.,lw=4)

        axes[i].set_xlim([0,10*600])        
    
    
for i in range(data_dim):
    plt.subplot(2,1,1+i)
    
    ax = plt.gca()

    plt.ylabel('Up speed [z]')
#     adjust_spines(ax,['Left','Bottom'])
    llim,ulim = ax.get_xlim()
    plt.plot(ulim+np.array([-60*10,0]),-5*np.ones(2),'k')    
    
plt.subplots_adjust(hspace=0)
plt.gca().spines['left'].set_bounds(-5,5)
plt.gca().spines['top'].set_visible(False)
plt.gca().spines['right'].set_visible(False)
plt.gca().spines['bottom'].set_visible(False)
plt.xticks([])


plt.tight_layout()
plt.savefig('figs/training_snip_z.png',transparent = True,dpi=600)
plt.show()
```

```
/home/chrelli/anaconda3/envs/myp/lib/python3.6/site-packages/ipykernel_launcher.py:44: MatplotlibDeprecationWarning: Adding an axes using the same arguments as a previous axes currently reuses the earlier instance.  In a future version, a new instance will always be created and returned.  Meanwhile, this warning can be suppressed, and the future behavior ensured, by passing a unique label to each axes instance.
```

# Set # of states, initialize by k-means, and set colors¶

In [44]:

```
hidden_dim = 3

mu_prior,std_prior = fit_kmeans(data,hidden_dim,colors,show_plots = False)

cmpl_qual = TableauMedium_10.mpl_colors

cmpl_qual[0]=TableauMedium_10.mpl_colors[3]
cmpl_qual[3]=TableauMedium_10.mpl_colors[0]
cmpl_qual[5]=TableauMedium_10.mpl_colors[9]
cmpl_qual[6]=TableauMedium_10.mpl_colors[6]
cmpl_qual[7]=TableauMedium_10.mpl_colors[5]

cmpl_mendl = Moonrise1_5.mpl_colors

# colors = harvard_colors()
colors = cmpl_qual
cmap = gradient_cmap(colors)
```

```
prior mu: tensor([-1.5430e-03,  2.8986e+00, -3.0013e+00]) and std: tensor([[0.4785],
        [1.0434],
        [1.0959]])
```

In [45]:

```
# Plot the HMM initialization on top of the data
```

In [46]:

```
plt.figure(figsize = [3.5,2.3])
plt.hist(data_np,100,color = 'lightgrey',density = True)
# plt.yscale('log')
plt.xlim([-6,6])
plt.xlabel('Up speed [z]')
plt.ylabel('')

yy = .4
for i in range(len(mu_prior)):
    plt.plot(mu_prior[i],yy,'o',color = colors[i+5],markersize = 7)
    plt.plot(mu_prior[i]+std_prior[i]*np.array([-1,1]),yy*np.ones(2),'-',color = colors[i+5],lw=3)

plt.yticks([0,1])
plt.gca().set_yticklabels('')

ax = plt.gca()
# adjust_spines(ax,['Bottom','Left'])
plt.gca().spines['top'].set_visible(False)
plt.gca().spines['right'].set_visible(False)
plt.ylabel('Density')
plt.xticks([-5,0,5])
plt.gca().spines['bottom'].set_bounds(-5,5)


plt.tight_layout()
# plt.yscale('log')
plt.savefig('figs/training_z.png',transparent = True,dpi=600)
plt.show()
```

# Declare the HMM model in Pyro¶

In [47]:

```
@config_enumerate
def hmm_model_z(data, data_dim=data_dim, hidden_dim=hidden_dim,batch_size = None,verbose=False):
    # data has to be a tensor with batch x time x data_dtim
    with ignore_jit_warnings():
        num_segments, segment_length, data_dim = map(int, data.shape)
    if batch_size is None:
        batch_size = num_segments
    if verbose:
        print('Running for {} time steps'.format(len(data)))

    # Sample global matrices wrt a Jeffreys prior.
    with pyro.plate("hidden_state", hidden_dim):
        transition = pyro.sample("transition", dist.Dirichlet(0.5 * torch.ones(hidden_dim)))

    locs = pyro.sample('locs', dist.Normal(torch.zeros(hidden_dim), 2.* torch.ones(hidden_dim) ).independent()  )
    scales = pyro.sample('scales', dist.LogNormal(torch.ones(hidden_dim)*0.,torch.ones(hidden_dim)*1. ).independent() )
    
    if verbose:
        print("shapes")
        print(transition.shape)
        print(locs.shape)
        print(scales.shape)

    # select a subset of the possible batches, use automatic broadcasting
    with pyro.plate('batching',num_segments,batch_size) as batch:
        with poutine.scale(scale=1./batch_size):        
            x = 0  # initial state
            for t in pyro.markov(range(segment_length)):
                x = pyro.sample("x_{}".format(t), dist.Categorical(transition[x]),
                                infer={"enumerate": "parallel"})
                if (t < 4) and verbose:
                    print("x is {}".format(x))
                    print("locs and scales")
                    print(locs[x])
                    print(scales[x])
                    print('data sample:')
                    print(data[batch,t,...].squeeze())
                    print("OBS")
                    hmm=pyro.sample("hmm_{}".format(t), dist.Normal(locs[x],scales[x]))
                    print(hmm)

                pyro.sample("y_{}".format(t), dist.Normal(locs[x],scales[x]), obs=data[batch,t,...].squeeze() )
                if (t < 4) and verbose:
                    print("x_{}.shape = {}".format(t, x.shape))


            
# hmm_model_z(data_pyro_z,verbose=True)
```

In [48]:

```
# TOD add a starting guess for the covariance, probably uncorrelated?
def my_init_fn_z(site):
    if site["name"] == "locs":
        return mu_prior.to(device)
    if site["name"] == "scales":
        return std_prior.to(device).squeeze()
    # IF the site is not caugth above, we just init to a random sample from the prior
    if site["name"] == "transition":
        off_diag = 0.05
        transition_init = torch.eye(hidden_dim,dtype = torch.float,device = device)*(1-off_diag) +off_diag/hidden_dim
        return transition_init.to(device)
    if site["name"] == "lkj":
#         off_diag = 0.0001
#         lkj_single = torch.eye(data_dim,dtype = torch.float,device = device)*(1-off_diag) +off_diag/data_dim
        lkj_single = torch.eye(data_dim,dtype = torch.float,device = device)
        lkj_init = torch.stack(hidden_dim*[lkj_single],0)
        return lkj_init.to(device)    
    return init_to_sample(site)

from pyro.optim import Adam
# Make a data loader which takes all the 
my_init_fn({"name": "locs"})
my_init_fn({"name": "scales"})
# my_init_fn({"name": "transition"})
# my_init_fn({"name": "lkj"})
```

Out[48]:

```
tensor([[0.4785],
        [1.0434],
        [1.0959]])
```

In [49]:

```
# def make_svi(hmm_model_batch,data_pyro_batch):
    # MAke a guide hmm_model_batch
#     hmm_guide = AutoDiagonalNormal(poutine.block(hmm_model_batch, expose=["transition", "locs","scales","lkj"]), 
#                                    init_loc_fn=my_init_fn).to(device)


hmm_guide = AutoDelta(poutine.block(hmm_model_z, expose=["transition", "locs","scales"]), 
                      init_loc_fn=my_init_fn_z).to(device)


# hmm_guide = AutoDelta(poutine.block(hmm_model_z, expose=["transition", "locs","scales"])).to(device)

pyro.clear_param_store()

#     optim = pyro.optim.Adam({'lr': 0.005, 'betas': [0.8, 0.99]})
#     optim = pyro.optim.Adam({'lr': 0.01})

#     optim = pyro.optim.Adam({'lr': 0.05})
#     optim = pyro.optim.SGD({'lr': 0.001, 'momentum': 0.5})
optim = pyro.optim.SGD({'lr': 0.0005, 'momentum': 0.5})

# elbo = TraceEnum_ELBO(max_plate_nesting=1)
elbo = TraceEnum_ELBO(max_plate_nesting=2)
#     elbo = JitTraceEnum_ELBO(max_plate_nesting=2)

# elbo.loss(hmm_model, hmm_guide, data_pyro[:200], data_dim=data_dim);
#     batch_size = 50 
batch_size = data_pyro_z.shape[0]
#     batch_size = 20
elbo.loss(hmm_model_z, hmm_guide, data_pyro_z,batch_size =  batch_size);
svi = SVI(hmm_model_z, hmm_guide, optim, loss=elbo);


#     return svi, hmm_guide,elbo

# svi, hmm_guide,elbo = make_svi(hmm_model_batch,data_pyro_batch)
```

# Run fitting for z¶

In [50]:

```
from utils.analysis_tools import plot_map_estimate_z

# def pyro_routine(hmm_model_batch,data_pyro_batch,svi,hmm_guide,elbo):
import time
# Register hooks to monitor gradient norms.
gradient_norms = defaultdict(list)
for name, value in pyro.get_param_store().named_parameters():
    value.register_hook(lambda g, name=name: gradient_norms[name].append(g.norm().item()))

losses_z = []
loss_time = []
map_esti_holder_z = []
# save the staring point
#     map_estimates =  hmm_guide(data_pyro_batch)

names = ['transition','locs','scales']
#     map_esti_holder.append( [ map_estimates[name].detach().cpu().numpy() for name in names] )
map_esti_holder_z.append( [my_init_fn({"name": name}).detach().cpu().numpy() for name in names] )

# plot_map_estimate(map_esti_holder,colors,data_pyro.cpu().numpy(),0,savepath = None)

N_STEPS = 100
start_time= time.time()

for i in tqdm(range(N_STEPS)):
    loss = svi.step(data_pyro_z)
    loss_time.append(time.time()-start_time)
    print("i: {} loss = {}".format(i,loss))

    losses_z.append(loss)
    map_estimates =  hmm_guide(data_pyro_z)
    map_esti_holder_z.append( [ map_estimates[name].detach().cpu().numpy() for name in names] )


    if i%20 == 0:
        plot_map_estimate_z(map_esti_holder_z,colors,data_np,0,savepath=None)
        plot_map_estimate_z(map_esti_holder_z,colors,data_np,i,savepath=None)
                    
        plt.figure(figsize = (2,2))
        plt.imshow(map_esti_holder[i][0])
        plt.show()

        #     print('.' if i % 2 else '\n', end='')
        from matplotlib import pyplot
        %matplotlib inline

        plt.figure(figsize=(3,2), dpi=100).set_facecolor('white')
        plt.plot(np.log(losses_z),'--o')
        plt.xlabel('iters')
        plt.ylabel('loss')
        plt.yscale('log')
        plt.title('Convergence of SVI');


        # plt plot the gradients converging
        plt.figure(figsize=(3,2), dpi=100).set_facecolor('white')
        for name, grad_norms in gradient_norms.items():
            plt.plot(grad_norms, label=name)
        plt.xlabel('iters')
        plt.ylabel('gradient norm')
        plt.yscale('log')
        plt.legend(loc='best')
        plt.title('Gradient norms during SVI');    


import pickle,time
now = time.time()
# todo, also pickle the training data and the initialization
pickle.dump( {"losses": losses_z,"loss_time": loss_time , "map": map_esti_holder_z,"data": data_pyro}, 
            open( "CPU_BATCH_pyro_fit_z_"+str(now)+".p", "wb" ) )

# return losses,loss_time,map_esti_holder,gradient_norms
# losses,loss_time,map_esti_holder,gradient_norms = pyro_routine(hmm_model_batch,data_pyro_batch,svi,hmm_guide,elbo)    

# for i in [0,1,-1]:
#     plot_map_estimate(map_esti_holder,colors,data_pyro.cpu().numpy(),i,savepath = None)
```

```
i: 0 loss = 491.5104064941406
```

```
i: 1 loss = 479.4921569824219
i: 2 loss = 467.7342529296875
i: 3 loss = 460.2534484863281
i: 4 loss = 455.6927185058594
i: 5 loss = 452.3441162109375
i: 6 loss = 449.32110595703125
i: 7 loss = 446.6486511230469
i: 8 loss = 444.4122619628906
i: 9 loss = 442.5534973144531
i: 10 loss = 440.96258544921875
i: 11 loss = 439.5574645996094
i: 12 loss = 438.2910461425781
i: 13 loss = 437.1376037597656
i: 14 loss = 436.0760803222656
i: 15 loss = 435.0919189453125
i: 16 loss = 434.17010498046875
i: 17 loss = 433.3027038574219
i: 18 loss = 432.4845886230469
i: 19 loss = 431.70904541015625
i: 20 loss = 430.9735107421875
```

```
i: 21 loss = 430.2749328613281
i: 22 loss = 429.6098327636719
i: 23 loss = 428.97650146484375
i: 24 loss = 428.3744812011719
i: 25 loss = 427.8009338378906
i: 26 loss = 427.2564697265625
i: 27 loss = 426.7401428222656
i: 28 loss = 426.2514953613281
i: 29 loss = 425.78887939453125
i: 30 loss = 425.3505859375
i: 31 loss = 424.9371032714844
i: 32 loss = 424.5447692871094
i: 33 loss = 424.1756896972656
i: 34 loss = 423.8258056640625
i: 35 loss = 423.49517822265625
i: 36 loss = 423.18316650390625
i: 37 loss = 422.888427734375
i: 38 loss = 422.6097106933594
i: 39 loss = 422.345947265625
i: 40 loss = 422.09735107421875
```

```
i: 41 loss = 421.8621520996094
i: 42 loss = 421.64068603515625
i: 43 loss = 421.4302673339844
i: 44 loss = 421.2319030761719
i: 45 loss = 421.0438537597656
i: 46 loss = 420.8661193847656
i: 47 loss = 420.69659423828125
i: 48 loss = 420.5366516113281
i: 49 loss = 420.3835144042969
i: 50 loss = 420.239013671875
i: 51 loss = 420.1004333496094
i: 52 loss = 419.9678649902344
i: 53 loss = 419.8422546386719
i: 54 loss = 419.72235107421875
i: 55 loss = 419.6073303222656
i: 56 loss = 419.4975280761719
i: 57 loss = 419.3928527832031
i: 58 loss = 419.29351806640625
i: 59 loss = 419.19647216796875
i: 60 loss = 419.10516357421875
```

```
i: 61 loss = 419.0166931152344
i: 62 loss = 418.9320983886719
i: 63 loss = 418.8525390625
i: 64 loss = 418.77508544921875
i: 65 loss = 418.7012634277344
i: 66 loss = 418.63031005859375
i: 67 loss = 418.5628662109375
i: 68 loss = 418.4980163574219
i: 69 loss = 418.43701171875
i: 70 loss = 418.377685546875
i: 71 loss = 418.3211975097656
i: 72 loss = 418.2669982910156
i: 73 loss = 418.2140808105469
i: 74 loss = 418.1650085449219
i: 75 loss = 418.1173400878906
i: 76 loss = 418.07012939453125
i: 77 loss = 418.02679443359375
i: 78 loss = 417.9840087890625
i: 79 loss = 417.9435729980469
i: 80 loss = 417.9046325683594
```

```
i: 81 loss = 417.86663818359375
i: 82 loss = 417.830078125
i: 83 loss = 417.7952880859375
i: 84 loss = 417.7611999511719
i: 85 loss = 417.7296447753906
i: 86 loss = 417.6973876953125
i: 87 loss = 417.66680908203125
i: 88 loss = 417.6369323730469
i: 89 loss = 417.6084289550781
i: 90 loss = 417.58038330078125
i: 91 loss = 417.553955078125
i: 92 loss = 417.5279235839844
i: 93 loss = 417.5028991699219
i: 94 loss = 417.4779968261719
i: 95 loss = 417.4551696777344
i: 96 loss = 417.4317626953125
i: 97 loss = 417.40911865234375
i: 98 loss = 417.38677978515625
i: 99 loss = 417.36663818359375
```

In [51]:

```
# save figs
savepath = 'figs/convergence_z.png'
plot_losses(losses_z,savepath,z=True)

plot_map_estimate_z(map_esti_holder_z,colors,data_np,i=-1,
    savepath='figs/gaussians_pyro_z.png')
```

# Also decode all the z data¶

In [52]:

```
# USE a viterbi algorithm to filter the data!
@infer_discrete(first_available_dim=-1, temperature=0)
@config_enumerate
def viterbi_decoder_z(data,transition,locs,scales):
    # takes the transition matrix and the inferred locations
    states = [0]
    for t in pyro.markov(range(len(data))):
        states.append(pyro.sample("states_{}".format(t),
                                  dist.Categorical(transition[states[-1]])))
        pyro.sample("obs_{}".format(t),
                    dist.Normal(locs[states[-1]], scales[states[-1]]),
                    obs=data[t])
    return states  # returns maximum likelihood states
```

In [53]:

```
transition,locs,scales = map_esti_holder_z[-1]

# decode all the data!
all_data_pyro = torch.tensor(y_data_z[:,2], dtype = torch.float,device=device)

all_split = torch.split(all_data_pyro,3000)
all_states_z = []
for d in tqdm(all_split):
    
    sta = viterbi_decoder_z(d,torch.tensor(transition,dtype=torch.float),
                         torch.tensor(locs,dtype=torch.float),
                         torch.tensor(scales,dtype=torch.float))    
    all_states_z.append(torch.stack(sta[1:]))

all_states_z = torch.cat(all_states_z)

print("viterbi done!")
```

```
viterbi done!
```

# And make an example plot for z¶

In [54]:

```
# Make an example 
all_states_z2= all_states_z+5
# all_states_z2[all_states_z2==7] = 5
# all_states_z2[all_states_z2==5] = 9
# all_states_z2[all_states_z2==7] = 9

def plot_colored_data_z(data_pyro,hmm_state,cmap):
    plt.figure(figsize = (10*1.5,1.5*1.2))
    # colors = ['r','g','b','orange']
    # and plot
    cmap_here = cmap
    cmap_here._init()
    alphas = 0.8
    # alphas = 1.0
    cmap_here._lut[:,-1] = alphas

    for i in [0]:
        plt.subplot(2,1,2+i)
        ax = plt.gca()
        plt.axhline(white_scale[i],ls='--',color='lightgrey')

        ax.plot(data_pyro[:,i],c='k',lw=2)
        llim,ulim = ax.get_ylim()
#         hmm_state[hmm_state == 1]=6
#         hmm_state[hmm_state == 2]=7
#         hmm_state[hmm_state == 3]=8
        
        ax.imshow(hmm_state[None,:], aspect="auto",cmap=cmap_here, vmin=0, vmax=len(colors)-1,  
                  extent=(0, data_pyro.shape[0], llim, ulim))

#         if i == 0:
#             plt.ylabel('Fwd\nspeed\n[z]')
        if i == 0:
            plt.ylabel('Up \nspeed\n[z]')
    #         plt.xlabel('Frame')

            _,xmax = ax.get_xlim()
            ymin,_ = ax.get_ylim()
            plt.plot(np.array([-5*60,0])+xmax,np.ones(2)*ymin,c='k',lw = 6)

        plt.xticks([])
        plt.yticks([])
        ax.spines['top'].set_visible(False)
        ax.spines['right'].set_visible(False)
        ax.spines['bottom'].set_visible(False)
        ax.spines['left'].set_visible(False)

    plt.tight_layout()    
    plt.subplots_adjust(hspace=0)
    savepath = 'figs/states_pyro_z.png'
    plt.savefig(savepath,transparent = True,dpi=600)
    plt.show()

plot_colored_data_z(all_data_pyro[80000:85000,np.newaxis],all_states_z2[80000:85000],cmap)
```

# Save the ethograms for later¶

In [55]:

```
# SAVE everything, for later
import pickle,time
now = time.time()
# todo, also pickle the training data and the initialization
pickle.dump( {"losses": losses,"losses_z": losses_z , "mapxy": map_esti_holder,"mapz": map_esti_holder_z,
              "state_xy":all_states_xy,"state_z":all_states_z,"data": data_pyro}, 
            open( "example_data/pyro_fits.p", "wb" ) )

# can reload just for plotting 
# dict = pickle.load( open( "BOTH_BATCH_pyro_fit_z_"+str(now)+".p", "rb" ) )
```

# Plot the latent states for xy¶

In [57]:

```
## ALSO make a plot in the real speed space
from utils.analysis_tools import adjust_spines2

transition,locs,scales,lkj = map_esti_holder[-1]
hidden_dim,data_dim = locs.shape

# Calculate the covariance
L_om = np.zeros([hidden_dim,data_dim,data_dim])
Sigma = np.zeros([hidden_dim,data_dim,data_dim])
#     print(scales)
# print(lkj)
for k in range(hidden_dim):
    # make a matrix of the scales
    s_matrix = np.diag( np.sqrt(scales[k,...]) )
    lkj_matrix=lkj[k,...]
    L_om[k,...] = s_matrix @ (lkj_matrix @ s_matrix)
    # mirror the lower to the upper!
    X = L_om[k,...]
    Sigma[k,...] = X + X.T - np.diag(np.diag(X))    

# Plot samples from the fitted HMM model!
# state 0ne
n_states = hidden_dim
fig5 = plt.figure(constrained_layout=True,figsize = (4,4))
spec5 = fig5.add_gridspec(ncols=2, nrows=2)
axes = []
z_trans_mean = np.array([np.mean(fit_fwd), np.mean(fit_left)])
z_trans_std = np.array([np.std(fit_fwd-np.mean(fit_fwd)), np.std(fit_left-np.mean(fit_left))])

for state in range(n_states):
    mean = locs[state,...]
    cov = Sigma[state,...]
    ax = fig5.add_subplot(spec5[:])
    axes.append(ax)
#     ax = fig5.add_subplot(spec5[0,0])
    x = np.random.multivariate_normal(mean,cov,1000)
    #transform to speeds: 
    speeds = z_trans_std * x + z_trans_mean
    
#     ax.plot(speeds[:,0],speeds[:,1],'.k',markersize = .1)
    
    
#     ax.plot(fit_left[hmm_state_xy==state][::100],fit_fwd[hmm_state_xy==state][::100],'.',c= colors[state],markersize = 1)
    
    
    # move to the real space
    mean_scaled = z_trans_std * mean + z_trans_mean
    cov_scaled = z_trans_std[:,np.newaxis]@ z_trans_std[np.newaxis,:] * cov
    
    # and transform for plotting
    mean_scaled = mean_scaled[[1,0]]
    cov_scaled[[0,1],[0,1]] = cov_scaled[[1,0],[1,0]]
    
    

    for nstd in range(4):
        ellip = plot_cov_ellipse(cov_scaled, mean_scaled, nstd=nstd, ax=ax)
    #     ellip.set_edgecolor(colors[state])    
    #     ellip.set_facecolor('w')   
        ellip.set_edgecolor(colors[state])    
        ellip.set_facecolor(colors[state])   
        ellip.set_alpha(.2)


for state in range(n_states):
    mean = locs[state,...]
    cov = Sigma[state,...]
    
    # move to the real space
    mean_scaled = z_trans_std * mean + z_trans_mean
    cov_scaled = z_trans_std[:,np.newaxis]@ z_trans_std[np.newaxis,:] * cov
    
    # and transform for plotting
    mean_scaled = mean_scaled[[1,0]]
    cov_scaled[[0,1],[0,1]] = cov_scaled[[1,0],[1,0]]
    
    
    ax = axes[state]

    
    ellip4 = plot_cov_ellipse(cov_scaled, mean_scaled, nstd=3, ax=ax)
#     ellip3.set_edgecolor(colors[state])    
#     ellip3.set_facecolor('None')    
    ellip4.set_edgecolor(colors[state])    
    ellip4.set_facecolor('None')   
    #ellip4.set_alpha(1)    
    
    ax.set_xlim([-.1,.1])
    ax.set_ylim([-.05,.15])
    
    ax.set_xlim([-.11,.11])
    ax.set_ylim([-.06,.16])
    
    ax.set_xlim([-.11-.02,.11+.02])
    ax.set_ylim([-.06-.02,.16+.02])
    
    
    ax.set_xticks([-.1,-.05,0,.05,.1])
    ax.set_yticks([-.05,0,.05,.1,.15])
    ax.set_yticks([-.05,0,.05,.1,.15])


    ax.set_aspect('equal', 'box')
    
for state in range(n_states):
    mean = locs[state,...]
    cov = Sigma[state,...]
    
    # move to the real space
    mean_scaled = z_trans_std * mean + z_trans_mean
    cov_scaled = z_trans_std[:,np.newaxis]@ z_trans_std[np.newaxis,:] * cov
    
    # and transform for plotting
    mean_scaled = mean_scaled[[1,0]]
    cov_scaled[[0,1],[0,1]] = cov_scaled[[1,0],[1,0]]
    
    
    ax = axes[state]
#     ax.plot(mean_scaled[0],mean_scaled[1],'o',ms=20,color = colors[state])
    

    
#     ax.axhline(0,c='grey')
#     ax.axvline(0,c='grey')

for state in range(n_states):
    ax.plot(fit_left[all_states_xy==state][::100],fit_fwd[all_states_xy==state][::100],'.',c= colors[state],markersize = 2)

ax.set_xlabel('Left speed [m/s]')
ax.set_ylabel('Fwd speed [m/s]')
ax.invert_xaxis()

adjust_spines2(ax,['left','bottom'])
ax.spines['left'].set_bounds(ax.get_yticks()[0],ax.get_yticks()[-1])
ax.spines['bottom'].set_bounds(ax.get_xticks()[0],ax.get_xticks()[-1])

savepath = 'figs/states_xy_together_pyro.png'
plt.savefig(savepath,transparent=True,dpi = 600)   

plt.show()
```

```
/home/chrelli/anaconda3/envs/myp/lib/python3.6/site-packages/ipykernel_launcher.py:33: MatplotlibDeprecationWarning: Adding an axes using the same arguments as a previous axes currently reuses the earlier instance.  In a future version, a new instance will always be created and returned.  Meanwhile, this warning can be suppressed, and the future behavior ensured, by passing a unique label to each axes instance.
```

# Plot the distribution of durations of behaviors¶

In [72]:

```
def rle(stateseq):
    """
    # from: https://github.com/slinderman/ssm/blob/master/ssm/util.py
    Compute the run length encoding of a discrete state sequence.
    E.g. the state sequence [0, 0, 1, 1, 1, 2, 3, 3]
         would be encoded as ([0, 1, 2, 3], [2, 3, 1, 2])
    [Copied from pyhsmm.util.general.rle]
    Parameters
    ----------
    stateseq : array_like
        discrete state sequence
    Returns
    -------
    ids : array_like
        integer identities of the states
    durations : array_like (int)
        length of time in corresponding state
    """
    pos, = np.where(np.diff(stateseq) != 0)
    pos = np.concatenate(([0],pos+1,[len(stateseq)]))
    return stateseq[pos[:-1]], np.diff(pos)


# look at at the durations!
inferred_state_list, inferred_durations = rle(all_states_xy)
num_states = hidden_dim
fps = 60
# Rearrange the lists of durations to be a nested list where
# the nth inner list is a list of durations for state n
inf_durs_stacked = []
for s in range(num_states):
    inf_durs_stacked.append(inferred_durations[inferred_state_list == s] * (1/fps))

for s in range(num_states):
    inf_durs_stacked[s] = np.log10(inf_durs_stacked[s])
    
fig5 = plt.figure(constrained_layout=True,figsize = (3.5,2))
for state in range(num_states):
    bins = np.linspace(-2,2,60)
    count,edges = np.histogram(inf_durs_stacked[state],bins,density = True)
    plt.fill_between(edges[:-1],count,color = colors[state],alpha = .2)
    
for state in range(num_states):
    bins = np.linspace(-2,2,60)
    count,edges = np.histogram(inf_durs_stacked[state],bins,density = True)    
    plt.plot(edges[:-1],count,color = colors[state])
    
ax = plt.gca()
ax.set_xticks([-2,-1,-0,1,2])
ax.set_yticks([0,2.2])
ax.set_xticklabels(["0.01","0.1",'1','10','100'])
ax.set_yticklabels([])
ax.set_xlabel("Duration [s]")
ax.set_ylabel("Density")
# adjust_spines(ax,['left','bottom'])


adjust_spines2(ax,['left','bottom'])
ax.spines['left'].set_bounds(ax.get_yticks()[0],ax.get_yticks()[-1])
ax.spines['bottom'].set_bounds(ax.get_xticks()[0],ax.get_xticks()[-1])


savepath = 'figs/durations_xy_pyro.png'
plt.savefig(savepath,transparent=True,dpi = 600)   
plt.show()
```

# Plot the height vs duration of xy bouts¶

In [73]:

```
# plot the average z value of the z speed!
start_index = np.cumsum(inferred_durations)
z_holder = []
for st,d in tqdm(zip(start_index,inferred_durations)):
    z_holder.append(np.mean(fit_z[st:(st+d)]))
z_holder = np.hstack(z_holder)

z_holder_stacked = []
for s in range(num_states):
    z_holder_stacked.append(z_holder[inferred_state_list == s] )

fig5 = plt.figure(constrained_layout=True,figsize = (4,2))
for s in range(num_states):
    plt.plot(inf_durs_stacked[s],z_holder_stacked[s],'.',c = colors[s],alpha = .5)
    
ax = plt.gca()
ax.set_xticks([-2,-1,-0,1,2])
# ax.set_yticks([0,2.2])
ax.set_xticklabels(["0.01","0.1",'1','10','100'])
# ax.set_yticklabels([])
ax.set_xlabel("Duration [s]")
ax.set_ylabel("<z> [m]")


adjust_spines(ax,['left','bottom'])
# ax.spines['left'].set_bounds(ax.get_yticks()[0],ax.get_yticks()[-1])
# ax.spines['bottom'].set_bounds(ax.get_xticks()[0],ax.get_xticks()[-1])

savepath = 'figs/height_xy.png'
plt.savefig(savepath,transparent=False,dpi = 600)   
plt.show()
```

```
/home/chrelli/anaconda3/envs/myp/lib/python3.6/site-packages/numpy/core/fromnumeric.py:3335: RuntimeWarning: Mean of empty slice.
  out=out, **kwargs)
/home/chrelli/anaconda3/envs/myp/lib/python3.6/site-packages/numpy/core/_methods.py:161: RuntimeWarning: invalid value encountered in true_divide
  ret = ret.dtype.type(ret / rcount)
```

```

```

In [75]:

```
# look at p-values for the running. 
# The durations 
plt.figure()
inf_durs_stacked = []
for s in range(num_states):
    inf_durs_stacked.append(inferred_durations[inferred_state_list == s] * (1/fps))
    
    dat = inf_durs_stacked[s]
    plt.plot(dat*0.+s+np.random.normal(scale=.1, size=len(dat)),dat,'o',color = colors[s])

print('rest(3) vs all')
for s in range(num_states):
    print(sp.stats.mannwhitneyu(inf_durs_stacked[s],inf_durs_stacked[3]))

print('ffwd(2) vs all')
for s in range(num_states):
    print(sp.stats.mannwhitneyu(inf_durs_stacked[s],inf_durs_stacked[2]))    

print('medians')
for s in range(num_states):
    print(np.median(inf_durs_stacked[s])   )
```

```
rest(3) vs all
MannwhitneyuResult(statistic=50605.5, pvalue=2.6354951678709483e-11)
MannwhitneyuResult(statistic=117893.5, pvalue=2.9406147853420427e-26)
MannwhitneyuResult(statistic=69007.5, pvalue=0.011720673232448586)
MannwhitneyuResult(statistic=195312.5, pvalue=0.49996874009111264)
MannwhitneyuResult(statistic=71815.5, pvalue=2.3644862954500334e-11)
ffwd(2) vs all
MannwhitneyuResult(statistic=19174.5, pvalue=1.2740463033636969e-09)
MannwhitneyuResult(statistic=45503.0, pvalue=1.2329914610857238e-16)
MannwhitneyuResult(statistic=30012.5, pvalue=0.49987269511205923)
MannwhitneyuResult(statistic=69007.5, pvalue=0.011720673232448586)
MannwhitneyuResult(statistic=27777.0, pvalue=1.5128598837302994e-08)
medians
0.5
0.45
0.6833333333333333
0.8333333333333334
0.5166666666666666
```

# Plot the latent states for z¶

In [77]:

```
transition,locs,scales = map_esti_holder_z[-1]
hidden_dim = locs.shape[0]

fig5 = plt.figure(constrained_layout=True,figsize = (3.5,2))
spec5 = fig5.add_gridspec(ncols=1, nrows=1)
axes = []

# plt.subplots_adjust(hspace=0,wspace = 0)
n_states = hidden_dim

z_trans_mean = np.mean(fit_up)
z_trans_std = np.std(fit_up-np.mean(fit_up))

from scipy.stats import norm

for state in range(n_states):
    mean = locs[state]
    cov = scales[state]
#     x = np.random.multivariate_normal(mean,cov,1000)
    x = np.random.normal(loc = mean, scale = cov,size  = 1000)
    speeds = z_trans_std * x + z_trans_mean

    mean_scaled = z_trans_std * mean + z_trans_mean
    cov_scaled = z_trans_std * cov
    print(mean_scaled)
    print(cov)
    print(cov_scaled)
    
    for i,var in enumerate(['up']):
        ax = plt.gca()
        axes.append(ax)
       
        xxx = np.linspace(-.1,.1,400)
        yyy = norm.pdf(xxx,loc = mean_scaled,scale=cov_scaled)
        yyy = yyy/(np.sum(yyy)*np.median(np.diff(xxx))) *20
        ax.fill_between(xxx,yyy,facecolor = colors[state+5],alpha = .3)
#         ax.plot(xxx,yyy,c = colors[state+3],alpha = 1)
    
        ed = np.linspace(-.1,.1,1000)
        count,edges = np.histogram(fit_up[all_states_z2==state+5],ed,density = False)
#         ax.bar(edges[:-1],count,width = .001,edgecolor = 'None',alpha = .3)
        
        ax.plot(edges[:-1],count,color = colors[state+5],lw = 2)
    

    
    
#         ax.set_title(var)
#         ax.axvline(0,c='k')

        ax.set_xlabel('Up speed [m/s]')
        ax.set_ylabel('Density')
        ax.set_xlim([-.05,.05])
#         ax.set_ylim([0,400])
        ax.set_yticks([0,4000])
        ax.set_yticklabels([''])

    
adjust_spines(axes[0],['left','bottom'])

savepath = 'figs/states_z.png'
plt.savefig(savepath,transparent=True,dpi=600)   
plt.show()
```

```
-2.5600725968046127e-05
0.27700922
0.0026945696248520984
0.018888993269586856
1.2555648
0.01221333646945761
-0.0193401827531138
1.3167293
0.012808305835289488
```

# Plot the duration of z bouts¶

In [79]:

```
# look at at the durations!
inferred_state_list, inferred_durations = rle(all_states_z)
num_states = 3
fps = 60
# Rearrange the lists of durations to be a nested list where
# the nth inner list is a list of durations for state n
inf_durs_stacked = []
for s in range(num_states):
    inf_durs_stacked.append(inferred_durations[inferred_state_list == s] * (1/fps))

for s in range(3):
    inf_durs_stacked[s] = np.log10(inf_durs_stacked[s])
    
fig5 = plt.figure(constrained_layout=True,figsize = (3.5,2))

for state in range(3):
    bins = np.linspace(-2,2,60)
    count,edges = np.histogram(inf_durs_stacked[state],bins,density = True)
    plt.fill_between(edges[:-1],count,color = colors[state+5],alpha = .2)
    
for state in range(3):
    bins = np.linspace(-2,2,60)
    count,edges = np.histogram(inf_durs_stacked[state],bins,density = True)    
    plt.plot(edges[:-1],count,color = colors[state+5])
    
ax = plt.gca()
ax.set_xticks([-2,-1,-0,1,2])
ax.set_yticks([0,2.2])
ax.set_xticklabels(["0.01","0.1",'1','10','100'])
ax.set_yticklabels([])
ax.set_xlabel("Duration [s]")
ax.set_ylabel("Density")
adjust_spines(ax,['left','bottom'])

savepath = 'figs/durations_z.png'
plt.savefig(savepath,transparent=True,dpi=600)   
plt.show()
```

# And the height vs the duration¶

In [80]:

```
# plot the average z value of the z speed!
start_index = np.cumsum(inferred_durations)
z_holder = []
for st,d in tqdm(zip(start_index,inferred_durations)):
    z_holder.append(np.mean(fit_z[st:(st+d)]))
z_holder = np.hstack(z_holder)

z_holder_stacked = []
for s in range(num_states):
    z_holder_stacked.append(z_holder[inferred_state_list == s] )

fig5 = plt.figure(constrained_layout=True,figsize = (3.5,2))
for s in range(3):
    plt.plot(inf_durs_stacked[s],z_holder_stacked[s]*100,'.',c = colors[s+5],alpha = .2,markersize = 7)
    
ax = plt.gca()
ax.set_xticks([-2,-1,-0,1,2])
# ax.set_yticks([0,2.2])
ax.set_xticklabels(["0.01","0.1",'1','10','100'])
# ax.set_yticklabels([])
ax.set_xlabel("Duration [s]")
ax.set_ylabel("<z> [cm]")
adjust_spines(ax,['left','bottom'])
savepath = 'figs/height_z.png'
plt.savefig(savepath,transparent=True,dpi = 600)   
plt.show()
```

```
/home/chrelli/anaconda3/envs/myp/lib/python3.6/site-packages/numpy/core/fromnumeric.py:3335: RuntimeWarning: Mean of empty slice.
  out=out, **kwargs)
/home/chrelli/anaconda3/envs/myp/lib/python3.6/site-packages/numpy/core/_methods.py:161: RuntimeWarning: invalid value encountered in true_divide
  ret = ret.dtype.type(ret / rcount)
```

```

```

In [89]:

```
# calculate the spearman correlation between the height and the duration of the 'rest' bouts
plt.figure(figsize = [6,2])
s = 0
plt.plot(inf_durs_stacked[s],z_holder_stacked[s]*100,'.',c = colors[s+5],alpha = 1,markersize = 7)
plt.show()    

print(sp.stats.spearmanr(inf_durs_stacked[s],z_holder_stacked[s]*100))
```

```
SpearmanrResult(correlation=-0.47035985035130895, pvalue=9.412231714049152e-47)
```

# Calculate the emperical transition matrices for xy and z¶

In [91]:

```
def transition_matrix(transitions):
    # from https://stackoverflow.com/questions/46657221/generating-markov-transition-matrix-in-python
    n = 1+ max(transitions) #number of states

    
    M = [[0]*n for _ in range(n)]

    for (i,j) in zip(transitions,transitions[1:]):
        M[i][j] += 1

    #now convert to probabilities:
    for row in M:
        s = sum(row)
        if s > 0:
            row[:] = [f/s for f in row]
    return M

#test:


tm_xy = transition_matrix(all_states_xy)
print('empirical xy transitions:')
for row in tm_xy: print(' '.join('{0:.3f}'.format(x) for x in row))
tm_z = transition_matrix(all_states_z)
print('empirical z transitions:')
for row in tm_z: print(' '.join('{0:.3f}'.format(x) for x in row))
```

```
empirical xy transitions:
0.971 0.006 0.006 0.015 0.002
0.002 0.972 0.007 0.015 0.003
0.002 0.015 0.980 0.000 0.003
0.002 0.003 0.000 0.993 0.002
0.001 0.005 0.004 0.016 0.973
empirical z transitions:
0.993 0.004 0.004
0.025 0.975 0.000
0.027 0.000 0.973
```

# Plot the transition matrices¶

In [92]:

```
# plot the transition matrices!
fig = plt.figure(constrained_layout=False,figsize = (8,4))

plt.subplot(121)
learned_transition_mat = np.array(tm_xy)
im = plt.imshow(learned_transition_mat, cmap='gray')
# plt.title("Learned Transition Matrix")

for i in range(learned_transition_mat.shape[0]):
    plt.plot(i+np.array([-.5,.5]),np.array([-.5,-.5]),c=colors[i],lw=10)
    plt.plot(np.array([-.5,-.5]),i+np.array([-.5,.5]),c=colors[i],lw=10)
# ax = plt.gca()
# ax.axis('off')
plt.axis('off')

plt.subplot(122)
learned_transition_mat = np.array(tm_z)
im = plt.imshow(learned_transition_mat, cmap='gray')
# plt.title("Learned Transition Matrix")

# plot the 
# plt.plot([-.5,.5],[.5,.5],'r',lw=5)

for i in range(learned_transition_mat.shape[0]):
    plt.plot(i+np.array([-.5,.5]),np.array([-.5,-.5]),c=colors[i+5],lw=10)
    plt.plot(np.array([-.5,-.5]),i+np.array([-.5,.5]),c=colors[i+5],lw=10)

plt.axis('off')
# ax = plt.gca()
# ax.axis('off')


cbar_ax = fig.add_axes([0.95, 0.15, 0.05, 0.7])
# fig.colorbar(im1, cax=cbar_ax)
fig.colorbar(im, cax=cbar_ax)
plt.subplot(121)
plt.clim([0,1])
plt.subplot(122)
plt.clim([0,1])
plt.axis('off')

savepath = 'figs/transitions.png'
plt.savefig(savepath,transparent=True)   
plt.show()
```

```
/home/chrelli/anaconda3/envs/myp/lib/python3.6/site-packages/ipykernel_launcher.py:36: MatplotlibDeprecationWarning: Adding an axes using the same arguments as a previous axes currently reuses the earlier instance.  In a future version, a new instance will always be created and returned.  Meanwhile, this warning can be suppressed, and the future behavior ensured, by passing a unique label to each axes instance.
/home/chrelli/anaconda3/envs/myp/lib/python3.6/site-packages/ipykernel_launcher.py:38: MatplotlibDeprecationWarning: Adding an axes using the same arguments as a previous axes currently reuses the earlier instance.  In a future version, a new instance will always be created and returned.  Meanwhile, this warning can be suppressed, and the future behavior ensured, by passing a unique label to each axes instance.
```

In [93]:

```
# plot the transition matrices!
fig = plt.figure(constrained_layout=False,figsize = (8,4))

# maxi = np.max([np.max(hmm_xy.transitions.transition_matrix),np.max(hmm_z.transitions.transition_matrix)])

plt.subplot(121)
learned_transition_mat = np.array(tm_xy)
learned_transition_mat[np.arange(learned_transition_mat.shape[0]),np.arange(learned_transition_mat.shape[0])] = 0
im = plt.imshow(learned_transition_mat, cmap='gray')
# plt.title("Learned Transition Matrix")
for i in range(learned_transition_mat.shape[0]):
    plt.plot(i+np.array([-.5,.5]),np.array([-.5,-.5]),c=colors[i],lw=10)
    plt.plot(np.array([-.5,-.5]),i+np.array([-.5,.5]),c=colors[i],lw=10)
    
    plt.plot(i+np.array([-.5,.5]),i+np.array([-.5,.5]),c=colors[i],lw=5)
    plt.plot(i+np.array([.5,-.5]),i+np.array([-.5,.5]),c=colors[i],lw=5)

# ax = plt.gca()  
# ax.axis('off')

plt.axis('off')
plt.subplot(122)
learned_transition_mat = np.array(tm_z)
learned_transition_mat[np.arange(learned_transition_mat.shape[0]),np.arange(learned_transition_mat.shape[0])] = 0
im = plt.imshow(learned_transition_mat, cmap='gray')
# plt.title("Learned Transition Matrix")

for i in range(learned_transition_mat.shape[0]):
    plt.plot(i+np.array([-.5,.5]),np.array([-.5,-.5]),c=colors[i+5],lw=10)
    plt.plot(np.array([-.5,-.5]),i+np.array([-.5,.5]),c=colors[i+5],lw=10)

    plt.plot(i+np.array([-.5,.5]),i+np.array([-.5,.5]),c=colors[i+5],lw=5)
    plt.plot(i+np.array([.5,-.5]),i+np.array([-.5,.5]),c=colors[i+5],lw=5)


plt.axis('off')


cbar_ax = fig.add_axes([0.95, 0.15, 0.05, 0.7])
fig.colorbar(im, cax=cbar_ax)

plt.subplot(121)
plt.clim([0,.03])
plt.subplot(122)
plt.clim([0,.03])

savepath = 'figs/transitions_masked.png'
plt.savefig(savepath,transparent=True)   

plt.show()
```

```
/home/chrelli/anaconda3/envs/myp/lib/python3.6/site-packages/ipykernel_launcher.py:42: MatplotlibDeprecationWarning: Adding an axes using the same arguments as a previous axes currently reuses the earlier instance.  In a future version, a new instance will always be created and returned.  Meanwhile, this warning can be suppressed, and the future behavior ensured, by passing a unique label to each axes instance.
/home/chrelli/anaconda3/envs/myp/lib/python3.6/site-packages/ipykernel_launcher.py:44: MatplotlibDeprecationWarning: Adding an axes using the same arguments as a previous axes currently reuses the earlier instance.  In a future version, a new instance will always be created and returned.  Meanwhile, this warning can be suppressed, and the future behavior ensured, by passing a unique label to each axes instance.
```

# Finally, do a massive plot with all the states labeled for the whole recording¶

In [94]:

```
#load the tracked data!
data_folder = 'example_data/tracking/'

with open(data_folder +'tracked_behavior.pkl', 'rb') as f:
    tracked_behavior = pickle.load(f)
print(tracked_behavior.keys())

# load ALL the frames as jagged lines 
with h5py.File(data_folder+'/pre_processed_frames.hdf5', mode='r') as hdf5_file:
    print(hdf5_file.keys())
    print(len(hdf5_file['dataset']))
    jagged_lines = hdf5_file['dataset'][...]
```

```
dict_keys(['var', 'ivar', 'body_constants', 'start_frame', 'end_frame', 'tracking_holder', 'guessing_holder', 'data_folder'])
<KeysViewHDF5 ['dataset']>
74962
```

In [98]:

```
# Calculate running speed, social distances, etc etc
from utils.analysis_tools import PlotMachine, TrackingWrangler
plt.close('all')
Plotter = PlotMachine(tracked_behavior,jagged_lines)

Wrangler = TrackingWrangler(tracked_behavior,jagged_lines)
Wrangler.kernel_smoothing()
Wrangler.unpack_all_body_support()
Wrangler.unpack_body_points()
Wrangler.kernel_smoothing_points()

Wrangler.calculate_social_distances(hidden = True)
Wrangler.calculate_2d_running(zoom =False,plot=False)
```

```
/home/chrelli/git/3d_sandbox/share_code/analysis/utils/analysis_tools.py:1397: MatplotlibDeprecationWarning: Adding an axes using the same arguments as a previous axes currently reuses the earlier instance.  In a future version, a new instance will always be created and returned.  Meanwhile, this warning can be suppressed, and the future behavior ensured, by passing a unique label to each axes instance.
  plt.subplot(n_subplots,1,1+i)
```

In [100]:

```
# setup for pretty plotting

import matplotlib

# Say, "the default sans-serif font is COMIC SANS"
matplotlib.rcParams['font.sans-serif'] = "Liberation Sans"
# Then, "ALWAYS use sans-serif fonts"
matplotlib.rcParams['font.family'] = "sans-serif"

matplotlib.rc('font', family='sans-serif') 
matplotlib.rc('text', usetex='false') 
matplotlib.rcParams.update({'font.size': 13})

from palettable.cmocean.sequential import Algae_6
cmpl = Algae_6.mpl_colors

def adjust_spines(ax, spines):
    for loc, spine in ax.spines.items():
        if loc in spines:
            spine.set_position(('outward', 10))  # outward by 10 points
            spine.set_smart_bounds(True)
        else:
            spine.set_color('none')  # don't draw spine

    # turn off ticks where there is no spine
    if 'left' in spines:
        ax.yaxis.set_ticks_position('left')
    else:
        # no yaxis ticks
        ax.yaxis.set_ticks([])

    if 'bottom' in spines:
        ax.xaxis.set_ticks_position('bottom')
    else:
        # no xaxis ticks
        ax.xaxis.set_ticks([])
```

In [102]:

```
self = Wrangler # this is a bit hacky, todo: move to module later

from scipy import ndimage

zoom = False
if zoom:
    fig5 = plt.figure(constrained_layout=True,figsize = (4,10))
    savepath = 'figs/pyro_ethogram_social_zoom.png'

else:
    fig5 = plt.figure(constrained_layout=True,figsize = (18,10))
    savepath = 'figs/pyro_ethogram_social.png'


heights = np.ones(9)
n_subplots = len(heights)
spec5 = fig5.add_gridspec(ncols=1, nrows=n_subplots, height_ratios=heights)


axes = []

n_frames = int(len(all_states_z)/2)

# lol
idx_holder = [np.arange(n_frames),np.arange(2*n_frames)[n_frames:]]

# remember correct placement of idx here
y_data = np.vstack((sp.stats.zscore(fit_fwd),sp.stats.zscore(fit_left))).T
plot_data = np.vstack((fit_fwd,fit_left)).T
hmm_state_xy = all_states_xy

# plot the running 

subs = [0,1,2,3]
body_idxs = [0,0,1,1]
ii = [0,1,0,1]
names = ['fwd','left','fwd','left']

for (sub,body_idx,i,name) in zip(subs,body_idxs,ii,names):

    ax = fig5.add_subplot(spec5[sub,0])
    axes.append(ax)

    # apply the fitted model
    frame_idx = idx_holder[body_idx]

    # and plot
    ax.plot(plot_data[frame_idx,i],label=name,c='k')
    llim,ulim = ax.get_ylim()
    h_im = ax.imshow(hmm_state_xy[None,frame_idx], aspect="auto", cmap=cmap, vmin=0, vmax=len(colors)-1,  
              extent=(0, len(frame_idx), llim, ulim))
    h_im.set_alpha(.00)
#     ax.legend()
    ax.set_ylabel(name+" speed\n[m/s]")
    ax.axhline(0,c='grey')


    
    

# remember correct placement of idx here
y_data = np.vstack((sp.stats.zscore(fit_fwd),sp.stats.zscore(fit_left))).T
plot_data = np.vstack((fit_fwd,fit_left)).T
hmm_state_z = all_states_xy

# plot the running 

subs = [0,1,2,3]
body_idxs = [0,0,1,1]
ii = [0,1,0,1]
names = ['fwd','left','fwd','left']

# frick from here: https://stackoverflow.com/questions/10127284/overlay-imshow-plots-in-matplotlib
cmap_here = cmap
cmap_here._init()
alphas = 0.8
# alphas = 1.0
cmap_here._lut[:,-1] = alphas

for (sub,body_idx,i,name) in zip(subs,body_idxs,ii,names):

    ax = fig5.add_subplot(spec5[sub,0])
    axes.append(ax)

    # apply the fitted model
    frame_idx = idx_holder[body_idx]

    # and plot
    ax.plot(plot_data[frame_idx,i],label=name,c='k')
    llim,ulim = ax.get_ylim()
    ax.imshow(hmm_state_xy[None,frame_idx], aspect="auto", cmap=cmap_here, vmin=0, vmax=len(colors)-1,  
              extent=(0, len(frame_idx), llim, ulim))
#     ax.legend()
    ax.set_ylabel("Mouse"+str(body_idx)+"\n"+name+"\nspeed\n[m/s]")
    ax.axhline(0,c='grey')    
    

    
    
# apply the fitted model
y_data = sp.stats.zscore(fit_up)[:,np.newaxis]
hmm_state_z = all_states_z +5
plot_data = fit_up[:,np.newaxis]


subs = [4,5]
body_idxs = [0,1]
ii = [0,0]
names = ['up','up']

for (sub,body_idx,i,name) in zip(subs,body_idxs,ii,names):
    ax = fig5.add_subplot(spec5[sub,0])
    axes.append(ax)
    # apply the fitted model
    frame_idx = idx_holder[body_idx]
    ax.plot(plot_data[frame_idx,i],label=name,c='k')
    ax.set_ylim([-.1,.1])
    llim,ulim = ax.get_ylim()
    ax.imshow(hmm_state_z[None,frame_idx], aspect="auto", cmap=cmap, vmin=0, vmax=len(colors)-1,  
              extent=(0, len(frame_idx), llim, ulim))
#     ax.legend()
    ax.set_ylabel("Mouse"+str(body_idx)+"\n"+name+"\nspeed\n[m/s]")
    ax.axhline(0,c='grey')    
    
    
        
plt.subplots_adjust(hspace=0.05)

from palettable.wesanderson import Mendl_4,GrandBudapest5_5,Darjeeling2_5,Moonrise1_5

colors_social = Darjeeling2_5.mpl_colors
# colors_social = Mendl_4.mpl_colors
# colors_social = np.vstack(np.array(c) for c in colors_social)

color_names = [
    "windows blue",
    "red",
    "amber",
    "faded green",
    "light violet",
    "orange",
    "light pink"
    ]


# cmap_social = gradient_cmap([colors_social[i] for i in [0,2,3]])
cmap_social = gradient_cmap(colors_social)


d_list = [self.d_nose2nose,self.d_nose02ass,self.d_nose12ass]
d_frames = []
names = ['Nose0-\nNose1','Nose0-\nTail1','Nose1-\nTail0']

for i_dat, dat in enumerate(d_list):
    ax = fig5.add_subplot(spec5[i_dat+6,0])
    axes.append(ax)    
    ax.plot(dat,'k')
    
    ax.set_ylabel(names[i_dat]+"\ndist\n[m]")

#     ax.axhline(0,c='grey')       
    
    if i_dat  == 0:
        logic_raw = (self.d_nose2nose < .02) * (self.d_nose02ass > .06) * (self.d_nose12ass > .06) 
    if i_dat  == 1:
        logic_raw = (self.d_nose2nose > .06) * (self.d_nose02ass < .02) * (self.d_nose12ass > -1) 
    if i_dat  == 2:
        logic_raw = (self.d_nose2nose > .06) * (self.d_nose02ass > -1) * (self.d_nose12ass < .02) 
    
    logic_close=ndimage.binary_opening(logic_raw,structure = np.ones(3))
    logic_close=ndimage.binary_closing(logic_close,structure = np.ones(2*30))

    # frames is where it's true and the previous one is false
    logic_frames = np.argwhere(logic_close[:-1]*(~logic_close[1:]) ) + 1   
    d_frames.append(logic_frames.squeeze())
    
    
    llim,ulim = ax.get_ylim()
#     ax.plot(logic_close*ulim,c='r')
#     ax.plot(logic_raw,c='r')


#     ax.fill_between(np.arange(len(logic_close)),np.zeros_like(logic_close),logic_close*ulim,facecolor=colors_social[3],edgecolor='None')

    ax.imshow(0*logic_close[None,:], aspect="auto", cmap=cmap_social, vmin=0, vmax=len(colors)-1,  
              extent=(0, len(logic_close), llim, ulim))    
    cutoffs = [.01,.02,.02]   
    cut = cutoffs[i_dat]
    bottom = cut* np.ones_like(logic_close)
    bottom[logic_close == 1] = llim
#     ax.fill_between(np.arange(len(logic_close)),logic_close+cut,bottom,facecolor=colors_social[3],edgecolor=colors_social[3])
    ax.fill_between(np.arange(len(logic_close)),bottom,logic_close+cut,facecolor=colors_social[3],edgecolor='none')
    ax.axhline(cut,c='grey')
    ax.set_ylim([llim,ulim])

    
plt.subplots_adjust(hspace=0.05)

for ax in axes:
    pass
    ax.set_xlim([0,self.n_frames*1])
    if zoom:
        ax.set_xlim([64750,66000]) # ALSO good


for ax in axes[:-1]:
    adjust_spines(ax,'left')
adjust_spines(axes[-1],['left','bottom'])
plt.xlabel('Frame')


plt.savefig(savepath,transparent=True,dpi = 600)   
plt.show()
```

```
/home/chrelli/anaconda3/envs/myp/lib/python3.6/site-packages/ipykernel_launcher.py:83: MatplotlibDeprecationWarning: Adding an axes using the same arguments as a previous axes currently reuses the earlier instance.  In a future version, a new instance will always be created and returned.  Meanwhile, this warning can be suppressed, and the future behavior ensured, by passing a unique label to each axes instance.
/home/chrelli/anaconda3/envs/myp/lib/python3.6/site-packages/ipykernel_launcher.py:83: MatplotlibDeprecationWarning: Adding an axes using the same arguments as a previous axes currently reuses the earlier instance.  In a future version, a new instance will always be created and returned.  Meanwhile, this warning can be suppressed, and the future behavior ensured, by passing a unique label to each axes instance.
/home/chrelli/anaconda3/envs/myp/lib/python3.6/site-packages/ipykernel_launcher.py:83: MatplotlibDeprecationWarning: Adding an axes using the same arguments as a previous axes currently reuses the earlier instance.  In a future version, a new instance will always be created and returned.  Meanwhile, this warning can be suppressed, and the future behavior ensured, by passing a unique label to each axes instance.
/home/chrelli/anaconda3/envs/myp/lib/python3.6/site-packages/ipykernel_launcher.py:83: MatplotlibDeprecationWarning: Adding an axes using the same arguments as a previous axes currently reuses the earlier instance.  In a future version, a new instance will always be created and returned.  Meanwhile, this warning can be suppressed, and the future behavior ensured, by passing a unique label to each axes instance.
/home/chrelli/anaconda3/envs/myp/lib/python3.6/site-packages/ipykernel_launcher.py:128: UserWarning: This figure was using constrained_layout==True, but that is incompatible with subplots_adjust and or tight_layout: setting constrained_layout==False.
```

# And also plot the raw data¶

In [103]:

```
zoom = False
if zoom:
    fig5 = plt.figure(constrained_layout=True,figsize = (4,6))
    savepath = 'figs/pyro_tracked_data_zoom.png'

else:
    fig5 = plt.figure(constrained_layout=True,figsize = (18,6))
    savepath = 'figs/pyro_tracked_data.png'


heights = [1, 1, 1, 1,1]
n_subplots = len(heights)
spec5 = fig5.add_gridspec(ncols=1, nrows=n_subplots, height_ratios=heights)

axes = []


# plot XYZ for both mice
# make the 

for body_idx,sub in enumerate([0,1]):
    c_hip = self.body_points[body_idx][0]
    c_ass = self.body_points[body_idx][1]
    ax = fig5.add_subplot(spec5[sub,0])
    axes.append(ax)
    for i,name in enumerate(['x','y','z']):
        plt.plot(c_hip[:,i], c= self.cmpl[1+i],label=name)
    plt.ylabel(' \nMouse{}\nposition\n[m]'.format(body_idx))
#     if sub == 0:
#         plt.legend(loc='upper right')


# plot the running speed for both mice
# plot the z speed for both mice

for body_idx,sub in enumerate([2,3]):
    
    fwd,left,up = self.fwd[body_idx],self.left[body_idx],self.up[body_idx]
    ax = fig5.add_subplot(spec5[sub,0])
    axes.append(ax)
    plt.plot(fwd, c= self.cmpl[1],label='fwd')
    plt.plot(left, c= self.cmpl[2],label='left')
    plt.plot(up, c= self.cmpl[3],label='up')
    plt.ylabel('Mouse{}\nspeed\n[m/s]'.format(body_idx))
#     if sub == 2:
#         plt.legend(loc='upper right')    

        
# plot the social distances
labels = ["nose_0 <-> nose_1","nose_0 --> ass_1","nose_1 --> ass_0"]

labels = ["nose_0 <-> nose_1","nose_0 --> gen_1","nose_1 --> gen_0"]

d_list = [self.d_nose2nose,self.d_nose02ass,self.d_nose12ass]

sub = 4
ax = fig5.add_subplot(spec5[sub,0])
axes.append(ax)
for i in range(3):
    ax.plot(d_list[i],c=self.cmpl[i+1],label = labels[i])
    
# plt.legend(loc='upper right')    
plt.ylabel('Social\ndistance\n[m]')

        
# Fit arma model for running and rearing


# calculate the social distances

# Plot the 'Ethogram' for Running

# Plot the 'Ethogram' for Z-running (rearing)

# Plot the social ethogram (n2n,n2ass,n2ass)
# follwing is nose-ass, while running, for a while, for example. Mounting would be center above centers
# Select a few good examples for identified behaviors
plt.subplots_adjust(hspace=0.1)

for ax in axes:
    pass
    ax.set_xlim([0,self.n_frames*1])
    if zoom:
        ax.set_xlim([64750,66000])
        
for ax in axes[:-1]:
    adjust_spines(ax,'left')
adjust_spines(axes[-1],['left','bottom'])
plt.xlabel('Frame')


plt.savefig(savepath,transparent=True,dpi = 600)   
plt.show()
```

```
/home/chrelli/anaconda3/envs/myp/lib/python3.6/site-packages/ipykernel_launcher.py:82: UserWarning: This figure was using constrained_layout==True, but that is incompatible with subplots_adjust and or tight_layout: setting constrained_layout==False.
```
