# Supplementary material for: Automatic mapping of multiplexed social receptive fields by deep learning and GPU-accelerated 3D videography
Source: Nat Commun. 2022 Feb 1;13:593. doi: 10.1038/s41467-022-28153-7 (PMC8807631; doi:10.1038/s41467-022-28153-7)
Supplement: Supplementary file 9 — Supplementary Software [file 41467_2022_28153_MOESM9_ESM.zip › ebbesen_froemke_2021_code/analysis/014_Make_Kalman_filtering_video.html]

014\_Make\_Kalman\_filtering\_video


In [1]:

```
# IDEA: Add neck to the posture map?
from IPython import get_ipython

# QT for movable plots
%load_ext autoreload
%autoreload 2

import time, os, sys, shutil
from utils.fitting_utils import *

# for math and plotting
import pandas as pd
import numpy as np
import scipy as sp
import matplotlib.pyplot as plt
# %matplotlib notebook
# %matplotlib inline

# %matplotlib widget
%matplotlib qt

from itertools import compress # for list selection with logical
from tqdm import tqdm

from multiprocessing import Process

# ALLSO JIT STUFF
from numba import jit, njit

# and pytorch
import torch

import sys, os, pickle
# import cv2
from colour import Color
import h5py
from tqdm import tqdm, tqdm_notebook
import glob
import itertools
```

In [2]:

```
from utils.analysis_tools import adjust_spines,cmpl
```

# Load tracked data and plot a frame¶

In [3]:

```
#load the tracked data!
data_folder = 'example_data/tracking/'

with open(data_folder +'tracked_behavior.pkl', 'rb') as f:
    tracked_behavior = pickle.load(f)
print(tracked_behavior.keys())

# load ALL the frames as jagged lines 
with h5py.File(data_folder+'/pre_processed_frames.hdf5', mode='r') as hdf5_file:
    print(hdf5_file.keys())
    print(len(hdf5_file['dataset']))
    jagged_lines = hdf5_file['dataset'][...]
```

```
dict_keys(['var', 'ivar', 'body_constants', 'start_frame', 'end_frame', 'tracking_holder', 'guessing_holder', 'data_folder'])
<KeysViewHDF5 ['dataset']>
74962
```

In [4]:

```
from utils.cuda_tracking_utils import unpack_from_jagged, cheap4d
# kill first 6 secs of the frames (delay is ~180)
start_frame = 30*60
pos, pos_weights, keyp, pkeyp, ikeyp = unpack_from_jagged(jagged_lines[start_frame])
print(ikeyp)
print(pos.shape)

cheap4d(pos,keyp,ikeyp)
# fig = plt.gcf()
# plt.title("N positions is {}".format(pos.shape))
```

```
[0 1 1 2 3 0 1 1 1 2 2 0 2 0 1 1 1 2 3]
(2986, 3)
```

```
findfont: Font family ['cursive'] not found. Falling back to DejaVu Sans.
```

In [5]:

```
print(tracked_behavior['tracking_holder'].shape)
print(tracked_behavior['var'])
print(tracked_behavior['ivar'])
print(tracked_behavior.keys())
```

```
(18, 73361)
['b', 'c', 's', 'psi', 'theta', 'phi', 'x', 'y', 'z', 'b', 'c', 's', 'theta', 'phi', 'x', 'y', 'z']
['b0', 'c0', 's0', 'psi0', 'theta0', 'phi0', 'x0', 'y0', 'z0', 'b1', 'c1', 's1', 'theta1', 'phi1', 'x1', 'y1', 'z1']
dict_keys(['var', 'ivar', 'body_constants', 'start_frame', 'end_frame', 'tracking_holder', 'guessing_holder', 'data_folder'])
```

# Plot a raw frame, check that the plotter works¶

In [6]:

```
from utils.analysis_tools import VideoPlotMachine,PlotMachine
# take a list of frames, calculate body supports and plot as a kind of decaying trail, with some decay and lengthm maybe the center of the nose??
plt.close('all')
Plotter = PlotMachine(tracked_behavior,jagged_lines)
#Plotter.kernel_smoothing(9)

example_frame = 11800

n_fine = 5
Plotter.make(example_frame,view_override = [55.0,90.])
```

```
11800
10200
i_trace_start is 9240 and i_frame is 10200
['nose']
['nose']
```

# Here we copy the smoothing functions, so they are easy to make plots from¶

In [ ]:

```
sigma_process = .01
sigma_measure = .015            
            
from filterpy.common import kinematic_kf,Q_discrete_white_noise
from filterpy.kalman import FixedLagSmoother

def kalman1_3D(tr,sigma_process,sigma_measure,dt = 1/60):
    # make first order kinematic kalman filter
    cv = kinematic_kf(dim=3, order=1, dt = dt) 
    cv.R = np.eye(3) * sigma_measure**2
    G = np.array([[0.5 * dt**2, dt]], np.float32).T
    Q0 = np.matmul(G, G.T) * sigma_process**2

    for i in [0,2,4]:
        cv.Q[i:(i+2),i:(i+2)] = Q_discrete_white_noise(dim=2, dt=dt, var=sigma_process**2)
    #     cv.Q[i:(i+2),i:(i+2)] = Q0
    cv.P = np.ones((cv.dim_x,cv.dim_x))*0.001 +.0001
    
    kalman_estimate = []
    # initialize
    cv.x =  np.array([[ tr[0,0],0,tr[0,1],0,tr[0,2] ,0 ]]).T
    cv.update(tr[i,:])
    for i in tqdm(range(tr.shape[0])):
        cv.predict()
        cv.update(tr[i,:][:,np.newaxis])

        kalman_estimate.append(cv.x)

    kalman_estimate = np.hstack(kalman_estimate)    
    tr_filtered = kalman_estimate[[0,2,4],:].T
    return tr_filtered

def fls1_3d(tr,sigma_process,sigma_measure,dt = 1/60,N_lag = 16):
    # make first order kinematic kalman filter
    cv = kinematic_kf(dim=3, order=1, dt = dt) 
    cv.R = np.eye(3) * sigma_measure**2
    G = np.array([[0.5 * dt**2, dt]], np.float32).T
    Q0 = np.matmul(G, G.T) * sigma_process**2

    for i in [0,2,4]:
        cv.Q[i:(i+2),i:(i+2)] = Q_discrete_white_noise(dim=2, dt=dt, var=sigma_process**2)
    #     cv.Q[i:(i+2),i:(i+2)] = Q0
    cv.P = np.ones((cv.dim_x,cv.dim_x))*0.001 +.0001
    
    kalman_estimate = []
    # initialize
    cv.x =  np.array([[ tr[0,0],0,tr[0,1],0,tr[0,2] ,0 ]]).T
    
    # also make an FLS smoother 
    fls = FixedLagSmoother(dim_x=6, dim_z=3, N=N_lag)

    fls.x = np.copy(cv.x)
    fls.F = np.copy(cv.F)
    fls.H = np.copy(cv.H)
    fls.P = np.copy(cv.P)
    fls.R = np.copy(cv.R)
    fls.Q = np.copy(cv.Q)
    
    for i in tqdm(range(tr.shape[0])):
        cv.predict()
        cv.update(tr[i,:][:,np.newaxis])
        fls.smooth(tr[i,:][:,np.newaxis])
        kalman_estimate.append(cv.x)

    kalman_estimate = np.hstack(kalman_estimate)    
    fls_estimate = np.hstack(fls.xSmooth)
    tr_filtered = kalman_estimate[[0,2,4],:].T
    tr_smoothed = fls_estimate[[0,2,4],:].T
    return tr_smoothed

def fls2_3d(tr,sigma_process,sigma_measure,dt = 1/60,N_lag = 16):
    # make second order kinematic kalman filter
    cv = kinematic_kf(dim=3, order=2, dt = dt) 
    cv.R = np.eye(3) * sigma_measure**2
    G = np.array([[0.5 * dt**2, dt]], np.float32).T
    Q0 = np.matmul(G, G.T) * sigma_process**2

    for i in [0,3,6]:
        cv.Q[i:(i+3),i:(i+3)] = Q_discrete_white_noise(dim=3, dt=dt, var=sigma_process**2)
    #     cv.Q[i:(i+2),i:(i+2)] = Q0
    cv.P = np.ones((cv.dim_x,cv.dim_x))*0.001 +.0001
    
    kalman_estimate = []
    # initialize
    cv.x =  np.array([[ tr[0,0],0,0,tr[0,1],0,0,tr[0,2],0 ,0 ]]).T

        
    # also make an FLS smoother 
    fls = FixedLagSmoother(dim_x=9, dim_z=3, N=N_lag)

    fls.x = np.copy(cv.x)
    fls.F = np.copy(cv.F)
    fls.H = np.copy(cv.H)
    fls.P = np.copy(cv.P)
    fls.R = np.copy(cv.R)
    fls.Q = np.copy(cv.Q)
    
    for i in tqdm(range(tr.shape[0])):
        cv.predict()
        cv.update(tr[i,:][:,np.newaxis])
        fls.smooth(tr[i,:][:,np.newaxis])

        kalman_estimate.append(cv.x)

    kalman_estimate = np.hstack(kalman_estimate)    
    fls_estimate = np.hstack(fls.xSmooth)
    tr_filtered = kalman_estimate[[0,3,6],:].T
    tr_smoothed = fls_estimate[[0,3,6],:].T
    return tr_smoothed

def kalman_1D(tr,sigma_process,sigma_measure,dt = 1/60):
    # make first order kinematic kalman filter
    cv = kinematic_kf(dim=1, order=1, dt = dt) 
    cv.R = np.eye(1) * sigma_measure**2
    G = np.array([[0.5 * dt**2, dt]], np.float32).T
    Q0 = np.matmul(G, G.T) * sigma_process**2

    for i in [0]:
        cv.Q[i:(i+2),i:(i+2)] = Q_discrete_white_noise(dim=2, dt=dt, var=sigma_process**2)
    #     cv.Q[i:(i+2),i:(i+2)] = Q0
    cv.P = np.ones((cv.dim_x,cv.dim_x))*0.001 +.0001

    kalman_estimate = []
    # initialize
    cv.x =  np.array([[ tr[0],0 ]]).T
    cv.update(tr[i])
    for i in tqdm(range(tr.shape[0])):
        cv.predict()
        cv.update(tr[i])

        kalman_estimate.append(cv.x)

    kalman_estimate = np.hstack(kalman_estimate)    
    tr_filtered = kalman_estimate[[0],:].T
    return tr_filtered

def kalman2_3D(tr,sigma_process,sigma_measure,dt = 1/60):
    # make second order kinematic kalman filter
    cv = kinematic_kf(dim=3, order=2, dt = dt) 
    cv.R = np.eye(3) * sigma_measure**2
    G = np.array([[0.5 * dt**2, dt]], np.float32).T
    Q0 = np.matmul(G, G.T) * sigma_process**2

    for i in [0,3,6]:
        cv.Q[i:(i+3),i:(i+3)] = Q_discrete_white_noise(dim=3, dt=dt, var=sigma_process**2)
    #     cv.Q[i:(i+2),i:(i+2)] = Q0
    cv.P = np.ones((cv.dim_x,cv.dim_x))*0.001 +.0001
    
    kalman_estimate = []
    # initialize
    cv.x =  np.array([[ tr[0,0],0,0,tr[0,1],0,0,tr[0,2],0 ,0 ]]).T
    cv.update(tr[i,:])
    for i in tqdm(range(tr.shape[0])):
        cv.predict()
        cv.update(tr[i,:][:,np.newaxis])

        kalman_estimate.append(cv.x)

    kalman_estimate = np.hstack(kalman_estimate)    
    tr_filtered = kalman_estimate[[0,3,6],:].T
    return tr_filtered

def fls2_1d(tr,sigma_process,sigma_measure,dt = 1/60,N_lag = 16):
    # make second order kinematic kalman filter
    cv = kinematic_kf(dim=1, order=2, dt = dt) 
    cv.R = np.eye(1) * sigma_measure**2
    G = np.array([[0.5 * dt**2, dt]], np.float32).T
    Q0 = np.matmul(G, G.T) * sigma_process**2

    for i in [0]:
        cv.Q[i:(i+2),i:(i+2)] = Q_discrete_white_noise(dim=2, dt=dt, var=sigma_process**2)
    #     cv.Q[i:(i+2),i:(i+2)] = Q0
    cv.P = np.ones((cv.dim_x,cv.dim_x))*0.001 +.0001
    
    kalman_estimate = []
    # initialize
    cv.x =  np.array([[ tr[0],0,0]]).T

        
    # also make an FLS smoother 
    fls = FixedLagSmoother(dim_x=3, dim_z=1, N=N_lag)

    fls.x = np.copy(cv.x)
    fls.F = np.copy(cv.F)
    fls.H = np.copy(cv.H)
    fls.P = np.copy(cv.P)
    fls.R = np.copy(cv.R)
    fls.Q = np.copy(cv.Q)
    
#     print(cv)
#     print(fls)
    
    for i in tqdm(range(tr.shape[0])):
        cv.predict()
        cv.update(tr[i])
        fls.smooth(tr[i])

        kalman_estimate.append(cv.x)

    kalman_estimate = np.hstack(kalman_estimate)    
    fls_estimate = np.hstack(fls.xSmooth)
    tr_filtered = kalman_estimate[[0],:].T
    tr_smoothed = fls_estimate[[0],:].T
    return tr_smoothed

def fls1_1d(tr,sigma_process,sigma_measure,dt = 1/60,N_lag = 16):
    # make second order kinematic kalman filter
    cv = kinematic_kf(dim=1, order=1, dt = dt) 
    cv.R = np.eye(1) * sigma_measure**2
    G = np.array([[0.5 * dt**2, dt]], np.float32).T
    Q0 = np.matmul(G, G.T) * sigma_process**2

    for i in [0]:
        cv.Q[i:(i+2),i:(i+2)] = Q_discrete_white_noise(dim=2, dt=dt, var=sigma_process**2)
    #     cv.Q[i:(i+2),i:(i+2)] = Q0
    cv.P = np.ones((cv.dim_x,cv.dim_x))*0.001 +.0001
    
    kalman_estimate = []
    # initialize
    cv.x =  np.array([[ tr[0],0]]).T

    
    # also make an FLS smoother 
    fls = FixedLagSmoother(dim_x=2, dim_z=1, N=N_lag)

    fls.x = np.copy(cv.x)
    fls.F = np.copy(cv.F)
    fls.H = np.copy(cv.H)
    fls.P = np.copy(cv.P)
    fls.R = np.copy(cv.R)
    fls.Q = np.copy(cv.Q)
    
    fls.x =  np.array([[ .9,0]]).T
    fls.P = np.array([[2.73445008e-04 ,2.49619926e-05],[2.49619926e-05, 4.56088374e-06]])
    
    for i in tqdm(range(tr.shape[0])):
        cv.predict()
        cv.update(tr[i])
        fls.smooth(tr[i])
        kalman_estimate.append(cv.x)
        
    kalman_estimate = np.hstack(kalman_estimate)    
    fls_estimate = np.hstack(fls.xSmooth)
    tr_filtered = kalman_estimate[[0],:].T
    tr_smoothed = fls_estimate[[0],:].T
    return tr_smoothed
```

# Smooth the 3D skeleton data¶

In [8]:

```
# function for filtering and error correction!
from utils.analysis_tools import particles_to_body_supports_cuda,VideoPlotMachine

# overwrite HACK for now
Plotter = VideoPlotMachine(tracked_behavior,jagged_lines)

self = Plotter 
# get the raw tracking data!
part = self.tracking_holder

# unpack all the 3D coordinates!
part = torch.from_numpy(part).float().cuda()
part = torch.transpose(part,0,1)
body_support_0 = particles_to_body_supports_cuda(part[:,:9],implant = True)
body_support_1 = particles_to_body_supports_cuda(part[:,9:],implant = False)
# and the spine length
s_0 = part[:,2].cpu().numpy()
s_1 = part[:,2+9].cpu().numpy()

# and smooth the data
from utils.analysis_tools import smooth_body_support

body_support_0_smooth,s_0_smooth = smooth_body_support(body_support_0,s_0)
body_support_1_smooth,s_1_smooth = smooth_body_support(body_support_1,s_1)
```

```
100%|██████████| 73361/73361 [00:20<00:00, 3599.32it/s]
100%|██████████| 73361/73361 [00:20<00:00, 3652.96it/s]
100%|██████████| 73361/73361 [00:20<00:00, 3651.48it/s]
100%|██████████| 73361/73361 [00:20<00:00, 3634.43it/s]
100%|██████████| 73361/73361 [00:20<00:00, 3651.39it/s]
100%|██████████| 73361/73361 [00:19<00:00, 3689.13it/s]
100%|██████████| 73361/73361 [00:10<00:00, 7011.24it/s]
100%|██████████| 73361/73361 [00:19<00:00, 3674.47it/s]
100%|██████████| 73361/73361 [00:19<00:00, 3669.03it/s]
100%|██████████| 73361/73361 [00:19<00:00, 3671.91it/s]
100%|██████████| 73361/73361 [00:19<00:00, 3668.28it/s]
100%|██████████| 73361/73361 [00:20<00:00, 3642.19it/s]
100%|██████████| 73361/73361 [00:19<00:00, 3674.98it/s]
100%|██████████| 73361/73361 [00:10<00:00, 7057.19it/s]
```

# And smooth the rotation matrices¶

In [9]:

```
# calculate rotation matrices from the smoothed skeleton points, to be used for smooth video playback

def rotation_matrix_vec2vec_numpy(f,t):
    # from this paper, ffrom math stacj
    # but made batch-able for pytorch
    # https://math.stackexchange.com/questions/180418/calculate-rotation-matrix-to-align-vector-a-to-vector-b-in-3d/476311#476311
    #rotate vector f onto vector t
    # import numpy as np
    # v = np.cross(f, t)
    # u = v/np.linalg.norm(v)
    # c = np.dot(f, t)
    # h = (1 - c)/(1 - c**2)

    # vx, vy, vz = v
    # rot =[[c + h*vx**2, h*vx*vy - vz, h*vx*vz + vy],
    #       [h*vx*vy+vz, c+h*vy**2, h*vy*vz-vx],
    #       [h*vx*vz - vy, h*vy*vz + vx, c+h*vz**2]]

    # good disussion about smoothing rotation matrices later: https://www.cvl.isy.liu.se/education/graduate/geometry2010/lectures/Lecture7b.pdf
    # rotate f onto t
    # very fast, but slightly numerically unstable, so we add epsilon!


    epsilon = 1e-6
    # f = x_pointer
    # t = nose_pointer
    # cross product
    v = np.cross(f,t)
    u = v/(np.linalg.norm(v,axis=1)[:,np.newaxis] + epsilon)
    # dot product
    c = np.einsum('i,ai->a', f,t)
    # the factor h
    h = (1 - c)/(1 - c**2 + epsilon)

    vx, vy, vz = v[:,0],v[:,1],v[:,2]

    R = np.stack([np.stack([c + h*vx**2, h*vx*vy - vz, h*vx*vz + vy], axis=1),
                             np.stack([h*vx*vy+vz, c+h*vy**2, h*vy*vz-vx],      axis=1),
                             np.stack([h*vx*vz - vy, h*vy*vz + vx, c+h*vz**2], axis=1)], axis=1)

    return R

# use quarterneons to smooth the body ellipsoid rotations
# convert the rotation matrices to quarternions
from pyquaternion import Quaternion

def unpack_axis_angels(R):
    # calculate the axis-angle representation
    # https://stackoverflow.com/questions/15022630/how-to-calculate-the-angle-from-rotation-matrix
    angle_x = np.arctan2(R[:,2,1],R[:,2,2])
    angle_y = np.arctan2(-R[:,2,0],np.sqrt(R[:,2,1]**2 + R[:,2,2]**2  ) )
    angle_z = np.arctan2(R[:,1,0],R[:,0,0])
    return np.stack((angle_x,angle_y,angle_z),axis = 1)

def averageQuaternions(Q):
    # from https://github.com/christophhagen/averaging-quaternions/blob/master/averageQuaternions.py
    # Number of quaternions to average
    M = Q.shape[0]
    A = np.zeros(shape=(4,4))

    for i in range(0,M):
        q = Q[i,:]
        # multiply q with its transposed version q' and add A
        A = np.outer(q,q) + A

    # scale
    A = (1.0/M)*A
    # compute eigenvalues and -vectors
    eigenValues, eigenVectors = np.linalg.eig(A)
    # Sort by largest eigenvalue
    eigenVectors = eigenVectors[:,eigenValues.argsort()[::-1]]
    # return the real part of the largest eigenvector (has only real part)
    return np.real(eigenVectors[:,0].ravel())    

def quaternion_smoothing(R):
    angles_kalman = unpack_axis_angels(R)     
    q_x = [ Quaternion(axis = (1.,0.,0.),radians = r) for r in angles_kalman[:,0] ]
    q_y = [ Quaternion(axis = (0.,1.,0.),radians = r) for r in angles_kalman[:,1] ]
    q_z = [ Quaternion(axis = (0.,0.,1.),radians = r) for r in angles_kalman[:,2] ]
    # now, smooth the rotations
    q_all = []
    for i in range(len(q_x)):
        q_all.append(q_x[i]*q_y[i]*q_z[i])

    # convert to a matrix w qith w x y z    
    Q = np.stack([q.elements for q in q_all],axis = 0)

    # try a running average first!
    Q_run_av = np.copy(Q)
    w_length = 9 # must be uneven
    h_length = int(np.floor(w_length/2))
    for i in tqdm(np.arange(np.floor(w_length/2),Q.shape[0]-np.ceil(w_length/2))):
        i = int(i) 
        Q_run_av[i,:] = averageQuaternions(Q[(i-h_length):(i+h_length+1),:])


    # convert back to rotation matrices, to check that converion is fine
    R_q_list = [q.rotation_matrix for q in q_all]
    R_q = np.stack(R_q_list,axis = 0)

    R_q_smooth_list = [Quaternion(Q_run_av[i,:]).rotation_matrix for i in range(Q_run_av.shape[0])]
    R_q_smooth = np.stack(R_q_smooth_list,axis = 0)
    return R_q,R_q_smooth

def smooth_rotation_matrices(body_support_0_smooth):
    # first calculate body vectors from the smoothed skeleton points
    c_hip,c_ass,c_mid,c_nose,c_tip,c_impl,R_body,R_head,R_nose = body_support_0_smooth

    #todo, maybe average across the both noisy estimates here, prob won't gain much though..
    v_nose = c_nose - c_mid
    v_hip = c_mid - c_hip

    v_nose = v_nose/np.linalg.norm(v_nose,axis=1)[:,np.newaxis]
    v_hip = v_hip/np.linalg.norm(v_hip,axis=1)[:,np.newaxis]


    # To calculate R_nose, we ask how we have to rotate a vector along x, so that it points along the hip or nose
    f = np.array([1,0,0])
    t = v_nose
    R_nose_smooth = rotation_matrix_vec2vec_numpy(f,t)

    t = v_hip
    R_body_smooth = rotation_matrix_vec2vec_numpy(f,t)
    return [c_hip,c_ass,c_mid,c_nose,c_tip,c_impl,R_body_smooth,R_head,R_nose_smooth]

def smooth_rotation_matrices_quaternion(body_support_0_smooth):
    # first calculate body vectors from the smoothed skeleton points
    c_hip,c_ass,c_mid,c_nose,c_tip,c_impl,R_body,R_head,R_nose = body_support_0_smooth

    #todo, maybe average across the both noisy estimates here, prob won't gain much though..
    v_nose = c_nose - c_mid
    v_hip = c_mid - c_hip

    v_nose = v_nose/np.linalg.norm(v_nose,axis=1)[:,np.newaxis]
    v_hip = v_hip/np.linalg.norm(v_hip,axis=1)[:,np.newaxis]


    # To calculate R_nose, we ask how we have to rotate a vector along x, so that it points along the hip or nose
    f = np.array([1,0,0])
    t = v_nose
    R_nose_smooth = rotation_matrix_vec2vec_numpy(f,t)

    t = v_hip
    R_body_smooth = rotation_matrix_vec2vec_numpy(f,t)
    
    R_q,R_body_smooth = quaternion_smoothing(R_body_smooth)
    R_q,R_nose_smooth = quaternion_smoothing(R_nose_smooth)
    
    return [c_hip,c_ass,c_mid,c_nose,c_tip,c_impl,R_body_smooth,R_head,R_nose_smooth]
```

In [11]:

```
# Make example plots for smoothing!
# and add the smoothed and raw data to the video maker
from utils.analysis_tools import particles_to_body_supports_cuda,VideoPlotMachine

Plotter = VideoPlotMachine(tracked_behavior,jagged_lines)
self = Plotter

# add the smoothed coordinates as numpy arrays
self.body_support_0_raw = [i.cpu().numpy().squeeze() for i in body_support_0]
self.body_support_0_smooth = body_support_0_smooth
self.s_0_raw = s_0
self.s_0_smooth = s_0_smooth
self.body_support_1_raw = [i.cpu().numpy().squeeze() for i in body_support_1]
self.body_support_1_smooth = body_support_1_smooth
self.s_1_raw = s_1
self.s_1_smooth = s_1_smooth
# also smooth the body ellipsoid rotations
self.body_support_0_smooth = smooth_rotation_matrices(body_support_0_smooth)
self.body_support_1_smooth = smooth_rotation_matrices(body_support_1_smooth)

# self.body_support_0_smooth = smooth_rotation_matrices_quaternion(body_support_0_smooth)
# self.body_support_1_smooth = smooth_rotation_matrices_quaternion(body_support_1_smooth)
```

# Make example videos for smoothing¶

In [15]:

```
# Example stills for the figure!
example_frame = 34000
self.make_me(example_frame,cloud = True,skel='smooth',ellip='smooth',
             trace='smooth',view_override = [47,74],savepath='figs/example_smooth.pdf') #47.74, 25.86

self.make_me(example_frame,cloud = True,skel='raw',ellip='raw',
             trace='raw',view_override = [47,74],savepath='figs/example_raw.pdf') #47.74, 25.86
```

```
c_hip is [-0.03932851  0.01097639  0.02462598]
c_hip is [ 0.08866866 -0.07113001  0.0228825 ]
34000
32400
i_trace_start is 31800 and i_frame is 32400
['nose']
['nose']
```

```
c_hip is [-0.04035885  0.01092708  0.0248792 ]
c_hip is [ 0.09418259 -0.06534421  0.02760802]
34000
32400
i_trace_start is 31800 and i_frame is 32400
['nose']
['nose']
```

In [14]:

```
# Example videos for smoothing
self.video_me(frame_list=34000+np.arange(-120,60)*30, savepath = 'videos/example_raw.mp4',
              trace='raw',skel='raw',ellip='raw',view_override = [47,74],fps = 10)

self.video_me(frame_list=34000+np.arange(-120,60)*30, savepath = 'videos/example_smooth.mp4',
              trace='smooth',skel='smooth',ellip='smooth',view_override = [47,74],fps=10)
plt.close('all')
```

```
c_hip is [-0.04881905  0.00620681  0.02576634]
c_hip is [ 0.15365421 -0.05854517  0.02130347]
30400
28800
i_trace_start is 28200 and i_frame is 28800
['nose']
['nose']
```

In [86]:

```
# merge the videos using opencv, from here: https://gist.github.com/nkint/8576156
import cv2
import os

# this two lines are for loading the videos.
# in this case the video are named as: cut1.mp4, cut2.mp4, ..., cut15.mp4
# videofiles = [n for n in os.listdir('.') if n[0]=='c' and n[-4:]=='.mp4']
# videofiles = sorted(videofiles, key=lambda item: int( item.partition('.')[0][3:]))

video_files = ['videos/example_raw.mp4', 'videos/example_smooth.mp4']


cap_0 = cv2.VideoCapture(video_files[0])
cap_1 = cv2.VideoCapture(video_files[1])

                                    
                                    
# video resolution: 1624x1234 px
# out = cv2.VideoWriter("video.avi", 
#                       cv2.cv.CV_FOURCC('F','M','P', '4'), 
#                       15, (1624, 1234), 1)
# get vcap property 
width  = cap_0.get(cv2.CAP_PROP_FRAME_WIDTH)   # float
height = cap_0.get(cv2.CAP_PROP_FRAME_HEIGHT)  # float

fourcc = cv2.VideoWriter_fourcc(*'MP4V')
out = cv2.VideoWriter('videos/supplementary_video_smoothing.mp4', fourcc, 10, (int(2*width), int(height)))
# fourcc = cv2.VideoWriter_fourcc(*'XVID')
# out = cv2.VideoWriter('videos/smoothing.avi', fourcc, 20, (int(2*width), int(height)))


# fourcc = cv2.VideoWriter_fourcc(*'XVID')
# out = cv2.VideoWriter('cutout.avi', fourcc, 20.0, (640, 480))

# create a splash screen
# create blank image
img = np.zeros((int(height),int(2*width), 3), np.uint8)
font = cv2.FONT_HERSHEY_SIMPLEX
font_color = (255, 255, 255)
h = height-.1*height
w = width
font_scale = 1
thickness = 2
text = 'Ebbesen & Froemke, 2020'
def put_centered_text(img,text,w,h,font, font_scale, font_color, thickness):
    # get boundary of this text
    textsize = cv2.getTextSize(text, font, font_scale, thickness)[0]
    cv2.putText(img, text, (int(w - textsize[0]/2),int(h) ), font, font_scale, font_color, thickness, cv2.LINE_AA)

put_centered_text(img,text,w,h,font, font_scale, font_color, thickness)
put_centered_text(img,'Supplementary video 3: State-space filtering',w,.2*height,font, font_scale, font_color, thickness)

for _ in range(30):
    cv2.imshow('frame',img)
    cv2.waitKey(10)
    out.write(img)
    


while(cap_0.isOpened() | cap_1.isOpened()):
    
    
    ret, frame_0 = cap_0.read()
    ret, frame_1 = cap_1.read()
    if frame_1 is not None:
        stacked_frame = cv2.hconcat([frame_0,frame_1])
        
        # Add annotation!
        # centering is not easy, in cv2: https://gist.github.com/xcsrz/8938a5d4a47976c745407fe2788c813a
        font = cv2.FONT_HERSHEY_SIMPLEX
        font_color = (0,0,0)
        h = height-.9*height
        w = width/2
        font_scale = 1
        thickness = 2
        # get boundary of this text
        put_centered_text(stacked_frame,'Raw frame-by-frame data',w,h,font, font_scale, font_color, thickness)
        put_centered_text(stacked_frame,'After state-space filtering',w+width,h,font, font_scale, font_color, thickness)

        cv2.imshow('frame',stacked_frame)
        out.write(stacked_frame)
    else:
        break

cap_0.release()
cap_1.release()
out.release()
cv2.destroyAllWindows()

print ("end.")
```

```
end.
```

In [1]:

```
# Also make a gif for the website
```

In [14]:

```
# Example videos for smoothing
self.video_me(frame_list=34000+np.arange(-120,60)*30, savepath = 'videos/example_tracking_25.gif',
              trace='smooth',skel='smooth',ellip='smooth',view_override = [47,74],fps=10,dpi = 25)
plt.close('all')
```

In [29]:

```
# crop the gif a little bit
import imageio
import numpy as np    

#Create reader object for the gif
path_list = ['videos/example_tracking_30.gif']
gifs = [imageio.get_reader(f) for f in path_list]

number_of_frames = min([g.get_length() for g in gifs]) 

#Create writer object
now = time.time()
new_gif = imageio.get_writer('videos/example_tracking_wide_30b.gif',fps =10)


for frame_number in tqdm(range(number_of_frames)):
    imgs = [g.get_next_data() for g in gifs]
    new_image = np.hstack(imgs) 
    new_gif.append_data(new_image[24:-24,:,:])

for g in gifs:
    g.close()
new_gif.close()
```

```
100%|██████████| 179/179 [00:01<00:00, 111.78it/s]
```

# Make plots for a supplementary figure showing how we do the state-space filtering¶

In [21]:

```
# Make example plots for smoothing!
# and add the smoothed and raw data to the video maker
from utils.analysis_tools import particles_to_body_supports_cuda,VideoPlotMachine

Plotter = VideoPlotMachine(tracked_behavior,jagged_lines)
self = Plotter

# add the smoothed coordinates as numpy arrays
self.body_support_0_raw = [i.cpu().numpy().squeeze() for i in body_support_0]
self.body_support_0_smooth = body_support_0_smooth
self.s_0_raw = s_0
self.s_0_smooth = s_0_smooth
self.body_support_1_raw = [i.cpu().numpy().squeeze() for i in body_support_1]
self.body_support_1_smooth = body_support_1_smooth
self.s_1_raw = s_1
self.s_1_smooth = s_1_smooth
# also smooth the body ellipsoid rotations
self.body_support_0_smooth = smooth_rotation_matrices(body_support_0_smooth)
self.body_support_1_smooth = smooth_rotation_matrices(body_support_1_smooth)

# c_hip,c_ass,c_mid,c_nose,c_tip,c_impl,R_body,R_head,R_nose = self.body_support_0_raw
# angles_raw = unpack_axis_angels(R_body)

# c_hip,c_ass,c_mid,c_nose,c_tip,c_impl,R_body,R_head,R_nose = self.body_support_0_smooth
# angles_kalman = unpack_axis_angels(R_body)

# R_q,R_q_smooth = quaternion_smoothing(R_body)
# angles_q = unpack_axis_angels(R_q)
# angles_q_smooth = unpack_axis_angels(R_q_smooth)

c_hip,c_ass,c_mid,c_nose,c_tip,c_impl,R_body,R_head,R_nose = self.body_support_0_raw
angles_raw = unpack_axis_angels(R_nose)

c_hip,c_ass,c_mid,c_nose,c_tip,c_impl,R_body,R_head,R_nose = self.body_support_0_smooth
angles_kalman = unpack_axis_angels(R_nose)

R_q,R_q_smooth = quaternion_smoothing(R_nose)
angles_q = unpack_axis_angels(R_q)
angles_q_smooth = unpack_axis_angels(R_q_smooth)
```

```
100%|██████████| 73352/73352 [00:05<00:00, 14366.76it/s]
```

In [22]:

```
import matplotlib
%matplotlib inline

# Say, "the default sans-serif font is COMIC SANS"
matplotlib.rcParams['font.sans-serif'] = "Liberation Sans"
# Then, "ALWAYS use sans-serif fonts"
matplotlib.rcParams['font.family'] = "sans-serif"

matplotlib.rc('font', family='sans-serif') 
matplotlib.rc('text', usetex='false') 
matplotlib.rcParams.update({'font.size': 13})

from palettable.cmocean.sequential import Algae_6
cmpl = Algae_6.mpl_colors


sc=2
plt.figure(figsize = (4,5) )
n_vars = len(tracked_behavior['var'])
n_frames = tracked_behavior['tracking_holder'].shape[1]
start_frame = 30000
frame_window = 2400

scale_bar = [1,1,.1,1,1,1,.01,.01,.01,1,1,.1,1,1,.1,.1,.1]
units = ["rad","rad","a.u.",'rad','rad','rad',"m","m","m","rad","rad","a.u.",'rad','rad',"m","m","m"]
latex_vars = ['β', 'γ', 's', 'ψ', 'θ', 'φ', 'x', 'y', 'z', 'β', 'γ', 's', 'θ', 'φ', 'x', 'y', 'z']

for i in range(n_vars):
    plt.subplot(n_vars,1,1+i)
    
    plt.plot(tracked_behavior['tracking_holder'][i,start_frame:(start_frame+frame_window)],c=cmpl[1])
    plt.plot(tracked_behavior['guessing_holder'][i,start_frame:(start_frame+frame_window)],c=cmpl[3])
    
    ax = plt.gca()
    plt.gca().spines['top'].set_visible(False)
    plt.gca().spines['right'].set_visible(False)
    plt.gca().spines['left'].set_visible(False)
    
    if i < (n_vars-1):

        pass
        plt.gca().spines['bottom'].set_visible(False)
        plt.xticks([])

#         adjust_spines(ax,[])
    else:
        pass
#         adjust_spines(ax,['bottom'])

        plt.gca().spines['bottom'].set_bounds(0,600)
        plt.xticks([0,600])
        
    if i < 9:
        ax.set_facecolor('k')
        ax.patch.set_alpha(0.13)
    else:
        ax.set_facecolor('peru')      
        ax.patch.set_alpha(0.2)

        
#         adjust_spines(ax,['bottom','left'])
    plt.yticks([])
#     plt.ylabel(tracked_behavior['var'][i])
#     plt.ylabel(latex_vars[i])
    plt.xlim([0,frame_window+20])
    
    ax = plt.gca()
    
    ylim=ax.get_ylim()
    xlim=ax.get_xlim()
    plt.plot( -2+np.array([1,1])*xlim[1],ylim[0]+np.array([0,1])*scale_bar[i],'-k' )
#     plt.text(xlim[1],ylim[0]," "+str(scale_bar[i])+' '+units[i] )
    
plt.subplots_adjust(left=None, bottom=None, right=None, top=None, wspace=None, hspace=0.)
# plt.xlabel('Frames',labelpad =-10)        
# plt.tight_layout()
if False:
    plt.savefig('/home/chrelli/git/3d_sandbox/mouseposev0p2/figure_raw_pics'+
            '/supplementary_body_points/raw_tracked.png',transparent=False,
            bbox_inches = 'tight',pad_inches =1)  

plt.show()
```

In [23]:

```
def unlink_wrap(dat, lims=[-np.pi, np.pi], thresh = 0.95):
    # nice trick: https://stackoverflow.com/questions/27138751/preventing-plot-joining-when-values-wrap-in-matplotlib-plots
    """
    Iterate over contiguous regions of `dat` (i.e. where it does not
    jump from near one limit to the other).

    This function returns an iterator object that yields slice
    objects, which index the contiguous portions of `dat`.

    This function implicitly assumes that all points in `dat` fall
    within `lims`.

    """
    jump = np.nonzero(np.abs(np.diff(dat)) > ((lims[1] - lims[0]) * thresh))[0]
    lasti = 0
    for ind in jump:
        yield slice(lasti, ind + 1)
        lasti = ind + 1
    yield slice(lasti, len(dat))

plt.figure(figsize = (4,10))

plt.subplot(7,1,1)
for i in range(3):
    plt.plot(angles_raw[:,i],'.',c=cmpl[i+1],markersize = .7)
plt.subplot(7,1,2)
for i in range(3):
    plt.plot(angles_kalman[:,i],'.',c=cmpl[i+1],markersize = 1.5)
    
# plt.plot(angles_q,'.')
#plt.plot(angles_q_smooth,'.')
for i in range(3):
    plt.subplot(7,1,3+i)
    plt.plot(angles_q[:,i],'.',c=cmpl[i+1])

for i in range(3):
    plt.subplot(7,1,3+i)
    for slc in unlink_wrap(angles_q_smooth[:,i]):
        xx = np.arange(angles_q_smooth.shape[0])
        yy = angles_q_smooth[:,i]
        plt.plot(xx[slc],yy[slc],'-',c='k',markersize=.3)

# plt.xlim(49000,53000)
for i in range(7):
    plt.subplot(7,1,1+i)
    plt.xlim(30000,36000-3600)
    sc = 1.2
    plt.ylim([-np.pi*sc,np.pi*sc])
    ax = plt.gca()
    ax.axis('off')
    
plt.subplots_adjust(hspace=0,wspace=0)
if False:
    plt.savefig('/home/chrelli/git/3d_sandbox/mouseposev0p2/figure_raw_pics'+
            '/supplementary_body_points/smooth_angles.png',transparent=True,
            bbox_inches = 'tight',pad_inches =1)

plt.show()
```

```
/home/chrelli/anaconda2/envs/mousepose/lib/python3.6/site-packages/ipykernel_launcher.py:37: MatplotlibDeprecationWarning: Adding an axes using the same arguments as a previous axes currently reuses the earlier instance.  In a future version, a new instance will always be created and returned.  Meanwhile, this warning can be suppressed, and the future behavior ensured, by passing a unique label to each axes instance.
/home/chrelli/anaconda2/envs/mousepose/lib/python3.6/site-packages/ipykernel_launcher.py:45: MatplotlibDeprecationWarning: Adding an axes using the same arguments as a previous axes currently reuses the earlier instance.  In a future version, a new instance will always be created and returned.  Meanwhile, this warning can be suppressed, and the future behavior ensured, by passing a unique label to each axes instance.
```

In [24]:

```
# Make a figure to show the kalman smoothing!
# plt.close('all')
fsz = (4,10)
lw1 = 1
plt.figure(figsize=fsz)
axs = []
type_list = np.array(['hip','tail','mid','nose','tip','impl'])
for sub_i,i in enumerate([1,0,2,3,5,4]):
    N_plots = 7
    plt.subplot(N_plots,1,1+sub_i)
    ax = plt.gca()
    axs.append(ax)
    for j in range(3):
        plt.plot(body_support_0[i].squeeze().cpu().numpy()[:,j],'.',c=cmpl[1+j],markersize = .7)
        plt.plot(body_support_0_smooth[i][:,j],c='k',lw=lw1)
#         plt.plot(body_support_0_smooth[i][:,j],c=cmpl[-1],lw=lw1)
    plt.ylabel(type_list[i])
    plt.ylim([-.22,.22])

    plt.subplot(N_plots,1,7)
    ax = plt.gca()
    axs.append(ax)
    plt.plot(s_0,c=cmpl[1])
    plt.plot(s_0_smooth,c='k')
    plt.ylabel('s')
    plt.ylim([-1,1])

for ax in axs:
    ax.set_xlim(30000,36000-3600)
    ax.axis('off')
# plt.subplots_adjust(hspace=0,wspace=0)    

if False:
    plt.savefig('/home/chrelli/git/3d_sandbox/mouseposev0p2/figure_raw_pics'+
            '/supplementary_body_points/smooth_xyz0.png',transparent=True,
            bbox_inches = 'tight',pad_inches =1)

plt.show()
```

```
/home/chrelli/anaconda2/envs/mousepose/lib/python3.6/site-packages/ipykernel_launcher.py:20: MatplotlibDeprecationWarning: Adding an axes using the same arguments as a previous axes currently reuses the earlier instance.  In a future version, a new instance will always be created and returned.  Meanwhile, this warning can be suppressed, and the future behavior ensured, by passing a unique label to each axes instance.
```

In [25]:

```
# re-compute the nose-tip and hip-tail end, after correcting the 3D rotations and smooothing s!
#c_hip =  c_mid + R_body @ np.array([-s,0,0])
from utils.analysis_tools import a_hip_0,a_hip_delta,b_hip_0,b_hip_delta,a_nose

c_hip,c_ass,c_mid,c_nose,c_tip,c_impl,R_body,R_head,R_nose = self.body_support_0_smooth
s = self.s_0_smooth

# calculate vectors holding the hip values!
# the values are n_particles long
a_hip = a_hip_0.cpu().numpy() + a_hip_delta.cpu().numpy() * s
b_hip = b_hip_0.cpu().numpy() + b_hip_delta.cpu().numpy() * (1.-s)


c_ass_smooth = np.einsum('aij,aj->ai',R_body,np.hstack([-a_hip,np.zeros_like(s),np.zeros_like(s)]) ) + c_mid

c_ass_smooth = np.einsum('aij,aj->ai',R_body,np.hstack([-a_hip,np.zeros_like(s),np.zeros_like(s)]) ) + c_mid


c_tip_smooth = R_nose@np.array([a_nose.cpu().numpy()[0],0.,0.]) + c_nose

plt.close('all')

axs = []

plt.figure(figsize = (4,10))

plt.subplot(7,1,1)
axs.append(plt.gca())
for i in range(3):
    plt.plot(c_tip[:,i],'.',c = cmpl[1+i])
    plt.plot(c_tip_smooth,'-k')
plt.xlim([30000,32400])

plt.subplot(7,1,2)
axs.append(plt.gca())
for i in range(3):
    plt.plot(c_ass[:,i],'.',c = cmpl[1+i])
plt.plot(c_ass_smooth,'-k')
plt.xlim([30000,32400])

plt.subplot(7,1,7)
axs.append(plt.gca())


for ax in axs:
    ax.set_xlim(30000,36000-3600)
    ax.axis('off')
plt.subplots_adjust(hspace=0,wspace=0)    
if False:
    plt.savefig('/home/chrelli/git/3d_sandbox/mouseposev0p2/figure_raw_pics'+
            '/supplementary_body_points/smooth_tipass.png',transparent=True,
            bbox_inches = 'tight',pad_inches =1)
plt.show()
```

# Let's make a video with an examples of each social behavior¶

In [133]:

```
# Make example plots for smoothing!
# and add the smoothed and raw data to the video maker
from utils.analysis_tools import particles_to_body_supports_cuda,VideoPlotMachine

Plotter = VideoPlotMachine(tracked_behavior,jagged_lines)
self = Plotter

# add the smoothed coordinates as numpy arrays
self.body_support_0_raw = [i.cpu().numpy().squeeze() for i in body_support_0]
self.body_support_0_smooth = body_support_0_smooth
self.s_0_raw = s_0
self.s_0_smooth = s_0_smooth
self.body_support_1_raw = [i.cpu().numpy().squeeze() for i in body_support_1]
self.body_support_1_smooth = body_support_1_smooth
self.s_1_raw = s_1
self.s_1_smooth = s_1_smooth
# also smooth the body ellipsoid rotations
# just by skeleton
self.body_support_0_smooth = smooth_rotation_matrices(body_support_0_smooth)
self.body_support_1_smooth = smooth_rotation_matrices(body_support_1_smooth)
# By skeleton & running quaternion 
# self.body_support_0_smooth = smooth_rotation_matrices_quaternion(body_support_0_smooth)
# self.body_support_1_smooth = smooth_rotation_matrices_quaternion(body_support_1_smooth)
# and re-compute the nose tip and tail
```

In [31]:

```
# Make videos for example social events from the figure! (detected in previous notebook)
event_frames = [18969,36343,17100]
event_name = ['Nose0 <-> Nose1','Nose0 -> Tail1','Nose1 -> Tail0']
```

In [32]:

```
# Plot the examples
plt.close('all')
for i in range(len(event_frames)):    
    Plotter.make_me(event_frames[i],cloud = True,skel='smooth',ellip='smooth',
             trace='smooth',view_override = [55.0,90.+18])
```

In [33]:

```
# now, we make the actual videos. They have three parts:
# - 1) a chunk of time before the social event
pre_time = 8 # frames (half frame rate)
# - 2) a rotation around the social event
# - 3) a chunk of time after the social event
post_time = 8 # frames


video_folder = 'videos'
dpi = 60

for event_number in tqdm(range(len(event_frames))):
    # before
    frame_window = np.hstack([np.arange(-pre_time*10,0)]).astype('int')*3
    frame_list = frame_window + event_frames[event_number]
    savepath = video_folder+'/pre_'+str(event_number) + '.mp4'
    if True:
        Plotter.video_me(frame_list=frame_list,cloud = True,skel='smooth',ellip='smooth',
             trace='smooth',savepath = savepath,view_override = [20,50],dpi=dpi,time_offset = -frame_window[0]/60)
    
    # after
    frame_window = np.hstack([np.arange(0,post_time*10)]).astype('int')*3
    frame_list = frame_window + event_frames[event_number]
    savepath = video_folder+'/post_'+str(event_number) + '.mp4'
    if True:
        Plotter.video_me(frame_list=frame_list,cloud = True,skel='smooth',ellip='smooth',
             trace='smooth',savepath = savepath,view_override = [20,50],dpi=dpi)
    
    # rotation
    savepath = video_folder+'/rotation_'+str(event_number) + '.mp4'
    if True:
        Plotter.rotation(frame_list=frame_list,cloud = True,skel='smooth',ellip='smooth',
             trace='smooth',savepath=savepath,view_override = [20,50],dpi=dpi)
    plt.close('all')
```

```
100%|██████████| 3/3 [01:29<00:00, 29.91s/it]
```

# Now, stack all the vidoes together, using cv2¶

In [35]:

```
# merge the videos using opencv, from here: https://gist.github.com/nkint/8576156
import cv2
import os

n_videos = 3
video_folder = 'videos'
video_files = [video_folder+'/pre_{:01d}.mp4'.format(i) for i in range(n_videos)]
caps = [cv2.VideoCapture(video_files[i]) for i in range(n_videos)]

width  = caps[0].get(cv2.CAP_PROP_FRAME_WIDTH)   # float
height = caps[0].get(cv2.CAP_PROP_FRAME_HEIGHT)  # float

b_cut = 12
s_cut = 40

fourcc = cv2.VideoWriter_fourcc(*'MP4V')
out_fps = 20
out = cv2.VideoWriter('videos/supplementary_video_social.mp4', fourcc, out_fps, (int(n_videos*width-6*s_cut), int(height-b_cut)))

# create a splash screen
# create blank image
img = np.zeros((int(height-b_cut),int(n_videos*width-6*s_cut), 3), np.uint8)
font = cv2.FONT_HERSHEY_SIMPLEX
font_color = (255, 255, 255)
h = height-.1*height
w = (n_videos*width-6*s_cut)/2
font_scale = 1.4
thickness = 2
text = 'Ebbesen & Froemke, 2020'
def put_centered_text(img,text,w,h,font, font_scale, font_color, thickness):
    # get boundary of this text
    textsize = cv2.getTextSize(text, font, font_scale, thickness)[0]
    cv2.putText(img, text, (int(w - textsize[0]/2),int(h) ), font, font_scale, font_color, thickness, cv2.LINE_AA)

put_centered_text(img,text,w,h,font, font_scale, font_color, thickness)
put_centered_text(img,'Supplementary video 4: Social events',w,.2*height,font, font_scale, font_color, thickness)

for _ in range(30):
    cv2.imshow('frame',img)
    cv2.waitKey(10)
    out.write(img)
    

        
# Add annotation!
# centering is not easy, in cv2: https://gist.github.com/xcsrz/8938a5d4a47976c745407fe2788c813a
font = cv2.FONT_HERSHEY_SIMPLEX
font_color = (0,0,0)
h = .07*height
w = width/2
font_scale = 1
thickness = 2


def add_files(video_files,c=(0,0,0)):
    caps = [cv2.VideoCapture(video_files[i]) for i in range(n_videos)]

    while(caps[0].isOpened()):
        ret, frame_0 = caps[0].read()
        ret, frame_1 = caps[1].read()
        ret, frame_2 = caps[2].read()
        if frame_1 is not None:
            stacked_frame = cv2.hconcat([frame_0[:-b_cut,s_cut:-s_cut,:],frame_1[:-b_cut,s_cut:-s_cut,:],frame_2[:-b_cut,s_cut:-s_cut,:]])
            
            
            put_centered_text(stacked_frame,'Nose_0 <-> Nose_1',w - s_cut,h,font, font_scale, font_color, thickness)
            put_centered_text(stacked_frame,'Nose_0 -> Tail_1',w+width - 3* s_cut,h,font, font_scale, font_color, thickness)
            put_centered_text(stacked_frame,'Nose_1 -> Tail_0',w+2*width- 5 * s_cut,h,font, font_scale, font_color, thickness)

            cv2.imshow('frame',stacked_frame)
            cv2.waitKey(10)
            out.write(stacked_frame)
        else:
            break

cc = (10,10,10)            
video_files = [video_folder+'/pre_{:01d}.mp4'.format(i) for i in range(n_videos)]
add_files(video_files, c = cc)     
video_files = [video_folder+'/rotation_{:01d}.mp4'.format(i) for i in range(n_videos)]
add_files(video_files, c= cc)     
video_files = [video_folder+'/post_{:01d}.mp4'.format(i) for i in range(n_videos)]
add_files(video_files,c = cc)             
        
caps[0].release()
caps[1].release()
caps[2].release()
out.release()
cv2.destroyAllWindows()

print ("end.")
```

```
end.
```
