# Supplementary material for: Automatic mapping of multiplexed social receptive fields by deep learning and GPU-accelerated 3D videography
Source: Nat Commun. 2022 Feb 1;13:593. doi: 10.1038/s41467-022-28153-7 (PMC8807631; doi:10.1038/s41467-022-28153-7)
Supplement: Supplementary file 9 — Supplementary Software [file 41467_2022_28153_MOESM9_ESM.zip › ebbesen_froemke_2021_code/analysis/011_Align_tracking_data_to_electrophysiology.html]

011\_Align\_tracking\_data\_to\_electrophysiology


In [1]:

```
# IDEA: Add neck to the posture map?
from IPython import get_ipython

# %matplotlib qt
%load_ext autoreload
%autoreload 2

import time, os, sys, shutil
from utils.fitting_utils import *

# for math and plotting
import pandas as pd
import numpy as np
import scipy as sp
import matplotlib.pyplot as plt
#%matplotlib notebook
#%matplotlib widget
import scipy.stats as stats

from itertools import compress # for list selection with logical
from tqdm import tqdm

from multiprocessing import Process

# and pytorch
import torch

import sys, os, pickle
# import cv2
from colour import Color
import h5py
from tqdm import tqdm, tqdm_notebook
import glob
import itertools
```

# Load timing data from tracking and ephys¶

In [2]:

```
# point to the experiment folder
tracking_folder = 'example_data/tracking'
TTL_folder = 'example_data/experiment1/recording1/events/Rhythm_FPGA-100.0/TTL_1'
```

In [3]:

```
# load the geometry
import pickle
geometry = pickle.load( open( tracking_folder+'/geometry.pkl', "rb" ) ) 
timing = pickle.load( open( tracking_folder+'/timing.pkl', "rb" ) )
behavior = pickle.load( open( tracking_folder+'/tracked_behavior.pkl', "rb" ) )

print(geometry.keys())
print(timing.keys())
plt.close('all')
```

```
dict_keys(['start_frame', 'end_frame', 'd_cam_params', 'c_cam_params', 'R_extrinsics', 't_extrinsics', 'R_world', 't_world', 'M0', 'floor_point', 'floor_normal', 'c_cylinder', 'r_cylinder'])
dict_keys(['master_frame_table', 'reference_time_cam', 'reference_stamps', 'time_stamps', 'shifted_stamps', 'aligned_to_ephys'])
```

In [4]:

```
# load the behavior LED and time stamps, IN SECONDS
raw_shifted = timing['time_stamps'][timing['reference_time_cam']]
LED_behavior_raw = raw_shifted[:,-1]
raw_shifted = timing['shifted_stamps'][timing['reference_time_cam']]
LED_behavior = raw_shifted[:,-1]
ts_behavior = timing['reference_stamps'] / 1e3

plt.figure()
plt.plot(ts_behavior,LED_behavior)
plt.xlabel('Time [s]')
plt.ylabel('LED lum')

plt.title('Camera LED trace')
plt.xlim([0,20])
plt.show()
```

# Rough aligment by simple shift¶

In [5]:

```
# ALSO load the ephys LED and time stamps, IN SECONDS
TTL_stamps = np.load(TTL_folder+'/timestamps.npy')
TTL_words = np.load(TTL_folder+'/full_words.npy')
TTL_states = np.load(TTL_folder+'/channel_states.npy')
TTL_channels = np.load(TTL_folder+'/channels.npy')

TTL_sample_rate = 30e3

LED_channel = 7

LED_oeph = TTL_states[TTL_channels == LED_channel]
# open ephys sits high with no LED light, so turn around
LED_oeph = (-LED_oeph /LED_channel + 1.)/2

ts_oeph = TTL_stamps[TTL_channels == LED_channel] / TTL_sample_rate 

plt.figure()
plt.step(ts_oeph,LED_oeph)
plt.plot(ts_oeph,LED_oeph,':or')
plt.xlabel('Time [ms]')
plt.ylabel('TTL state')
plt.title('oeph LED trace')
plt.xlim(ts_oeph[0]+np.array([-5,10]))
plt.show()
```

In [6]:

```
# make a function to update the timing with an aligned time!
# LED_oeph,ts_oeph,LED_behavior,ts_behavior, show_plots = True
# align_to_refernces
LED_ref,ts_ref,LED_video,ts_video = LED_oeph,ts_oeph,LED_behavior,ts_behavior
show_plots = True
plt.close('all')

# zero to the first onset (roughly)
# now we find 'big steps' which are big increases!
led_diff = np.diff(LED_video)
big_steps = np.hstack([False, led_diff > .5 ])
# set the first few to false to remove possible noise
big_steps[0:30] = False

bonsai_led_on = ts_video[big_steps]
bonsai_internal_offset = bonsai_led_on[0]
bonsai_led_zeroed = bonsai_led_on-bonsai_internal_offset;

# also find the onset times in the open ephys!
led_diff = np.diff(LED_ref)
# big_steps = np.hstack([False, led_diff > .5 ])
# ref_led_on = ts_ref[big_steps]

# BY DESIGN the TTL flips are 0 when the LED is on
ref_led_on = ts_ref[LED_ref == 0.]

# ~~~~~~~~~~~~
# Process (zero) the open ephys to find first blink of a recording blink (on) times!
# ~~~~~~~~~~~~
# find the LED start of the recordings
# these have extremely long time differences, like above 20x the median for example
big_skips = ref_led_on[ np.hstack( ( False , np.diff(ref_led_on) > 20*np.median(np.diff(ref_led_on)) ) ) ]
first_leds = np.hstack( (ref_led_on[0] , big_skips ) )

# NOW here we zero the open ephys time to the first blink in that recording!
# we zero the open ephys to that first led!
rec_no = 0 # allow fo change the recording to align to
internal_open_offset = first_leds[rec_no];
open_led_zeroed = ref_led_on[ref_led_on >= internal_open_offset] - internal_open_offset;
```

In [7]:

```
# from : http://jonathansoma.com/lede/data-studio/matplotlib/changing-fonts-in-matplotlib/
# Along with matplotlib.pyplot, for this one
# you also need to import matplotlib itself
import matplotlib

# Say, "the default sans-serif font is COMIC SANS"
matplotlib.rcParams['font.sans-serif'] = "Liberation Sans"
# Then, "ALWAYS use sans-serif fonts"
matplotlib.rcParams['font.family'] = "sans-serif"

matplotlib.rc('font', family='sans-serif') 
matplotlib.rc('text', usetex='false') 
matplotlib.rcParams.update({'font.size': 15})

import matplotlib.font_manager
flist = matplotlib.font_manager.get_fontconfig_fonts()
names = [matplotlib.font_manager.FontProperties(fname=fname).get_name() for fname in flist]
```

In [8]:

```
from palettable.cmocean.sequential import Algae_6


if show_plots and False:
#     plt.close('all')
    plt.figure()
    plt.plot(ts_video,LED_video)
    for i in range(100):
        plt.axvline(bonsai_led_on[i],c='r')
    plt.xlim([0,20])
    plt.title('video LED not aligned')
    plt.xlabel('Time [s]')
    plt.show()
    
if show_plots and False:
#     plt.close('all')
    plt.figure()
    plt.plot(ts_ref,LED_ref)
    for i in range(100):
        plt.axvline(ref_led_on[i],c=Algae_6.mpl_colors[1])
    plt.xlim( np.array([0,20]) + ts_ref[0] )
    plt.title('open epehys LED not aligned')
    plt.xlabel('Time [s]')
    plt.show()
    
if show_plots:
#     plt.close('all')
    plt.figure(figsize=(6,3))
    plt.subplot(2,1,1)
#     plt.plot(ts_video-bonsai_internal_offset,LED_video*255)
    
    plt.plot(ts_video-bonsai_internal_offset,LED_behavior_raw,'k')
    
    for i in range(np.min([100,len(bonsai_led_on)])):
        plt.axvline(bonsai_led_on[i]-bonsai_internal_offset,c=Algae_6.mpl_colors[2])
    plt.xlim( np.array([-5,15]) )
#     plt.title('Onset times for alignment')
    plt.ylabel('8-bit im')
#     plt.legend(["raw trace","onset times"])
    ax = plt.gca()
    ax.spines['left'].set_bounds(100,255)

    plt.plot(ts_video-bonsai_internal_offset,LED_behavior_raw,'k')
    plt.xticks([])

    ax = plt.gca()
    ax.spines['right'].set_visible(False)
    ax.spines['top'].set_visible(False)
    ax.spines['bottom'].set_visible(False)
    plt.yticks([100,255])
    ax.spines['left'].set_bounds(100,255)
    
    plt.subplot(2,1,2)
#     plt.plot(ts_ref-internal_open_offset,LED_ref,':o')
    for i in range(100):
        plt.axvline(ref_led_on[i]-internal_open_offset,c=Algae_6.mpl_colors[2],alpha=1.)
    plt.xlim( np.array([-5,15]) )
#     plt.title('open epehys LED zeroed')

    xxx =ts_ref-internal_open_offset
    yyy = LED_ref
    xxx = np.hstack([-10,xxx])
    yyy = np.hstack([.0,yyy])
    plt.step(xxx,yyy,':',color = 'k')
    plt.plot(xxx,1-yyy,'o',color = 'k')

    plt.xlabel('Time [s]')
#     plt.legend(["TTL flips","onset times"])
    plt.ylabel('TTL logic')
    ax = plt.gca()
    ax.set_yticks([0,1])
    plt.ylim([-.1,1.1])
    
    ax.spines['right'].set_visible(False)
    ax.spines['top'].set_visible(False)
    ax.spines['left'].set_bounds(0,1)
    
    
    for ii in range(2):
        plt.subplot(2,1,1+ii)
        plt.xlim( np.array([-1,6]) )
        sst = 1200.
#         plt.xlim( np.array([-1+sst,6+sst]) )


#     for ii in range(2):
#         plt.subplot(2,1,1+ii)
#         plt.xlim( np.array([-2+1000,8.5+1000]) ) 

#     plt.savefig('figure_raw_pics/figure_1/LED_trace.pdf',bbox_inches = 'tight', pad_inches = 0)
    plt.show()
```

```
/home/chrelli/anaconda3/envs/myp/lib/python3.6/site-packages/ipykernel_launcher.py:80: MatplotlibDeprecationWarning: Adding an axes using the same arguments as a previous axes currently reuses the earlier instance.  In a future version, a new instance will always be created and returned.  Meanwhile, this warning can be suppressed, and the future behavior ensured, by passing a unique label to each axes instance.
```

# Refine alignment¶

In [9]:

```
# ~~~~~~~~~~~~
# Use cross correlation to do a finer alignment, we go for 40 s +/- around the first seen blink
# (this is if someone forgets which recording to start first)
# ~~~~~~~~~~~~
# bin the big steps at 10 ms resolution!
test_a = open_led_zeroed
test_b = bonsai_led_zeroed

if show_plots and False:
    plt.figure()
    plt.plot(test_a)
    plt.plot(test_b)
    plt.show()
    
bins = np.arange(0,40,.01) # do 20 s!
# bin the big steps at 20 ms resultions!
count_a, bins_a = np.histogram(test_a,bins=bins)
count_b, bins_b = np.histogram(test_b,bins=bins)

count_a = np.convolve(count_a,np.ones(5),mode='same')
count_b = np.convolve(count_b,np.ones(5),mode='same')

if show_plots and False:
    # show the binned traces
    plt.figure(figsize=(15,5))
    plt.step(bins_a[:-1],count_a)
    plt.step(bins_b[:-1],count_b)
    plt.plot()

from scipy import signal
crosscorr = signal.correlate(count_a, count_b, mode='full',method ='direct')
lags = np.hstack((np.flip(-bins[1:-1]),bins[:-1]))

lag_winner = lags[np.argmax(crosscorr)]
if show_plots:
    # show cross corelellogram
    plt.figure(figsize=(16,4))
    plt.plot(lags,crosscorr)
    plt.axvline(lag_winner,c='r')
    plt.plot(lags,crosscorr,c='k')
    plt.title(lag_winner)
    plt.xlabel('shift [s]')
    plt.ylabel('xcorr')

    plt.show()
```

# Correct for drift¶

In [10]:

```
# ~~~~~~~~~~~~
# Find the alignement by robust regerssion
# ~~~~~~~~~~~~
# to find the real alignment and 
# Calculate all differences as a big matrix!, only use differences smaller than one second!
# use broadcasting to calculate the big matrix! It will be open_ephys X bonsai
# we align by the lag as well here!
all_differences = open_led_zeroed[:,np.newaxis] - bonsai_led_zeroed - lag_winner;

# find min pr column so min pr bonsai blink
min_location = np.argmin(np.abs(all_differences),0)
# get a list of the smallest distance of the bonsai blinks to an open ephys bit flip
min_diff = all_differences[min_location,np.arange(len(min_location))] 
# only use residuals which are kind of reasonable, like abs residual z<2
abs_diff_small = np.abs(stats.zscore(min_diff)) < 2.;

# fit a robust regression
res = stats.theilslopes(min_diff[abs_diff_small], bonsai_led_on[abs_diff_small], 0.70)
bonsai_offset = res[1];
bonsai_slope = res[0];

# and do the alignment! # remember to add the lag_winner as well!
bonsai_time_aligned = (1+bonsai_slope) * (ts_video - bonsai_internal_offset + lag_winner) + internal_open_offset + bonsai_offset;
# %% For comparison
bonsai_time_rough = (ts_video - bonsai_internal_offset) + internal_open_offset
```

In [11]:

```
# What's thr drift?
print("Offset is {} and slope is {}".format(bonsai_offset,bonsai_slope))
print("Offset is {} and slope is {} ms/min".format(bonsai_offset,bonsai_slope*1e3*60))
```

```
Offset is 0.0043639021557598 and slope is -8.118949221169916e-07
Offset is 0.0043639021557598 and slope is -0.048713695327019496 ms/min
```

In [12]:

```
from palettable.cmocean.sequential import Algae_6


if show_plots:
    # do the diagnosis plot of the regression!
    fig = plt.figure(figsize=(5,3))
    plt.subplot(1,2,1)
    plt.plot(bonsai_led_on/60,(min_diff - np.mean(min_diff) )*1e3,'.',c=Algae_6.mpl_colors[1])
#     plt.xlabel('recording time [min]')
    plt.ylabel('Residual [ms]')

    # hold on
    ax = plt.gca()
    xx = np.array( ax.get_xlim() )
    yy = bonsai_offset + bonsai_slope * xx*60 - np.mean(min_diff)

#     plt.plot(xx,yy*0e3,'-k')
    plt.plot(xx,yy*1e3,'--k')
#     plt.ylim([-.015,.015])

    plt.subplot(1,2,2)
    # % calculate the absolute residuals
    residuals = min_diff - (bonsai_offset + bonsai_slope  * bonsai_led_on);
    plt.plot(bonsai_led_on/60,residuals*1e3,'.',c=Algae_6.mpl_colors[1])
#     plt.ylabel('residual [ms]')
#     plt.xlabel('recording time [min]')
    plt.yticks([])
#     plt.plot(xx,yy*0,'-k')

    plt.plot(xx,yy*0+np.median(residuals),'--k')
#     plt.ylim([-.01,.01])

    for ii in [1,2]:
        plt.subplot(1,2,ii)
        plt.xlim([-1,22])
        plt.ylim([-9,9])
        plt.ylim([-12,12])
        ax = plt.gca()
        ax.spines['right'].set_visible(False)
        ax.spines['top'].set_visible(False)
        ax.spines['left'].set_bounds(-10,10)
        ax.spines['bottom'].set_bounds(0,20)

    ax.spines['left'].set_visible(False)

    
    fig.add_subplot(111, frameon=False)
    # hide tick and tick label of the big axis
    plt.tick_params(labelcolor='none', top=False, bottom=False, left=False, right=False)
    plt.xlabel("Recording time [min]")
    # plt.ylabel("common Y")       
    
    
    ax.spines['left'].set_bounds(-10,10)
    
#     plt.savefig('figure_raw_pics/figure_1/slopes.pdf',bbox_inches = 'tight', pad_inches = 0)
    plt.show()
```

```
/home/chrelli/anaconda3/envs/myp/lib/python3.6/site-packages/ipykernel_launcher.py:34: MatplotlibDeprecationWarning: Adding an axes using the same arguments as a previous axes currently reuses the earlier instance.  In a future version, a new instance will always be created and returned.  Meanwhile, this warning can be suppressed, and the future behavior ensured, by passing a unique label to each axes instance.
```

In [13]:

```
# add the aligned time to the behavior timing!
timing['aligned_to_ephys'] = bonsai_time_aligned
```

In [14]:

```
# add the aligned time to the behavior timing and re-save!
with open(tracking_folder+'/timing.pkl', 'wb+') as f:
    pickle.dump(timing,f)
```

# Plot some figures for the ms¶

In [15]:

```
from palettable.colorbrewer.sequential import Blues_8,Greens_5
from palettable.cmocean.sequential import Algae_6

plt.figure(figsize=(8,2))

ax = plt.gca()
# ax.set_prop_cycle('color', Greens_5.mpl_colors[1:])
ax.set_prop_cycle('color', Algae_6.mpl_colors[1:])

cam = 2
for cam in range(4):
    stamps=timing['time_stamps'][cam]

    f_idx = stamps[:,0]
    f_n = stamps[:,1]

#     plt.subplot(4,1,1+cam)
#     plt.plot(f_idx[:-1],np.diff(stamps[:,3])*1e3+15*cam,label='unix',c = 'gray')
    plt.plot(f_n[:-1],np.diff(stamps[:,2])+1*cam,label='hw')

    plt.plot([-2000,-1000],np.array([1000/60.,1000/60.])+1*cam,c='k')


    

    
plt.plot(np.array([0.,60*60*2.])+70000,np.array([0,0])+16,c='k')
plt.plot(np.array([60*60*2.,60*60*2.])+70000,np.array([16,17]),c='k')
    
    
#     plt.legend()
#     plt.xlim([2000,200000])
#     plt.xlim([2300,2400])

plt.ylim(15,20)
ax.spines['right'].set_visible(False)
ax.spines['left'].set_visible(False)

ax.spines['top'].set_visible(False)
ax.spines['bottom'].set_bounds(0,80000)

# plt.axis('tight')
plt.yticks([])

plt.xlabel('Frame #')
plt.ylabel('$\Delta$timestamp')


# plt.axis('tight')
# plt.savefig('figure_raw_pics/figure_1/frames.pdf', pad_inches = 0.3)
#plt.savefig('figure_raw_pics/figure_1/frames.pdf',bbox_inches = 'tight', pad_inches = 0)

plt.show()
```

In [52]:

```
# calc the median jitter 
dts = []
for cam in range(4):
    stamps=timing['time_stamps'][cam][:,2]
    dts.append(stamps)
    
dts = np.hstack(dts)
print(dts.shape)
diffs = np.diff(dts)
diffs = diffs[diffs>0]
print(diffs[0])
print("jitter: {} ms".format(np.std(diffs)))
```

```
(299339,)
16.73388671875
jitter: 0.02578912656231456 ms
```

In [20]:

```
plt.figure(figsize=(1.5,2))
ax = plt.gca()
ax.set_prop_cycle('color', Algae_6.mpl_colors[1:])

cam = 2
for cam in range(4):
    stamps=timing['time_stamps'][cam]

    f_idx = stamps[:,0]
    f_n = stamps[:,1]

#     plt.subplot(4,1,1+cam)
    plt.plot(f_idx[:-1],np.diff(stamps[:,3])*1e3+1*cam,label='unix',c = 'gray')
    plt.plot(f_n[:-1],np.diff(stamps[:,2])+1*cam,label='hw')
#     plt.legend()
#     plt.xlim([2000,200000])
#     plt.xlim([2440,2470])
    plt.xlim([58650,58680])

    
plt.ylim(15,20)
plt.yticks([])

# plt.axis('tight')
ax.spines['right'].set_visible(False)
ax.spines['top'].set_visible(False)
ax.spines['left'].set_visible(False)
ax.spines['top'].set_visible(False)

plt.xlabel('Frame #')
# plt.axis('tight')
# plt.savefig('figure_raw_pics/figure_1/frames_zoom.pdf', pad_inches = 10)

#plt.savefig('figure_raw_pics/figure_1/frames_zoom.pdf',bbox_inches = 'tight', pad_inches = 0)
plt.show()
```

In [21]:

```
plt.figure(figsize=(2,2))
ax = plt.gca()
ax.set_prop_cycle('color', Algae_6.mpl_colors[1:])

cam = 2

nd = []
pctd = []
for cam in range(4):
    stamps=timing['time_stamps'][cam]

    f_idx = stamps[:,0]
    f_n = stamps[:,1]
    n_unique = np.unique(f_n).shape[0]
    n_all = np.unique(f_n).shape[0]
    n_dropped = n_unique-n_all
    
    all_diffs = np.diff(stamps[:,2])
    n_dropped = np.sum(all_diffs < 10)
    pct_dropped = n_dropped/np.shape(all_diffs)[0]
    nd.append(n_dropped)
    pctd.append(pct_dropped)

rects1 = plt.bar(np.arange(4),nd)

# for iii in range(4):    
#     plt.bar(iii,nd[iii],color = Algae_6.mpl_colors[iii-4])

    
    
ax = plt.gca()
ax.set_xticks(np.arange(4))
ax.set_xticklabels(['cam 0','cam 1','cam 2','cam 3'],rotation=90.)
ax.set_yticks(np.arange(0,11,2))

# for iii in range(4):    
#     plt.bar(ii,nd[ii])

def autolabel(rects):
    """Attach a text label above each bar in *rects*, displaying its height."""
    for rect in rects:
        height = rect.get_height()
        ax.annotate('{}'.format(height),
                    xy=(rect.get_x() + rect.get_width() / 2, height),
                    xytext=(0, 3),  # 3 points vertical offset
                    textcoords="offset points",
                    ha='center', va='bottom')


autolabel(rects1)

plt.ylabel("Dropped frames")
plt.yticks([0,10])
ax.spines['right'].set_visible(False)
ax.spines['top'].set_visible(False)
# ax.spines['bottom'].set_bounds(-.3,3.3)

#plt.savefig('figure_raw_pics/figure_1/dropped_count.pdf',bbox_inches = 'tight', pad_inches = 0)


plt.show()
```
