# Supplementary material for: Automatic mapping of multiplexed social receptive fields by deep learning and GPU-accelerated 3D videography
Source: Nat Commun. 2022 Feb 1;13:593. doi: 10.1038/s41467-022-28153-7 (PMC8807631; doi:10.1038/s41467-022-28153-7)
Supplement: Supplementary file 9 — Supplementary Software [file 41467_2022_28153_MOESM9_ESM.zip › ebbesen_froemke_2021_code/analysis/003_Train_hourglass_network.html]

003\_Train\_hourglass\_network


In [1]:

```
# FROM TIRAMISU
# IDEA: Add neck to the posture map?
%matplotlib inline
%load_ext autoreload
%autoreload 2

import time
from pathlib import Path
import numpy as np
import matplotlib.pyplot as plt

import torch
import torch.nn as nn
import torch.optim as optim
import torchvision
import torchvision.transforms as transforms

# import the tiramisu models
#from pytorch_tiramisu.models import tiramisu

# from pose.models import hourglass
#import pose2d.models as hourglass_models

# from datasets import camvid
# from pytorch_tiramisu.datasets import joint_transforms
# import pytorch_tiramisu.utils.imgs
# import pytorch_tiramisu.utils.training as train_utils

import sys, os, pickle
import cv2
from colour import Color
import h5py
```

In [2]:

```
# Check CUDA
print('Cuda:')
print(torch.cuda.is_available())
print(torch.cuda.device_count())
print(torch.cuda.get_device_name(0))
```

```
Cuda:
True
1
GeForce RTX 2080 Ti
```

# Check the training data is there and declare dataset and loaders¶

In [3]:

```
# Make sure the images are in the file
h5_path = 'training_sets/mouse_training_set_labeled.h5'
h5_file = h5py.File(h5_path, 'r')

from c_utils.utils_hour import check_h5
check_h5(h5_path)
```

```
<KeysViewHDF5 ['annotated', 'annotations', 'c_images', 'reference_index', 'skeleton']>
```

In [4]:

```
# the images have to be repeatedly divisible with two, so these are the options
exp = np.arange(1,12)
exp = 2**exp
print(1*exp);print(3*exp);print(5*exp);print(7*exp);print(11*exp)
```

```
[   2    4    8   16   32   64  128  256  512 1024 2048]
[   6   12   24   48   96  192  384  768 1536 3072 6144]
[   10    20    40    80   160   320   640  1280  2560  5120 10240]
[   14    28    56   112   224   448   896  1792  3584  7168 14336]
[   22    44    88   176   352   704  1408  2816  5632 11264 22528]
```

In [5]:

```
# make a minimal dataset to loop over the frames!!
# https://stanford.edu/~shervine/blog/pytorch-how-to-generate-data-parallel
import torch
import torch.utils.data as data
import imgaug.augmenters as iaa

from c_utils.utils_hour import gaussian

# HACK for now, numpy had a code change in 1.8
import numpy
numpy.random.bit_generator = numpy.random._bit_generator


selfseq = iaa.Sequential([
        #     iaa.Crop(px=(0, 100)), # crop images from each side by 0 to 16px (randomly chosen)
            iaa.CropAndPad(percent=(-0.10, 0.15), sample_independently=False),
            iaa.Fliplr(0.5), # horizontally flip 50% of the images
            iaa.Sometimes(.3, iaa.GaussianBlur(sigma=(0, 1.5)) ), # blur images with a sigma of 0 to 3.0
            iaa.Sometimes( 1, iaa.Dropout(p = (0,0.2)) ),
            iaa.Affine(rotate=(-30, 30),
                       translate_percent={"x": (-0.2, 0.2), "y": (-0.2, 0.2)})
        ],random_order=True)
   


class MouseDataset(data.Dataset):
    # todo add augmentation here, clean up and make faster
    # todo remove stupid side effects etc
    def __init__(self, h5_data,which_indices,augmentation=False):
        '''Initialization'''
        self.label_names = ['impl','ear','ear','nose','tail','ear','ear','nose','tail']
        self.label_index = [0,1,1,1,1,2,2,3,3]
        
        # index for loading subsets from the h5 file
        self.which_indices = which_indices       
        self.n_images = len(which_indices)
    

        # FOR THE AUGMENTATION PIPELINE

        self.seq = iaa.Sequential([
        #     iaa.Crop(px=(0, 100)), # crop images from each side by 0 to 16px (randomly chosen)
            iaa.CropAndPad(percent=(-0.10, 0.15), sample_independently=False),
            iaa.Fliplr(0.5), # horizontally flip 50% of the images
            iaa.Sometimes(.3, iaa.GaussianBlur(sigma=(0, 1.5)) ), # blur images with a sigma of 0 to 3.0
            iaa.Sometimes( 1, iaa.Dropout(p = (0,0.2)) ),
            iaa.Affine(rotate=(-30, 30),
                       translate_percent={"x": (-0.2, 0.2), "y": (-0.2, 0.2)})
        ],random_order=False)
        
        self.augmentataion = augmentation
       
    def __len__(self):
        'Denotes the total number of samples'
        return self.n_images    
        
    def __getitem__(self, index):
        # will be i x j x 1 - is that ok?
        # todo check if this moveaxis is good?
        # im = np.moveaxis(h5_data['c_images'][self.which_indices[index]],[0,1],[1,2])
#         tracking_folder = '/home/chrelli/Documents/Example3D_compressed'
#         h5_path = tracking_folder + '/mouse_rgbd_annotation_set.h5'
        h5_path = 'training_sets/mouse_training_set_labeled.h5'
        with h5py.File(h5_path, 'r') as h5_file:
            xy = h5_file['annotations'][self.which_indices[index]]
            c_image = h5_file['c_images'][self.which_indices[index]]
            # dac_image = h5_file['dac_images'][self.which_indices[index]]
        
       
        # check if the points are good before augmentation
        # upper left corner is trash
        point_good = (xy[:,0] > 30)*( xy[:,1] > 30)
        
        # NOW, AUGMENT!
        # TODO AUGMENT DEPTH AS WELL?
        # AUGMENT DEPTH BY DEAD BLOCKS TO SIMULATE REAL DATA?
        

        if self.augmentataion:
            #HACK: selfseq instead of self.seq
            images_aug, xy_aug_list = self.seq(images = c_image[np.newaxis,:,:,:], keypoints=[xy])
            xy = xy_aug_list[0]
            c_image = images_aug[0,:,:,:]
        
        # pack depth and pixels to target - OR NOT??
        im = c_image
        frame_height = im.shape[0]
        frame_width = im.shape[1]
        
        # make the resolution correct, i.e. set the height to 192
        pad_top = 8
        pad_bottom = 10
        im = im[pad_top:-pad_bottom,:,:]
        
        # and make the image Chan X heigh X width
        im = np.moveaxis(im,2,0)
        
        # recale the keypoints
        xy[:,1] -= pad_top
            
        rescale = True
        h_out,w_out = im.shape[1],im.shape[2]
        label_sigma = np.array([2,1,1,1,1,1,1,1,1]) *1.5 *4
        gaussian_sigma = (15,15)
        line_thickness = 5
        
        if rescale:
            # halved
            h_out,w_out = int(h_out/4),int(w_out/4)
            xy = np.round(xy/4).astype('int')
            label_sigma = np.array([3,1,1,1,1,1,1,1,1]) 
            gaussian_sigma = (3,3)
            line_thickness = 1

        label_names = ['impl','ear','ear','nose','tail','ear','ear','nose','tail']
        body_names = ['mouse0','mouse0','mouse0','mouse0','mouse0','mouse1','mouse1','mouse1','mouse1']
        label_index = [0,1,1,2,3,1,1,2,3]
        body_index = [0,0,0,0,0,1,1,1,1]

        # target has to be batch x n_features x pic_i x pic_j
        target_points = np.zeros((4,h_out,w_out))
        img = target_points[0,:,:].copy()

        for i in range(9):
            if point_good[i]:
                target_points[label_index[i],:,:] += ( gaussian(img.copy(), xy[i,:], label_sigma[i]) )        

        # draw the lines within the body!
        xy_good = xy[point_good]
        label_good = xy[point_good]
        body_good = xy[point_good]


        target_lines = []

        def draw_lines(p1,p2):
            img_blank = np.zeros((h_out,w_out)).astype('uint8')
            for i1,i2 in zip(p1,p2):
                if point_good[i1]*point_good[i2]:
                    start = tuple(np.round(xy[i1,:]).astype('int') )
                    end = tuple(np.round(xy[i2,:]).astype('int') )
                    cv2.line(img_blank,start,end,[255,255,255],thickness = line_thickness)
                img_blank = np.clip(img_blank,0,255)
                img_blank = cv2.GaussianBlur(img_blank,gaussian_sigma,0)
            img_blank = img_blank/255
            target_lines.append(img_blank.copy())            
            
        # I to ears
        p1,p2 = [0,0],[1,2]
        draw_lines(p1,p2)

        # I to nose
        p1,p2 = [0],[3]
        draw_lines(p1,p2)

        # I to tail
        p1,p2 = [0],[4]
        draw_lines(p1,p2)

        # Ear to Ear
        p1,p2 = [1,5],[2,6]
        draw_lines(p1,p2)

        # Ear to Tail
        p1,p2 = [1,2,5,6],[4,4,8,8]
        draw_lines(p1,p2)

        # Ear to Nose
        p1,p2 = [1,2,5,6],[3,3,7,7]
        draw_lines(p1,p2)

        # Nose to Tail
        p1,p2 = [3,7],[4,8]
        draw_lines(p1,p2)

        # stack all the targets
        target_lines = np.concatenate([t[np.newaxis,:,:] for t in target_lines],axis=0)
        target = np.concatenate((target_points,target_lines),axis = 0)
        
        # convert to sensible ranges
        im = im / 255.
        
        im = np.clip(im,0,1)
        
        # from documentation    
        # For a conv2D, input should be in (N, C, H, W) format. N is the number of samples/batch_size. C is the channels. H and W are height and width resp.
        # See shape documentation at https://pytorch.org/docs/stable/nn.html#torch.nn.Conv2d    
        # so im is (batch, channels, H, W) 
        # target is (n_trained_features, H, W) 
        
        return im.astype('float32'),target.astype('float32')

# # separate by pseudo-random indices
np.random.seed(0)
n_frames = 576
random_indices = np.random.permutation(n_frames)

MouseTrain = MouseDataset(h5_file,random_indices[:],augmentation=True)
MouseValid = MouseDataset(h5_file,random_indices[:50])

# we shuffle, so that we always see different dumps
# MouseTrainLoader = data.DataLoader(MouseTrain, batch_size=1, shuffle=True, num_workers = 1)
MouseTrainLoader = data.DataLoader(MouseTrain, batch_size=1, shuffle=True)
MouseValidLoader = data.DataLoader(MouseValid, batch_size=1, shuffle=True, num_workers = 1)

print("training augment = {}".format(MouseTrain.augmentataion) )
print("validation augment = {}".format(MouseValid.augmentataion) )
```

```
training augment = True
validation augment = False
```

In [6]:

```
self = MouseTrain
index = 10
h5_path = 'training_sets/mouse_training_set_labeled.h5'
with h5py.File(h5_path, 'r') as h5_file:
    xy = h5_file['annotations'][self.which_indices[index]]
    c_image = h5_file['c_images'][self.which_indices[index]]
    # dac_image = h5_file['dac_images'][self.which_indices[index]]
```

In [7]:

```
# check that some randoms are fine and augmented
from c_utils.utils_hour import plot_im_target, random_from
for i in range(3):    
#     im,target = random_from( MouseTrainLoader)
    im,target = next(iter( MouseTrainLoader))
    print(im.shape)
    print(im.dtype)
    print(torch.max(im))

    plot_im_target(im,target,5)
```

```
torch.Size([1, 3, 192, 320])
torch.float32
tensor(1.)
```

```
torch.Size([1, 3, 192, 320])
torch.float32
tensor(1.)
```

```
torch.Size([1, 3, 192, 320])
torch.float32
tensor(1.)
```

In [8]:

```
# check that some randoms from the valid are not augmented
from c_utils.utils_hour import plot_im_target, random_from
for _ in range(2):    
    im,target = random_from( MouseValidLoader)
#     im,target = next(iter( MouseTrainLoader))
    print(im.shape)
    plot_im_target(im,target,5)
```

```
28
torch.Size([1, 3, 192, 320])
```

```
38
torch.Size([1, 3, 192, 320])
```

In [9]:

```
# save an example for a figure!
from c_utils.utils_hour import plot_im_target_pseudo,specific_from,plot_and_dump_im_target_pseudo
# 1 is good
im,target = specific_from(MouseValidLoader,1)
plot_and_dump_im_target_pseudo(im,target,8)
```

```
1
```

```
/home/chrelli/git/3d_sandbox/share_code/analysis/c_utils/utils_hour.py:378: MatplotlibDeprecationWarning: Adding an axes using the same arguments as a previous axes currently reuses the earlier instance.  In a future version, a new instance will always be created and returned.  Meanwhile, this warning can be suppressed, and the future behavior ensured, by passing a unique label to each axes instance.
  plt.subplot(2,3,2)
/home/chrelli/git/3d_sandbox/share_code/analysis/c_utils/utils_hour.py:387: RuntimeWarning: invalid value encountered in true_divide
  plt.imshow( color_im/np.max(color_im) )
/home/chrelli/git/3d_sandbox/share_code/analysis/c_utils/utils_hour.py:389: RuntimeWarning: invalid value encountered in true_divide
  cv2.imwrite(figure_dump_folder+'/train/dump_pafs'+t+'.png',color_im[:,:,[2,1,0]]/np.max(color_im)*255)
```

In [10]:

```
# Look at a few examples with merged fields
from c_utils.utils_hour import plot_im_target_pseudo,random_from
for _ in range(3):
    im,target = random_from(MouseValidLoader)
    plot_im_target_pseudo(im,target,8)
```

```
37
```

```
/home/chrelli/git/3d_sandbox/share_code/analysis/c_utils/utils_hour.py:209: MatplotlibDeprecationWarning: Adding an axes using the same arguments as a previous axes currently reuses the earlier instance.  In a future version, a new instance will always be created and returned.  Meanwhile, this warning can be suppressed, and the future behavior ensured, by passing a unique label to each axes instance.
  plt.subplot(2,3,2)
```

```
5
```

```
/home/chrelli/git/3d_sandbox/share_code/analysis/c_utils/utils_hour.py:218: RuntimeWarning: invalid value encountered in true_divide
  plt.imshow( color_im/np.max(color_im) )
```

```
45
```

# SET up for learning¶

## Make the hourglass net¶

In [13]:

```
# OK, we now have a generator, let's print from it
print("Train: %d" %len(MouseTrainLoader))
print("Val: %d" %len(MouseValidLoader))
inputs, targets = next(iter(MouseTrainLoader))
print("Inputs: ", inputs.size())
print("Targets: ", targets.size())
print("Channels: %d" %inputs.shape[1] )
print("Classes: %d" %target.shape[1] )

    
# import the hourglass model and set up architecture
from architectures.hourglass import hg
global best_acc

model = hg(
    num_stacks=8,
    num_blocks=1,
    num_classes=11,
    num_feats=128,
    inplanes=64,
    init_stride=2,
)
    
model = torch.nn.DataParallel(model).cuda()
# set the loss
criterion = torch.nn.MSELoss(size_average=True).cuda()
```

```
Train: 576
Val: 50
Inputs:  torch.Size([1, 3, 192, 320])
Targets:  torch.Size([1, 11, 48, 80])
Channels: 3
Classes: 11
```

## Set the optimizer, LR schedule and the hourglass loss¶

In [14]:

```
# MAKE optimizer
optimizer = torch.optim.RMSprop(
    model.parameters(),
    lr= 0.00025,
    momentum=0,
    weight_decay=0,
)

# learning rate scheduler
scheduler = torch.optim.lr_scheduler.ReduceLROnPlateau(optimizer, verbose=True, patience=5)


# custom loss function for weighing
def weighted_mse_loss(input, target, weights):
    out = (input - target) ** 2
    out = out * weights.expand_as(out)
    loss = out.sum()
    return loss

# also load a summary_writer for tensorboard
def weighted_hourglass_loss(inputs,targets,output):
    # loss_weight = torch.ones((inputs.size(0), args.num_classes, 1, 1))
    loss_weight = torch.ones((inputs.size(0), targets.size(1), 1, 1))
    
    loss_weight.requires_grad = True
    loss_weight = loss_weight.cuda()

    # add all the loss maps together (8 hourglasses)
    loss = weighted_mse_loss(output[0], targets, weights=loss_weight)
    for j in range(1, len(output)):
        loss += weighted_mse_loss(output[j], targets, weights=loss_weight)

    return loss
```

## Define a training step¶

In [15]:

```
# make a function which will do a training step!
from torch.autograd import Variable
epoch = 0
def train(model, trn_loader, optimizer, epoch):
    model.train()
    epoch_loss = 0
    frame_loss = []
    NN = trn_loader.__len__()
    
    # trn_error = 0
    for idx, data in enumerate(trn_loader):
            
        ### output some progress ###
        if idx%100 == 0:
            print("im {} of {}...".format(idx,NN))
            
        ### PREPARE TENSORS ###
        
        # make right byte and shape (# remove the depth dimension)
        if data[0].shape[1] > 3:
            inputs = data[0][:,:3,:,:].float()
        else:
            inputs = data[0].float()
        targets = data[1].float()
               
        # send to cuda
        inputs = Variable(inputs.cuda())
        targets = Variable(targets.cuda(non_blocking=True))

        ### CALC LOSS ###
        
        # compute model output
        output = model(inputs)
        # get the score map to the cpu?  not now
        # score_map = output[-1].data.cpu()       
        
        # calculate the loss weights and send to cuda
        # it's a one pr class - maybe scale for the affinity fields?
        # scale by input weight, for example?
        # should call it class weight?
        loss = weighted_hourglass_loss(inputs,targets,output)
        
        ### DO BACKPROP ###
        optimizer.zero_grad()
        loss.backward()
        optimizer.step()

        epoch_loss += loss.item()
        frame_loss.append(loss.item())
        
    epoch_loss /= len(trn_loader)
    # trn_error /= len(trn_loader)
    return epoch_loss, frame_loss #, trn_error

# epoch_loss, frame_loss = train(model, TestTrainLoader, optimizer, epoch)
# frame_loss
```

## Define a validation step¶

In [16]:

```
# make a function which will do a training step!
from torch.autograd import Variable

def test(model, trn_loader, optimizer, epoch):
    model.eval()
    epoch_loss = 0
    frame_loss = []
    
    # trn_error = 0
    for idx, data in enumerate(trn_loader):
                
        ### PREPARE TENSORS ###
        
        # make right byte and shape (# remove the depth dimension)
        if data[0].shape[1] > 3:
            inputs = data[0][:,:3,:,:].float()
        else:
            inputs = data[0].float()
        targets = data[1].float()
               
        # send to cuda
        inputs = Variable(inputs.cuda())
        targets = Variable(targets.cuda(non_blocking=True))

        ### CALC LOSS W/O GRAD ###
        
        with torch.no_grad():
            # compute model output
            output = model(inputs)        
            loss = weighted_hourglass_loss(inputs,targets,output)
        
        epoch_loss += loss.item()
        frame_loss.append(loss.item())
        
    epoch_loss /= len(trn_loader)
    # trn_error /= len(trn_loader)
    return epoch_loss, frame_loss #, trn_error

# epoch_loss, frame_loss = test(model, TestTrainLoader, optimizer, epoch)
# frame_loss
```

## Define a saving func (dump weights) and a plotting func (dump figure)¶

In [17]:

```
# A HELPER FUNCTION WHICH SAVES THE STATE OF THE NETWORK, maybe every 10 epochs or smth?
import os
import sys
import math
import string
import random
import shutil

WEIGHTS_PATH = '192x320_weights/'

def save_weights(model, epoch, loss):
    time_string = time.strftime('%Y-%m-%d_%H-%M-%S',time.localtime())
    weights_fname = 'singlecore_weights_epoch_%d_loss_%.3f_'% (epoch, loss) +time_string+'.pth' 
    weights_fpath = os.path.join(WEIGHTS_PATH, weights_fname)
    torch.save(model.state_dict(), weights_fpath)
    shutil.copyfile(weights_fpath, WEIGHTS_PATH+'singlecore_latest.th')
```

In [18]:

```
from c_utils.utils_hour import plot_ito_pseudo

def dump_inspection(model, trn_loader, optimizer, epoch,WEIGHTS_PATH,filetag=''):
    # def dump_inspection(model, trn_loader, epoch, EIGHTS_PATH):
    model.eval()
    epoch_loss = 0
    frame_loss = []

    idx, data = next(iter(enumerate( trn_loader)))

    ### PREPARE TENSORS ###

    # make right byte and shape (# remove the depth dimension)
    if data[0].shape[1] > 3:
        inputs = data[0][:,:3,:,:].float()
    else:
        inputs = data[0].float()
    targets = data[1].float()

    # send to cuda
    inputs = Variable(inputs.cuda())
    targets = Variable(targets.cuda(non_blocking=True))

    ### CALC LOSS W/O GRAD ###
    with torch.no_grad():
        # compute model output
        output = model(inputs)        
        loss = weighted_hourglass_loss(inputs,targets,output)

    Fig1 = plot_ito_pseudo(inputs,targets,output,size = 10)

    time_string = time.strftime('%Y-%m-%d_%H-%M-%S',time.localtime())
    fig_fname = 'singlecore_'+filetag+'_'+time_string+'_epoch_%d_loss_%.3f_'% (epoch, loss) +'.png' 
    fig_fpath = os.path.join(WEIGHTS_PATH, fig_fname)

    plt.savefig(fig_fpath)
    plt.close(Fig1)    
    
# dump_inspection(model, trn_loader, optimizer, epoch,WEIGHTS_PATH)
```

# TRAINING LOOP¶

In [19]:

```
# run actual training loop

from torch.utils.tensorboard import SummaryWriter

writer = SummaryWriter()

train_loader = MouseTrainLoader
val_loader = MouseValidLoader

N_EPOCHS = 120 
START_EPOCH = 0

for epoch in range(START_EPOCH, START_EPOCH+N_EPOCHS):
    since = time.time()

    ### Train ###
    trn_loss, trn_frame_loss = train(model, train_loader, optimizer, epoch)
    print('Epoch {:d}\nTrain - Loss: {:.4f}'.format(epoch, trn_loss))    
    time_elapsed = time.time() - since  
    print('Train Time {:.0f}m {:.0f}s'.format( time_elapsed // 60, time_elapsed % 60) )
    
    ### Test ###    
    val_loss, val_frame_loss = test(model, val_loader, optimizer, epoch)
    print('Epoch {:d}\nValidation - Loss: {:.4f}'.format(epoch, val_loss))    
    time_elapsed = time.time() - since  
    print('Train Time {:.0f}m {:.0f}s'.format( time_elapsed // 60, time_elapsed % 60) )
    
   
    ### Checkpoint ###  
    if epoch % 5 == 0:
        save_weights(model, epoch, val_loss)

    ### write to and save a figure ###
        
    writer.add_scalar('trn_loss', trn_loss, global_step=epoch)
    writer.add_scalar('val_loss', val_loss, global_step=epoch)
            
    for ll in trn_frame_loss:
        writer.add_scalar('trn_frame_loss', ll)
    
    for ll in val_frame_loss:
        writer.add_scalar('val_frame_loss', ll)

    
    dump_inspection(model, train_loader, optimizer, epoch,WEIGHTS_PATH,filetag='train')
    dump_inspection(model, val_loader, optimizer, epoch,WEIGHTS_PATH,filetag='valid')
    
    ### Adjust learning rate ###
    scheduler.step(val_loss)
    
    if epoch == 90:
        for param_group in optimizer.param_groups:
            param_group['lr'] = 1e-4
    
save_weights(model, epoch, val_loss)    
writer.close()
```

```
im 0 of 576...
im 100 of 576...
im 200 of 576...
im 300 of 576...
im 400 of 576...
im 500 of 576...
Epoch 0
Train - Loss: 1146.5784
Train Time 2m 55s
Epoch 0
Validation - Loss: 566.4172
Train Time 2m 59s
im 0 of 576...
im 100 of 576...
im 200 of 576...
im 300 of 576...
im 400 of 576...
im 500 of 576...
Epoch 1
Train - Loss: 458.8006
Train Time 2m 56s
Epoch 1
Validation - Loss: 803.0840
Train Time 2m 59s
im 0 of 576...
im 100 of 576...
im 200 of 576...
im 300 of 576...
im 400 of 576...
im 500 of 576...
Epoch 2
Train - Loss: 388.6464
Train Time 2m 56s
Epoch 2
Validation - Loss: 800.9391
Train Time 2m 59s
im 0 of 576...
im 100 of 576...
im 200 of 576...
im 300 of 576...
im 400 of 576...
im 500 of 576...
Epoch 3
Train - Loss: 361.9273
Train Time 2m 53s
Epoch 3
Validation - Loss: 313.0572
Train Time 2m 56s
im 0 of 576...
im 100 of 576...
im 200 of 576...
im 300 of 576...
im 400 of 576...
im 500 of 576...
Epoch 4
Train - Loss: 333.0115
Train Time 2m 56s
Epoch 4
Validation - Loss: 3954.7535
Train Time 2m 59s
im 0 of 576...
im 100 of 576...
im 200 of 576...
im 300 of 576...
im 400 of 576...
im 500 of 576...
Epoch 5
Train - Loss: 311.4291
Train Time 2m 57s
Epoch 5
Validation - Loss: 1166.1981
Train Time 3m 1s
im 0 of 576...
im 100 of 576...
im 200 of 576...
im 300 of 576...
im 400 of 576...
im 500 of 576...
Epoch 6
Train - Loss: 300.0168
Train Time 2m 57s
Epoch 6
Validation - Loss: 4122.1966
Train Time 3m 0s
im 0 of 576...
im 100 of 576...
im 200 of 576...
im 300 of 576...
im 400 of 576...
im 500 of 576...
Epoch 7
Train - Loss: 285.7114
Train Time 2m 57s
Epoch 7
Validation - Loss: 741.1418
Train Time 2m 60s
im 0 of 576...
im 100 of 576...
im 200 of 576...
im 300 of 576...
im 400 of 576...
im 500 of 576...
Epoch 8
Train - Loss: 279.1951
Train Time 2m 57s
Epoch 8
Validation - Loss: 412.7393
Train Time 3m 0s
im 0 of 576...
im 100 of 576...
im 200 of 576...
im 300 of 576...
im 400 of 576...
im 500 of 576...
Epoch 9
Train - Loss: 268.7975
Train Time 2m 55s
Epoch 9
Validation - Loss: 338.0809
Train Time 2m 59s
Epoch    10: reducing learning rate of group 0 to 2.5000e-05.
```
