# Supplementary material for: Automatic mapping of multiplexed social receptive fields by deep learning and GPU-accelerated 3D videography
Source: Nat Commun. 2022 Feb 1;13:593. doi: 10.1038/s41467-022-28153-7 (PMC8807631; doi:10.1038/s41467-022-28153-7)
Supplement: Supplementary file 9 — Supplementary Software [file 41467_2022_28153_MOESM9_ESM.zip › ebbesen_froemke_2021_code/analysis/017_Plot_example_error_and_artifact.html]

017\_Plot\_example\_error\_and\_artifact


In [1]:

```
# IDEA: Add neck to the posture map?
from IPython import get_ipython

# QT for movable plots
%load_ext autoreload
%autoreload 2

import time, os, sys, shutil
from utils.fitting_utils import *

# for math and plotting
import pandas as pd
import numpy as np
import scipy as sp
import matplotlib.pyplot as plt
%matplotlib notebook
# %matplotlib inline

# %matplotlib widget
# %matplotlib qt

from itertools import compress # for list selection with logical
from tqdm import tqdm

from multiprocessing import Process

# ALLSO JIT STUFF
from numba import jit, njit

# and pytorch
import torch

import sys, os, pickle
# import cv2
from colour import Color
import h5py
from tqdm import tqdm, tqdm_notebook
import glob
import itertools
```

In [2]:

```
from utils.analysis_tools import adjust_spines,cmpl
```

# Load tracked data and plot a frame¶

In [3]:

```
#load the tracked data!
data_folder = 'example_data/tracking/'

with open(data_folder +'tracked_behavior.pkl', 'rb') as f:
    tracked_behavior = pickle.load(f)
print(tracked_behavior.keys())

# load ALL the frames as jagged lines 
with h5py.File(data_folder+'/pre_processed_frames.hdf5', mode='r') as hdf5_file:
    print(hdf5_file.keys())
    print(len(hdf5_file['dataset']))
    jagged_lines = hdf5_file['dataset'][...]
```

```
dict_keys(['var', 'ivar', 'body_constants', 'start_frame', 'end_frame', 'tracking_holder', 'guessing_holder', 'data_folder'])
<KeysViewHDF5 ['dataset']>
74962
```

In [4]:

```
from utils.cuda_tracking_utils import unpack_from_jagged, cheap4d
# kill first 6 secs of the frames (delay is ~180)
start_frame = 30*60
pos, pos_weights, keyp, pkeyp, ikeyp = unpack_from_jagged(jagged_lines[start_frame])
print(ikeyp)
print(pos.shape)

cheap4d(pos,keyp,ikeyp)
# fig = plt.gcf()
# plt.title("N positions is {}".format(pos.shape))
```

```
[0 1 1 2 3 0 1 1 1 2 2 0 2 0 1 1 1 2 3]
(2986, 3)
```

In [5]:

```
print(tracked_behavior['tracking_holder'].shape)
print(tracked_behavior['var'])
print(tracked_behavior['ivar'])
print(tracked_behavior.keys())
```

```
(18, 73361)
['b', 'c', 's', 'psi', 'theta', 'phi', 'x', 'y', 'z', 'b', 'c', 's', 'theta', 'phi', 'x', 'y', 'z']
['b0', 'c0', 's0', 'psi0', 'theta0', 'phi0', 'x0', 'y0', 'z0', 'b1', 'c1', 's1', 'theta1', 'phi1', 'x1', 'y1', 'z1']
dict_keys(['var', 'ivar', 'body_constants', 'start_frame', 'end_frame', 'tracking_holder', 'guessing_holder', 'data_folder'])
```

In [6]:

```
# Make the plotter and smooth the data
from utils.analysis_tools import particles_to_body_supports_cuda,VideoPlotMachine
Plotter = VideoPlotMachine(tracked_behavior,jagged_lines)
Plotter.better_smoothing()
```

```
100%|██████████| 73361/73361 [00:17<00:00, 4292.41it/s]
100%|██████████| 73361/73361 [00:16<00:00, 4367.98it/s]
100%|██████████| 73361/73361 [00:17<00:00, 4309.04it/s]
100%|██████████| 73361/73361 [00:16<00:00, 4318.86it/s]
100%|██████████| 73361/73361 [00:17<00:00, 4187.66it/s]
100%|██████████| 73361/73361 [00:17<00:00, 4128.77it/s]
100%|██████████| 73361/73361 [00:14<00:00, 4904.66it/s]
100%|██████████| 73361/73361 [00:17<00:00, 4118.57it/s]
100%|██████████| 73361/73361 [00:17<00:00, 4140.09it/s]
100%|██████████| 73361/73361 [00:17<00:00, 4137.51it/s]
100%|██████████| 73361/73361 [00:17<00:00, 4134.83it/s]
100%|██████████| 73361/73361 [00:17<00:00, 4129.41it/s]
100%|██████████| 73361/73361 [00:17<00:00, 4129.62it/s]
100%|██████████| 73361/73361 [00:14<00:00, 4966.28it/s]
```

In [33]:

```
# Check that the plotter is working
%matplotlib inline
frame = 45000
Plotter.make_me(frame,cloud = True,skel='smooth',ellip='smooth',
             trace='smooth',view_override = [55.0,90.+46])
```

# Dump 500 frames and manually inspect for errors¶

In [ ]:

```
# Plot the examples
plt.close('all')
# allow burn in 
n_check = 500
check_frames = np.random.randint(low=3600,high=Plotter.n_frames,size = n_check)
check_frames = np.round( np.linspace(3600,Plotter.n_frames,n_check) ).astype('int')

for i in tqdm(check_frames):    
    Plotter.make_me(i,cloud = True,skel='smooth',ellip='smooth',
             trace='smooth',view_override = [55.0,90.+46],savepath = 'figs/error_check/check_frame_{}.png'.format(i))
```

In [15]:

```
# counted one mistake in 500 frames, make waffle plot
plot_folder = 'figs'

n_test = 500
n_error = 1

from pywaffle import Waffle
fig = plt.figure(
    FigureClass=Waffle, 
    rows=25, 
    values=[n_test-n_error, n_error],
    figsize=(4, 4),  # figsize is a parameter of matplotlib.pyplot.figure
    colors=(cmpl[1], cmpl[3]))
plt.savefig(plot_folder+'/error_waffle.png')
plt.show()
```

# Make some supplementary plots to show errors¶

In [228]:

```
# Calculate the pointcloud loss pr frame, and -- also -- the total final loss for all #todo: also save this during tracking - stupid not to
from utils.cuda_tracking_utils_weights_for_figures import particles_to_distance_cuda, torch_device

self = Plotter
pc_loss_holder = []
pc_loss_frames = []
# for i_frame in tqdm(range(20000)):
for i_frame in tqdm(range(self.tracking_holder.shape[1])):
    # get the frame index in the tracked data, bit annoying, but useful if only a portion is tracked
    # frame is the raw data
    # i_frame is the tracked data
    frame = i_frame+self.start_frame
    
    # get the particle from the tracked data
    part = torch.tensor(self.tracking_holder[:,i_frame],dtype = torch.float32).unsqueeze(0).to(torch_device)
    
    # and unpack the positions from the jagged lines
    pos, pos_weights, keyp, pkeyp, ikeyp = unpack_from_jagged(self.jagged_lines[frame])
    pos = torch.tensor(pos,dtype = torch.float32).to(torch_device)

    dist0,_,self.body_support_0 = particles_to_distance_cuda(part[:,:9],pos[::5,:],implant = True)
    dist1,_,self.body_support_1 = particles_to_distance_cuda(part[:,9:],pos[::5,:])
    r = torch.min(dist0,dist1)
    r = torch.clamp(r,0,.03)          

    pc_loss_holder.append(r.cpu().numpy().ravel())
    pc_loss_frames.append(frame)
```

```
100%|██████████| 73361/73361 [06:00<00:00, 203.59it/s]
```

In [261]:

```
# plot example frames around the error
error_frame = 16741

# error_frame = 20700+self.start_frame

n_s = 5
frame_step = 30
plot_frames = np.arange(-n_s+1,n_s+1) * frame_step+ error_frame
print(plot_frames)
for i in tqdm(plot_frames):  
    i = int(i)
#     Plotter.make_me(i,cloud = True,skel='smooth',ellip='smooth',
#              trace='smooth',view_override = [55.0,90.+20],savepath = plot_folder+'/error_example_flip_over_{}.png'.format(i))
    Plotter.make_me(i,cloud = True,skel='smooth',ellip='smooth',
             trace='smooth',view_override = [55.0,90.+20],savepath = plot_folder+'/error_example_flip_over_{}.png'.format(i))
```

```
  0%|          | 0/10 [00:00<?, ?it/s]
```

```
[22180 22210 22240 22270 22300 22330 22360 22390 22420 22450]
```

```
 10%|█         | 1/10 [00:00<00:03,  2.49it/s]
```

```
 20%|██        | 2/10 [00:00<00:03,  2.48it/s]
```

```
 30%|███       | 3/10 [00:01<00:02,  2.44it/s]
```

```
 40%|████      | 4/10 [00:01<00:02,  2.35it/s]
```

```
 50%|█████     | 5/10 [00:02<00:02,  2.30it/s]
```

```
 60%|██████    | 6/10 [00:02<00:01,  2.26it/s]
```

```
 70%|███████   | 7/10 [00:03<00:01,  2.26it/s]
```

```
 80%|████████  | 8/10 [00:03<00:00,  2.28it/s]
```

```
 90%|█████████ | 9/10 [00:03<00:00,  2.36it/s]
```

```
100%|██████████| 10/10 [00:04<00:00,  2.35it/s]
```

In [229]:

```
# Plot the flip over and the loss around the error frame
loss = pc_loss_holder
loss_frame = pc_loss_frames
mean_loss = [np.median(l) for l in loss]
n_bad = [np.sum(l==0.03) for l in loss]
```

In [256]:

```
def adjust_spines2(ax, spines):
    for loc, spine in ax.spines.items():
        if loc in spines:
            spine.set_position(('outward', 10))  # outward by 10 points
#             spine.set_smart_bounds(True)
        else:
            spine.set_color('none')  # don't draw spine

    # turn off ticks where there is no spine
    if 'left' in spines:
        ax.yaxis.set_ticks_position('left')
    else:
        # no yaxis ticks
        ax.yaxis.set_ticks([])

    if 'bottom' in spines:
        ax.xaxis.set_ticks_position('bottom')
    else:
        # no xaxis ticks
        ax.xaxis.set_ticks([])        


buff = 500000
plt.figure(figsize=(8,4))

# x_min = plot_frames[0] - self.start_frame - buff
# x_max = plot_frames[-1] - self.start_frame + buff
x_min = plot_frames[0] - buff
x_max = plot_frames[-1] + buff


plt.subplot(2,1,1)

plt.plot(loss_frame,mean_loss,'-',c=cmpl[3])
# plt.xlim(x_min,x_max)
plt.gca().set_ylim(bottom=0)
plt.axis('off')
plt.ylim([0,.03])
plt.xticks([])
plt.yticks([0,.03])
adjust_spines(plt.gca(),'left')
plt.subplot(2,1,2)

# plt.plot(loss_frame,n_bad,'-',c=cmpl[3])
plt.plot(loss_frame,n_bad,'-',c=cmpl[3],markersize = mz)

# plt.xlim(x_min,x_max)
plt.gca().set_ylim(bottom=0)
plt.axis('off')
plt.xticks([])
plt.yticks([0,400])
adjust_spines(plt.gca(),'left')

plt.subplots_adjust(hspace = 0)    


plt.savefig(plot_folder+'/error_wide.pdf',transparent = True)    
plt.show()


buff = 0
plt.figure(figsize=(3.5,4))

# x_min = plot_frames[0] - self.start_frame - buff
# x_max = plot_frames[-1] - self.start_frame + buff
x_min = plot_frames[0] - buff
x_max = plot_frames[-1] + buff


plt.subplot(2,1,1)
mz = 1.5

plt.plot(loss_frame,mean_loss,'.',c=cmpl[3],markersize = mz)
plt.xlim(x_min,x_max)
plt.gca().set_ylim(bottom=0)
for f in plot_frames:
    plt.axvline(f,c=cmpl[1])
plt.axis('off')
plt.plot(loss_frame,mean_loss,'.',c=cmpl[3],markersize = mz)
plt.ylim([0,.03])

plt.subplot(2,1,2)
plt.plot(loss_frame,n_bad,'.',c=cmpl[3],markersize = mz)
plt.xlim(x_min,x_max)
plt.gca().set_ylim(bottom=0)
plt.axis('off')

for f in plot_frames:
    plt.axvline(f,c=cmpl[1])

plt.plot(loss_frame,n_bad,'.',c=cmpl[3],markersize = mz)

plt.subplots_adjust(hspace = 0)    
plt.savefig(plot_folder+'/error_zoom.pdf',transparent = True)    
plt.show()
```

In [262]:

```
error_frame = 20700+self.start_frame

n_s = 5
frame_step = 30
plot_frames = np.arange(-n_s+1,n_s+1) * frame_step+ error_frame

buff = 0
plt.figure(figsize=(3.5,4))

# x_min = plot_frames[0] - self.start_frame - buff
# x_max = plot_frames[-1] - self.start_frame + buff
x_min = plot_frames[0] - buff
x_max = plot_frames[-1] + buff


plt.subplot(2,1,1)
mz = 1.5

plt.plot(loss_frame,mean_loss,'.',c=cmpl[3],markersize = mz)
plt.xlim(x_min,x_max)
plt.gca().set_ylim(bottom=0)
for f in plot_frames:
    plt.axvline(f,c=cmpl[1])
plt.axis('off')
plt.plot(loss_frame,mean_loss,'.',c=cmpl[3],markersize = mz)
plt.ylim([0,.03])

plt.subplot(2,1,2)
plt.plot(loss_frame,n_bad,'.',c=cmpl[3],markersize = mz)
plt.xlim(x_min,x_max)
plt.gca().set_ylim(bottom=0)
plt.axis('off')

for f in plot_frames:
    plt.axvline(f,c=cmpl[1])

plt.plot(loss_frame,n_bad,'.',c=cmpl[3],markersize = mz)

plt.subplots_adjust(hspace = 0)    
plt.savefig(plot_folder+'/error_zoom2.pdf',transparent = True)    
plt.show()
```

In [ ]:
