# Supplementary material for: Automatic mapping of multiplexed social receptive fields by deep learning and GPU-accelerated 3D videography
Source: Nat Commun. 2022 Feb 1;13:593. doi: 10.1038/s41467-022-28153-7 (PMC8807631; doi:10.1038/s41467-022-28153-7)
Supplement: Supplementary file 9 — Supplementary Software [file 41467_2022_28153_MOESM9_ESM.zip › ebbesen_froemke_2021_code/analysis/016_Save_a_10s_sequence_of_images.html]

016\_Save\_a\_10s\_sequence\_of\_images


In [1]:

```
# IDEA: Add neck to the posture map?
from IPython import get_ipython

# QT for movable plots
%load_ext autoreload
%autoreload 2

import time, os, sys, shutil
from utils.fitting_utils import *

# for math and plotting
import pandas as pd
import numpy as np
import scipy as sp
import matplotlib.pyplot as plt
%matplotlib notebook
# %matplotlib inline

# %matplotlib widget
# %matplotlib qt

from itertools import compress # for list selection with logical
from tqdm import tqdm

from multiprocessing import Process

# ALLSO JIT STUFF
from numba import jit, njit

# and pytorch
import torch

import sys, os, pickle
# import cv2
from colour import Color
import h5py
from tqdm import tqdm, tqdm_notebook
import glob
import itertools
```

In [2]:

```
from utils.analysis_tools import adjust_spines,cmpl
```

In [3]:

```
# Load tracked data and plot a frame
```

In [4]:

```
#load the tracked data!
data_folder = 'example_data/tracking/'

with open(data_folder +'tracked_behavior.pkl', 'rb') as f:
    tracked_behavior = pickle.load(f)
print(tracked_behavior.keys())

# load ALL the frames as jagged lines 
with h5py.File(data_folder+'/pre_processed_frames.hdf5', mode='r') as hdf5_file:
    print(hdf5_file.keys())
    print(len(hdf5_file['dataset']))
    jagged_lines = hdf5_file['dataset'][...]
```

```
dict_keys(['var', 'ivar', 'body_constants', 'start_frame', 'end_frame', 'tracking_holder', 'guessing_holder', 'data_folder'])
<KeysViewHDF5 ['dataset']>
74962
```

In [5]:

```
from utils.cuda_tracking_utils import unpack_from_jagged, cheap4d
# kill first 6 secs of the frames (delay is ~180)
start_frame = 30*60
pos, pos_weights, keyp, pkeyp, ikeyp = unpack_from_jagged(jagged_lines[start_frame])
print(ikeyp)
print(pos.shape)

cheap4d(pos,keyp,ikeyp)
# fig = plt.gcf()
# plt.title("N positions is {}".format(pos.shape))
```

```
[0 1 1 2 3 0 1 1 1 2 2 0 2 0 1 1 1 2 3]
(2986, 3)
```

```
findfont: Font family ['cursive'] not found. Falling back to DejaVu Sans.
```

In [6]:

```
print(tracked_behavior['tracking_holder'].shape)
print(tracked_behavior['var'])
print(tracked_behavior['ivar'])
print(tracked_behavior.keys())
```

```
(18, 73361)
['b', 'c', 's', 'psi', 'theta', 'phi', 'x', 'y', 'z', 'b', 'c', 's', 'theta', 'phi', 'x', 'y', 'z']
['b0', 'c0', 's0', 'psi0', 'theta0', 'phi0', 'x0', 'y0', 'z0', 'b1', 'c1', 's1', 'theta1', 'phi1', 'x1', 'y1', 'z1']
dict_keys(['var', 'ivar', 'body_constants', 'start_frame', 'end_frame', 'tracking_holder', 'guessing_holder', 'data_folder'])
```

In [39]:

```
# Make the plotter and smooth the data
from utils.analysis_tools import particles_to_body_supports_cuda,VideoPlotMachine
Plotter = VideoPlotMachine(tracked_behavior,jagged_lines)
Plotter.better_smoothing()
```

```
[autoreload of utils.analysis_tools failed: Traceback (most recent call last):
  File "/home/chrelli/anaconda2/envs/mousepose/lib/python3.6/site-packages/IPython/extensions/autoreload.py", line 245, in check
    superreload(m, reload, self.old_objects)
  File "/home/chrelli/anaconda2/envs/mousepose/lib/python3.6/site-packages/IPython/extensions/autoreload.py", line 450, in superreload
    update_generic(old_obj, new_obj)
  File "/home/chrelli/anaconda2/envs/mousepose/lib/python3.6/site-packages/IPython/extensions/autoreload.py", line 387, in update_generic
    update(a, b)
  File "/home/chrelli/anaconda2/envs/mousepose/lib/python3.6/site-packages/IPython/extensions/autoreload.py", line 357, in update_class
    update_instances(old, new)
  File "/home/chrelli/anaconda2/envs/mousepose/lib/python3.6/site-packages/IPython/extensions/autoreload.py", line 312, in update_instances
    update_instances(old, new, obj.__dict__, visited)
  File "/home/chrelli/anaconda2/envs/mousepose/lib/python3.6/site-packages/IPython/extensions/autoreload.py", line 312, in update_instances
    update_instances(old, new, obj.__dict__, visited)
  File "/home/chrelli/anaconda2/envs/mousepose/lib/python3.6/site-packages/IPython/extensions/autoreload.py", line 317, in update_instances
    update_instances(old, new, obj, visited)
  File "/home/chrelli/anaconda2/envs/mousepose/lib/python3.6/site-packages/IPython/extensions/autoreload.py", line 317, in update_instances
    update_instances(old, new, obj, visited)
  File "/home/chrelli/anaconda2/envs/mousepose/lib/python3.6/site-packages/IPython/extensions/autoreload.py", line 312, in update_instances
    update_instances(old, new, obj.__dict__, visited)
  File "/home/chrelli/anaconda2/envs/mousepose/lib/python3.6/site-packages/IPython/extensions/autoreload.py", line 317, in update_instances
    update_instances(old, new, obj, visited)
AttributeError: 'dict' object has no attribute 'iterkeys'
]
100%|██████████| 73361/73361 [00:20<00:00, 3629.09it/s]
100%|██████████| 73361/73361 [00:20<00:00, 3661.78it/s]
100%|██████████| 73361/73361 [00:20<00:00, 3622.67it/s]
100%|██████████| 73361/73361 [00:20<00:00, 3619.58it/s]
100%|██████████| 73361/73361 [00:20<00:00, 3627.39it/s]
100%|██████████| 73361/73361 [00:19<00:00, 3679.96it/s]
100%|██████████| 73361/73361 [00:10<00:00, 7083.68it/s]
100%|██████████| 73361/73361 [00:20<00:00, 3661.64it/s]
100%|██████████| 73361/73361 [00:20<00:00, 3663.73it/s]
100%|██████████| 73361/73361 [00:20<00:00, 3645.11it/s]
100%|██████████| 73361/73361 [00:19<00:00, 3670.66it/s]
100%|██████████| 73361/73361 [00:20<00:00, 3634.96it/s]
100%|██████████| 73361/73361 [00:20<00:00, 3612.04it/s]
100%|██████████| 73361/73361 [00:10<00:00, 6958.82it/s]
```

In [44]:

```
# Save the three example social behaviors for Fig 6
%matplotlib inline
view = [50.0-0,-15]
for i in np.arange(10):
    frame = (30+i)*60
    path = '/home/chrelli/git/3d_sandbox/mouseposev0p2/figure_raw_pics/figure_5/kalman_examples/'+str(frame)+'.pdf'
    Plotter.make_me(frame,cloud = True,skel='smooth',ellip='smooth',
             trace='smooth',trace_clip=30*60,view_override = view,savepath=path)
```

In [ ]:
