# Supplementary material for: Automatic mapping of multiplexed social receptive fields by deep learning and GPU-accelerated 3D videography
Source: Nat Commun. 2022 Feb 1;13:593. doi: 10.1038/s41467-022-28153-7 (PMC8807631; doi:10.1038/s41467-022-28153-7)
Supplement: Supplementary file 9 — Supplementary Software [file 41467_2022_28153_MOESM9_ESM.zip › ebbesen_froemke_2021_code/analysis/008_Plot_tracked_paramters.html]

008\_Plot\_tracked\_paramters


In [1]:

```
# IDEA: Add neck to the posture map?
from IPython import get_ipython

# QT for movable plots
%load_ext autoreload
%autoreload 2

import time, os, sys, shutil
from utils.fitting_utils import *

# for math and plotting
import pandas as pd
import numpy as np
import scipy as sp
import matplotlib.pyplot as plt
# %matplotlib notebook
# %matplotlib inline

# %matplotlib widget
# %matplotlib qt

from itertools import compress # for list selection with logical
from tqdm import tqdm

from multiprocessing import Process

# ALLSO JIT STUFF
from numba import jit, njit

# and pytorch
import torch

import sys, os, pickle
# import cv2
from colour import Color
import h5py
from tqdm import tqdm, tqdm_notebook
import glob
import itertools
```

# Load data, set up plot style¶

In [2]:

```
# setup for pretty plotting

import matplotlib

# Say, "the default sans-serif font is COMIC SANS"
matplotlib.rcParams['font.sans-serif'] = "Liberation Sans"
# Then, "ALWAYS use sans-serif fonts"
matplotlib.rcParams['font.family'] = "sans-serif"

matplotlib.rc('font', family='sans-serif') 
matplotlib.rc('text', usetex='false') 
matplotlib.rcParams.update({'font.size': 13})

from palettable.cmocean.sequential import Algae_6
cmpl = Algae_6.mpl_colors

def adjust_spines(ax, spines):
    for loc, spine in ax.spines.items():
        if loc in spines:
            spine.set_position(('outward', 10))  # outward by 10 points
            spine.set_smart_bounds(True)
        else:
            spine.set_color('none')  # don't draw spine

    # turn off ticks where there is no spine
    if 'left' in spines:
        ax.yaxis.set_ticks_position('left')
    else:
        # no yaxis ticks
        ax.yaxis.set_ticks([])

    if 'bottom' in spines:
        ax.xaxis.set_ticks_position('bottom')
    else:
        # no xaxis ticks
        ax.xaxis.set_ticks([])
```

In [4]:

```
#load the tracked data!
data_folder = 'example_data/tracking/'

with open(data_folder +'tracked_behavior.pkl', 'rb') as f:
    tracked_behavior = pickle.load(f)
print(tracked_behavior.keys())

# load ALL the frames as jagged lines 
with h5py.File(data_folder+'pre_processed_frames.hdf5', mode='r') as hdf5_file:
    print(hdf5_file.keys())
    print(len(hdf5_file['dataset']))
    jagged_lines = hdf5_file['dataset'][...]
```

```
dict_keys(['var', 'ivar', 'body_constants', 'start_frame', 'end_frame', 'tracking_holder', 'guessing_holder', 'data_folder'])
<KeysViewHDF5 ['dataset']>
74962
```

# Have a look¶

In [5]:

```
from utils.cuda_tracking_utils import unpack_from_jagged, cheap4d
# kill first 6 secs of the frames (delay is ~180)
start_frame = 30*60
pos, pos_weights, keyp, pkeyp, ikeyp = unpack_from_jagged(jagged_lines[start_frame])
print(ikeyp)
print(pos.shape)

cheap4d(pos,keyp,ikeyp)
# fig = plt.gcf()
# plt.title("N positions is {}".format(pos.shape))
```

```
[0 1 1 2 3 0 1 1 1 2 2 0 2 0 1 1 1 2 3]
(2986, 3)
```

In [6]:

```
print(tracked_behavior['tracking_holder'].shape)
print(tracked_behavior['var'])
print(tracked_behavior['ivar'])
print(tracked_behavior.keys())
```

```
(18, 73361)
['b', 'c', 's', 'psi', 'theta', 'phi', 'x', 'y', 'z', 'b', 'c', 's', 'theta', 'phi', 'x', 'y', 'z']
['b0', 'c0', 's0', 'psi0', 'theta0', 'phi0', 'x0', 'y0', 'z0', 'b1', 'c1', 's1', 'theta1', 'phi1', 'x1', 'y1', 'z1']
dict_keys(['var', 'ivar', 'body_constants', 'start_frame', 'end_frame', 'tracking_holder', 'guessing_holder', 'data_folder'])
```

# Plot a zoomed chunk for a main figure¶

In [9]:

```
import matplotlib

# Say, "the default sans-serif font is COMIC SANS"
matplotlib.rcParams['font.sans-serif'] = "Liberation Sans"
# Then, "ALWAYS use sans-serif fonts"
matplotlib.rcParams['font.family'] = "sans-serif"

matplotlib.rc('font', family='sans-serif') 
matplotlib.rc('text', usetex='false') 
matplotlib.rcParams.update({'font.size': 13})

from palettable.cmocean.sequential import Algae_6
cmpl = Algae_6.mpl_colors


sc=2
plt.figure(figsize = (5,9.5) )
n_vars = len(tracked_behavior['var'])
n_frames = tracked_behavior['tracking_holder'].shape[1]
start_frame = 200
frame_window = 600

scale_bar = [1,1,.1,1,1,1,.01,.01,.01,1,1,.1,1,1,.1,.1,.1]
units = ["rad","rad","a.u.",'rad','rad','rad',"m","m","m","rad","rad","a.u.",'rad','rad',"m","m","m"]
latex_vars = ['β', 'γ', 's', 'ψ', 'θ', 'φ', 'x', 'y', 'z', 'β', 'γ', 's', 'θ', 'φ', 'x', 'y', 'z']

for i in range(n_vars):
    plt.subplot(n_vars,1,1+i)
    
    plt.plot(tracked_behavior['tracking_holder'][i,start_frame:(start_frame+frame_window)],c=cmpl[1])
    plt.plot(tracked_behavior['guessing_holder'][i,start_frame:(start_frame+frame_window)],c=cmpl[3])
    
    ax = plt.gca()
    plt.gca().spines['top'].set_visible(False)
    plt.gca().spines['right'].set_visible(False)
    plt.gca().spines['left'].set_visible(False)
    
    if i < (n_vars-1):

        pass
        plt.gca().spines['bottom'].set_visible(False)
        plt.xticks([])

#         adjust_spines(ax,[])
    else:
        pass
#         adjust_spines(ax,['bottom'])

        plt.gca().spines['bottom'].set_bounds(0,600)
        plt.xticks([0,600])
        
    if i < 9:
        ax.set_facecolor('k')
        ax.patch.set_alpha(0.13)
    else:
        ax.set_facecolor('peru')      
        ax.patch.set_alpha(0.2)

        
#         adjust_spines(ax,['bottom','left'])
    plt.yticks([])
#     plt.ylabel(tracked_behavior['var'][i])
    plt.ylabel(latex_vars[i])
    plt.xlim([0,frame_window+10])
    
    ax = plt.gca()
    
    ylim=ax.get_ylim()
    xlim=ax.get_xlim()
    plt.plot( -2+np.array([1,1])*xlim[1],ylim[0]+np.array([0,1])*scale_bar[i],'-k' )
    plt.text(xlim[1],ylim[0]," "+str(scale_bar[i])+' '+units[i] )
    
plt.subplots_adjust(left=None, bottom=None, right=None, top=None, wspace=None, hspace=0.)
plt.xlabel('Frames',labelpad =-10)        
# plt.tight_layout()
# plt.savefig('figure_raw_pics/figure_5/fitting_cartoon/tracked_zoom_pretty.pdf',transparent=False,
#             bbox_inches = 'tight',pad_inches =1)  

plt.show()
```

# Plot the whole recording (params and body points) for a supplementary figure¶

In [12]:

```
sc=2
plt.figure(figsize = (20,8) )
n_vars = len(tracked_behavior['var'])
start_frame = 0
frame_window = n_frames
do_wrapping = True

scale_bar = [1,1,.1,1,1,1,.01,.01,.01,1,1,.1,1,1,.1,.1,.1]
units = ["rad","rad","a.u.",'rad','rad','rad',"m","m","m","rad","rad","a.u.",'rad','rad',"m","m","m"]
latex_vars = ['β', 'γ', 's', 'ψ', 'θ', 'φ', 'x', 'y', 'z', 'β', 'γ', 's', 'θ', 'φ', 'x', 'y', 'z']

for i in range(n_vars):
    plt.subplot(n_vars,1,1+i)
    
    
    dat0 = tracked_behavior['tracking_holder'][i,start_frame:(start_frame+frame_window)]
    dat1 = tracked_behavior['guessing_holder'][i,start_frame:(start_frame+frame_window)]
    
    # wrap:
    
    def wrap_angles(phases):
        phases = (phases + np.pi) % (2 * np.pi) - np.pi
        return phases
    if units[i] == 'rad' and do_wrapping:
        dat0 = wrap_angles(dat0)
        dat1 = wrap_angles(dat1)

    
    plt.plot(dat0,c=cmpl[1])
    plt.plot(dat1,c=cmpl[3])
    
    ax = plt.gca()
#     ax = plt.gca()
    plt.gca().spines['top'].set_visible(False)
    plt.gca().spines['right'].set_visible(False)
    plt.gca().spines['left'].set_visible(False)
    
    if i < (n_vars-1):

        pass
        plt.gca().spines['bottom'].set_visible(False)
        plt.xticks([])
    else:
        pass
#         adjust_spines(ax,['bottom'])

#         adjust_spines(ax,['bottom','left'])
    plt.yticks([])
    plt.ylabel(latex_vars[i])

#     plt.ylabel(tracked_behavior['var'][i])
    plt.xlim([0,frame_window+10])

    ax = plt.gca()
    ylim=ax.get_ylim()
    xlim=ax.get_xlim()
    plt.plot( -2+np.array([1,1])*xlim[1],ylim[0]+np.array([0,1])*scale_bar[i],'-k' )
    plt.text(xlim[1],ylim[0]," "+str(scale_bar[i])+' '+units[i] )


    if i < 9:
        ax.set_facecolor('k')
        ax.patch.set_alpha(0.13)
    else:
        ax.set_facecolor('peru')      
        ax.patch.set_alpha(0.2)    
    
plt.xlabel('Frames')    
plt.subplots_adjust(left=None, bottom=None, right=None, top=None, wspace=None, hspace=0)
    
# plt.tight_layout()
# plt.savefig('figure_raw_pics/figure_5/fitting_cartoon/tracked_full_pretty.pdf',transparent=False)  

plt.show()
```

In [13]:

```
from utils.analysis_tools import TrackingWrangler
```

In [14]:

```
Wrangler = TrackingWrangler(tracked_behavior,jagged_lines)
# Wrangler.kernel_smoothing()
Wrangler.unpack_all_body_support()
Wrangler.plot_body_supports()
Wrangler.unpack_body_points()
```

```
findfont: Font family ['cursive'] not found. Falling back to DejaVu Sans.
```
