# Supplementary material for: Automatic mapping of multiplexed social receptive fields by deep learning and GPU-accelerated 3D videography
Source: Nat Commun. 2022 Feb 1;13:593. doi: 10.1038/s41467-022-28153-7 (PMC8807631; doi:10.1038/s41467-022-28153-7)
Supplement: Supplementary file 9 — Supplementary Software [file 41467_2022_28153_MOESM9_ESM.zip › ebbesen_froemke_2021_code/analysis/002_Label_training_images.html]

002\_Label\_training\_images


In [1]:

```
# annontate
import numpy as np
import cv2
import h5py
import matplotlib.pyplot as plt
# from deepposekit import VideoReader, KMeansSampler
import sys
sys.path.append('/home/chrelli/git/3d_sandbox/mouseposev0p2/chrelli_annotator/')
# sys.path.append('/home/chrelli/git/3d_sandbox/mousepose_0p1/deepposekit-annotator/')

from dpk_annotator import Annotator
import tqdm
import glob
import itertools

from os.path import expanduser
home = expanduser("~")
```

# Point to the training images, label using the 'DeepPoseKit' annotator (Graving et al. 2019)¶

In [2]:

```
# The skeleton file specifies the affinity fields (nose-to-tail, ear-to-nose, etc) 
# between the clicked keypoints (nose, ears, tail, implant).

training_sets = glob.glob('training_sets' + '/*.h5')
skeletons = glob.glob('training_sets' + '/*skeleton_v2*')

print(training_sets)
print(skeletons)
```

```
['training_sets/mouse_postKonstanz_annotation_set_labeled.h5']
['training_sets/skeleton_v2_mice.csv']
```

In [3]:

```
app = Annotator(datapath=training_sets[0],
                dataset='c_images',
                skeleton=skeletons[0],
                shuffle_colors=False,
                text_scale=.3)

app.run()
```

```
/home/chrelli/git/3d_sandbox/mouseposev0p2/chrelli_annotator/dpk_annotator/gui/Annotator.py:176: H5pyDeprecationWarning: dataset.value has been deprecated. Use dataset[()] instead.
  self.image_idx = np.sum(np.all(h5file['annotated'].value, axis=1)) - 1
```

```
Saved
Saved
```

```
/home/chrelli/git/3d_sandbox/mouseposev0p2/chrelli_annotator/dpk_annotator/gui/Annotator.py:190: H5pyDeprecationWarning: The default file mode will change to 'r' (read-only) in h5py 3.0. To suppress this warning, pass the mode you need to h5py.File(), or set the global default h5.get_config().default_file_mode, or set the environment variable H5PY_DEFAULT_READONLY=1. Available modes are: 'r', 'r+', 'w', 'w-'/'x', 'a'. See the docs for details.
  with h5py.File(self.datapath) as h5file:
```
